# A progesterone derivative linked to a stable phospholipid activates breast cancer cell response without leaving the cell membrane

**DOI:** 10.1007/s00018-024-05116-3

**Published:** 2024-02-22

**Authors:** Jofre Font-Mateu, Pol Sanllehí, Jesús Sot, Beatriz Abad, Nicolas Mateos, Juan Andres Torreno-Pina, Roberto Ferrari, Roni H. G. Wright, Maria F. Garcia-Parajo, Jesús Joglar, Félix M. Goñi, Miguel Beato

**Affiliations:** 1 Center for Genomic Regulation (CRG), Barcelona Institute for Science and Technology (BIST), Barcelona, Spain; 2 Department of Biological Chemistry, Institute for Advanced Chemistry of Catalonia, IQAC-CSIC, Jordi Girona 18-26, 08034 Barcelona, Spain; 3 Instituto Biofisika (UPV/EHU, CSIC), Barrio Sarriena s/n, 48940 Leioa, Spain; 4 SGIKER, Universidad del País Vasco, Barrio Sarriena s/n, 48940 Leioa, Spain; 5 The Barcelona Institute for Science and Technology (BIST), ICFO-Institut de Ciencies Fotòniques, 08860 Barcelona, Spain; 6 Department of Chemistry, Life Sciences and Environmental Sustainability, University of Parma, Parma, Italy; 7 Faculty of Medicine and Health Sciences, Universitat Internacional de Catalunya, 08195 Sant Cugat del Vallès, Barcelona Spain; 8 ICREA, Pg. Lluis Companys 23, 08010 Barcelona, Spain; 9 Departamento de Bioquímica y Biología Molecular, Universidad del País Vasco, Barrio Sarriena s/n, 48940 Leioa, Spain; 10 Universitat Pompeu Fabra (UPF), Barcelona, Spain

**Keywords:** Progesterone receptor, Breast cancer cells, Progesterone signaling, Nuclear hormone receptors, Cell membrane phospholipids

## Abstract

In hormone-responsive breast cancer cells, progesterone (P4) has been shown to act via its nuclear receptor (nPR), a ligand-activated transcription factor. A small fraction of progesterone receptor is palmitoylated and anchored to the cell membrane (mbPR) forming a complex with estrogen receptor alpha (ERα). Upon hormone exposure, either directly or via interaction with ERα, mbPR activates the SRC/RAS/ERK kinase pathway leading to phosphorylation of nPR by ERK. Kinase activation is essential for P4 gene regulation, as the ERK and MSK1 kinases are recruited by the nPR to its genomic binding sites and trigger chromatin remodeling. An interesting open question is whether activation of mbPR can result in gene regulation in the absence of ligand binding to intracellular progesterone receptor (iPR). This matter has been investigated in the past using P4 attached to serum albumin, but the attachment is leaky and albumin can be endocytosed and degraded, liberating P4. Here, we propose a more stringent approach to address this issue by ensuring attachment of P4 to the cell membrane via covalent binding to a stable phospholipid. This strategy identifies the actions of P4 independent from hormone binding to iPR. We found that a membrane-attached progestin can activate mbPR, the ERK signaling pathway leading to iPR phosphorylation, initial gene regulation and entry into the cell cycle, in the absence of detectable intracellular progestin.

**Supplementary Information:**

The online version contains supplementary material available at 10.1007/s00018-024-05116-3.

## Introduction

The dominant view of steroid hormone action is that these amphipathic molecules diffuse through the cell membrane and interact with their intracellular receptors, members of the large nuclear receptor family, which shuttle between the cytoplasm and cell nucleus [1]. In response to binding their specific hormone ligand, the intracellular receptors dissociate from chaperones, dimerize and concentrate in the cell nucleus, where they interact with DNA sequences called hormone response elements (HRE), or with other transcription factors, and regulate the rate of transcription of their target genes [2]. Progesterone (P4) is a steroid hormone that regulates target cell behavior in many tissues via interaction with the intracellular progesterone receptor (iPR). In breast cancer cells, we have described the interaction of ligand-activated iPR with progesterone responsive elements (PREs) in chromatin and the subsequent chromatin remodeling steps required for gene regulation (for a recent review, see Beato et al. 2020) [3]. However, in addition to these well-established genomic pathways, steroid hormones are known to act via a non-genomic mechanism [4]. In breast cancer cells, estradiol activates the SRC/RAS/ERK kinase pathway via a small fraction of the nuclear estrogen receptor alpha (ERα) attached to the cell membrane (mbERα) [5]. We found that in T47D breast cancer cells this kinase pathway is also activated by progestins, via a small fraction of progesterone receptor (PR) attached to the cell membrane (mbPR) in a complex with mbERα [6]. Upon P4 exposure, the SRC/RAS/ERK kinase pathway is activated [7, 8]. It is also known that PR membrane attachment is mediated by the palmitoylation of cysteine 820, located in a short amino acid region of the ligand binding domain conserved in other steroid hormone receptors [9]. After exposure to 10 nM progestin, the mbPR remains attached to the cell membrane, while the activated ERK kinase phosphorylates iPR at serine 294, leading to its activation, displacement of its associated chaperones and dimerization. This activated phosphoPR (pPR) forms a ternary complex with activated ERK and MSK1 that binds to PREs in the nucleus and initiates the changes in chromatin and transcription factor recruitment that lead to the regulation of P4-responsive genes [7, 10]. In recent studies we found that progestins are able to activate the response of breast cancer cells at 50 pM concentration, 200-fold lower than used in most previous studies, by activating the kinase signaling pathway and promoting pPR binding to a small subset of accessible enhancers [11].

An interesting question is whether hormone entry into the cells and binding to the iPR is necessary for activating cell response to P4. It is in principle possible that hormone binding to the mbPR and subsequent kinase activation is sufficient to promote phosphorylation of the ligand-free PR, followed by dissociation of pPR from the chaperones, dimerization, binding to accessible chromatin PREs, and activation of the cell response. In the past, several groups have used steroid hormones attached to serum albumin in an attempt to demonstrate that the hormones can act without crossing the cell membrane [12, 13]. However, this strategy is not convincing for two main reasons: the attachment of steroids to albumin is non-covalent, and albumin can be endocytosed and degraded by lysosomes leading to release of P4 into the cytosol. Here we report an original and more stringent approach to ensure that the hormone remains firmly attached to the cell membrane during the experimental procedure, allowing the study of those hormonal functions, if any, that only require ligand binding to the mbPR. To this end, we attached a P4 derivative via a linker chain to the polar head group of a phosphatidylcholine analogue, an *Archaea-*like phospholipid with two fatty acids linked to glycerol by ether bonds that cannot be cleaved by cellular phospholipases A2 [14]. Therefore, the steroid would be stably anchored to the membrane via the alkyl phospholipid, and the linker chain (4–8 ethylene oxide units) would provide space for the anchored steroid to reach the mbPR binding site [15]. In addition, the lipid was fluorescently labeled in one of the alkyl chains to allow detection within the cells [16]. The results show that our approach works in principle, as the membrane-attached P4 derivative activates the cell response to hormone, without entering the cell. This strategy may help in the study of the proteins associated with membrane-bound hormone receptors and their interactions with protein kinases. In addition, this study underscores the physiological importance of low levels of hormone receptors attached to the cell membrane in breast cancer, which opens the possibility to target them for blocking kinase activation as part of the endocrine therapy of cancer.

## Results

### Design and synthesis of the probes

**Fig. 1:**
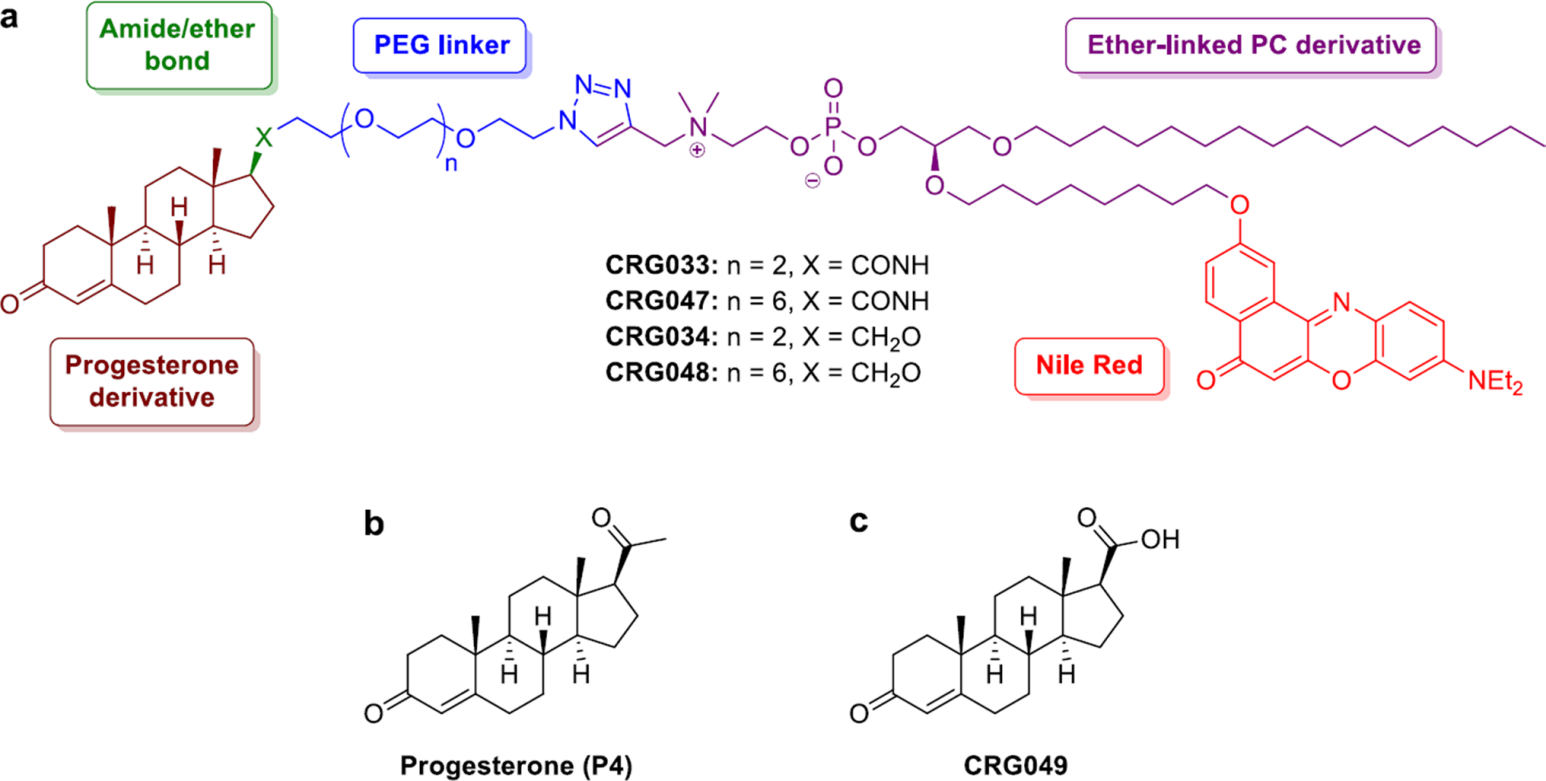
Chemical structure of synthesized compounds. **a** Structure of the designed membrane-binding PR probes CRG033, CRG047, CRG0 [35] and CRG048. **b** Structure of progesterone P4. **c** Structure of compound CRG049, the hydrolysis byproduct of probes CRG033 and CRG047

The probes used in this work (CRG033, CRG047, CRG034 and CRG048) consist of a progesterone derivative attached to the polar head group of a chemically modified lipid by means of a polyethylene glycol (PEG) linker of variable length (Fig. 1).

For a full account of the synthetic experimental part see the Supporting Information. Briefly, a Cu(I)-catalyzed azide-alkyne [3 + 2] dipolar cycloaddition (CuAAC) between an azide-terminated moiety bearing the progesterone derivative and an alkyne-containing lipid was envisaged as a suitable and efficient way to synthetically ensemble the targeted probes [17, 18]. To provide a stable anchor to the cell membrane, an ether-linked phosphatidylcholine derivative was used as the lipidic moiety of the probes. As opposed to ester-linked lipids, in this *Archaea*-like phospholipid structure the two acyl chains were linked to the glycerol by ether bonds, which conferred an increased metabolic stability against cellular phospholipases A2 [14].

In addition, the lipid was fluorescently labeled in one of the alkyl chains in order to allow probe detection during in vitro and in vivo experiments [16]. Nile Red was chosen as a fluorophore. Under physiological conditions, Nile Red is an uncharged, low-polar molecule, which facilitates dye insertion into the cell membrane. Hence, a 2-hydroxy Nile-Red derivative was placed at the distal position of one of the lipid alkyl chains via an ether bond (Supplementary Information Scheme S4) [19–21].

As previously mentioned, a PEG linker was connecting the steroidal and lipid moieties of the probes. Insertion of a short-chain PEG conferred water solubility to the probes and increased their amphipathic nature [15]. In this case, we reasoned that linkers with either four (CRG033 and CRG034) or eight (CRG047 and CRG048) ethylene oxide units would provide enough space for the progesterone derivative to reach the mbPR binding site and exert its activity (Supplementary Information Scheme S1).

Since the C3 ketone functionality is known to be crucial for PR activation [22], the progesterone fragment was attached to the probe at the 17β position of the steroidal scaffold. In compounds CRG033 and CRG047, the steroid was anchored to the linker via an amide bond, which preserved the carbonyl functionality of the natural ligand but could be susceptible of metabolic degradation by amidases. Thus, despite not bearing the C20 carbonyl group of progesterone (Fig. 1), the chemically and metabolically more stable ether-containing probes CRG034 and CRG048 were also prepared (Supplementary Information Schemes S2, S3 and S4).

### Affinity of the synthesized compounds for PR

**Fig. 2:**
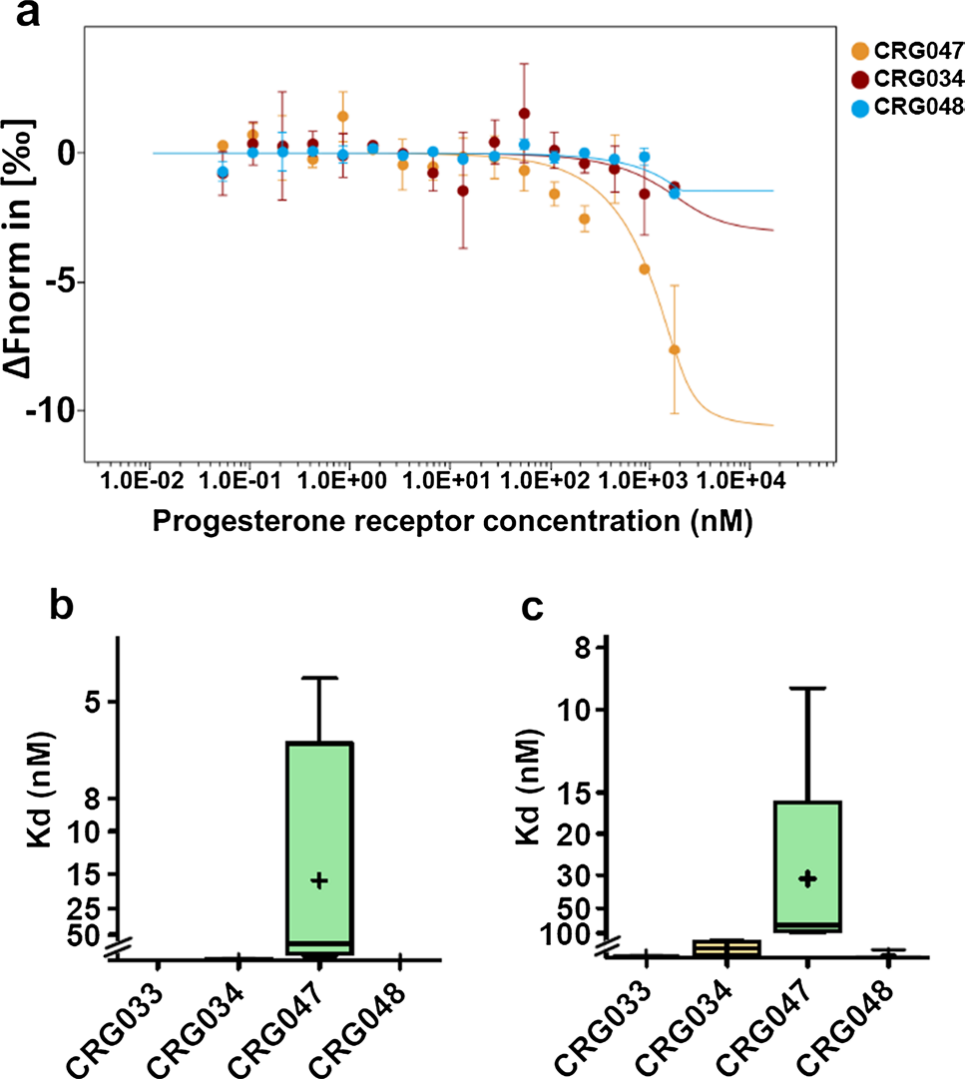
PR affinity with progesterone-like compounds. **a** Microscale thermophoresis (MST). The thermophoresis changes as normalized fluorescence (ΔFnorm in [‰]) is plotted against the concentration of the unlabeled protein and fitted according to the law of mass action. Average ± SD of two readings from the same experiment is shown. (**b**, **c**) Microscale thermophoresis (MST) analysis of 2 μM progesterone-like compounds with increasing concentrations of full-length progesterone receptor expressed in baculovirus (**b**) or GST-progesterone receptor Ligand-binding Domain (LBD) (**c**). Internal fluorescence of the Nile-red moiety in the compounds was used for the detection of the binding affinities. Due to precipitation, signal saturation was not reached. Boxplots show results from at least four independent experiments. ‘ + ’ indicates average (CRG047 Kd for full PR, 16.1 nM; for PR-LBD, 31.2 nM)

The chemical structure of several of the synthesized compounds and of a possible product of the hydrolysis of the amide bond of CRG033 or CRG047 (labeled CRG049) are shown in Fig. 1. After their chemical and fluorometric characterization (see Supporting Information, Fig. S1 and Table S1), we first explored their affinity for purified recombinant full-length PR expressed in baculovirus [23] using microscale thermophoresis (MST, see Experimental Section for details). Among tested compounds, only CRG047 exhibited a Kd for PR (≈ 14 nM) at the same low nM range as P4 [24] (Fig. 2a, b and Fig. S2a–d). A comparable affinity was found using the recombinant ligand-binding domain of PR expressed in *E. coli* [25] (Fig. 2c). We therefore chose CRG047 for subsequent characterization.

### CRG047 in lipid bilayers

**Fig. 3:**
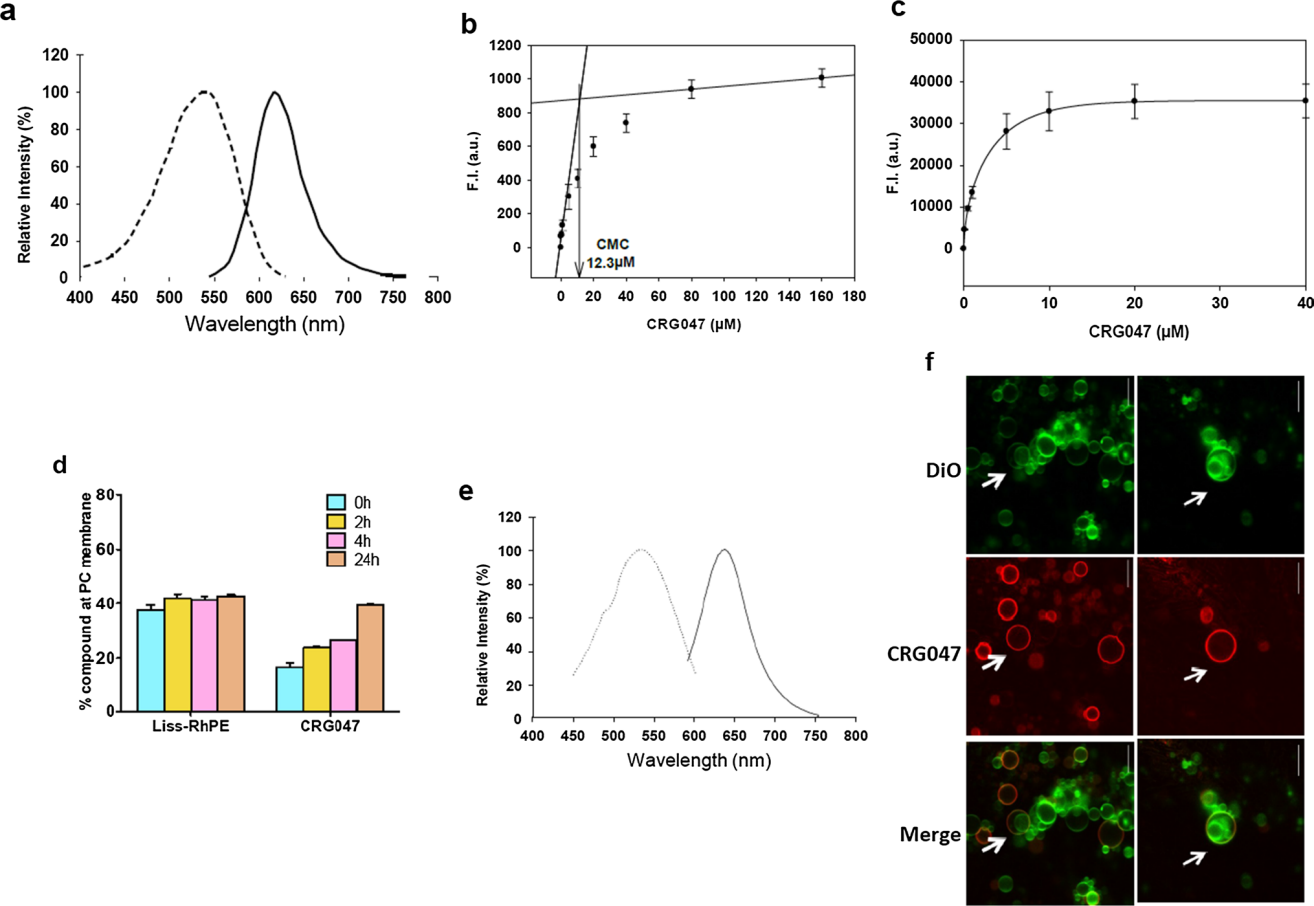
Biophysical properties of the CRG047 probe. **a** Excitation and emission spectra of CRG047 in ethanol. **b** Critical micellar concentration (CMC) of CRG047, measured by fluorescence spectroscopy. **c** Dose-dependent fluorescence emission of CRG047 in small unilamellar vesicles (SUV) composed of egg PC. **d** CRG047 incorporation into egg PC bilayers (SUV) as a function of time, and a comparison with the incorporation of Liss-RhPE as indicated. **e** Excitation and emission spectra of CRG047 in SUV composed of egg PC. **f** Incorporation of CRG047 into vesicles found inside mostly unilamellar GUV composed of egg PC. Two representative fields are shown. Top: Vesicles stained with DiO during the formation of the vesicles. Middle: CRG047 stain. Bottom: merge. The arrows point to internal vesicles within the larger GUV. CRG047 cannot diffuse through the outer bilayers towards the inner vesicles. Data in b, c, and d are average values ± SD

The fluorescence properties of CRG047 in organic solvent (ethanol) are summarized in Fig. 3a. For measuring the critical micellar concentration (CMC) of CRG047, increasing concentrations of the compound dissolved in DMSO were added to 25 mM HEPES, 150 mM NaCl, pH 7.4, and the increase in emitted fluorescence was measured to estimate its CMC, that was of ≈ 12 μM (Fig. 3b).

To study the CRG047 affinity for phospholipid membranes, increasing amounts of the compound in DMSO were added to a suspension of small unilamellar vesicles (SUV), and the fluorescence was recorded. Fluorescence intensity increased until saturation was reached (at ≈ 20 µM under our conditions) (Fig. 3c). The lower this concentration, the higher is the affinity of the compound for the vesicles. The time increase of bound CRG047 after mixing (Fig. 3d) indicated that the compound did not leave the membranes for several hours, and that additional compound was gradually transferred from micelles to membrane bilayers (Fig. 3d). CRG047 contains both hydrophobic (PC-derivative + progesterone derivative) and hydrophilic (PEG linker) moieties, i.e., it is an amphipathic molecule. This leads to the compound existing initially in equilibrium between the lipid and water phases, but finally being transferred to the non-polar lipid phase, for which it appears to have some preference. In contrast with the amphipathic CRG047, the less polar Liss-RhPE reached equilibrium with the bilayers in the first minutes (Fig. 3d). The excitation and emission spectra of CRG047 (in SUVs) are shown in Fig. 3e.

The topology of CRG047 in membranes can be inferred from studies on the partition of amphipathic molecules into membranes (see e.g., review in Lichtenberg et al. [26]). It can be presumed that both non-polar moieties (PC-derivative + progesterone derivative) will diffuse across the membrane hydrophobic core, and reach, at least in part, the inner monolayer, thus having access to the mbPR. In contrast, the polar PEG linker moiety would remain outside the membrane. It cannot be ruled out, however, that the PEG moiety can also move across the membrane, driven by the motion of the non-polar components; in any case the progesterone moiety would be unable to diffuse through the cytosol, because of the phospholipid anchor.

We also used fluorescence microscopy to explore the incorporation and retention of CRG047 (Fig. 3f) into the membranes of preformed giant vesicles (GUV). For this purpose GUV were electroformed containing DiO (a hydrophobic probe different from Liss-RhPE), shown in green in Fig. 3f. The resulting GUV were oligolamellar, i.e., some of them contained other smaller vesicles inside (Fig. 3f top). When these already formed GUV were treated with externally added CRG047 (in red, Fig. 3f middle), only the larger, outer vesicles were stained, showing that CRG047 cannot diffuse across the membranes. CRG047 remained in the outermost vesicle membrane and was unable to diffuse across the bilayers (white arrows) to reach inner vesicles. This shows the suitability of the phospholipid-bound progestin to be used in live-cell experiments.

### CRG047 interaction with breast cancer cells membrane and activation of the kinase cascade

**Fig. 4:**
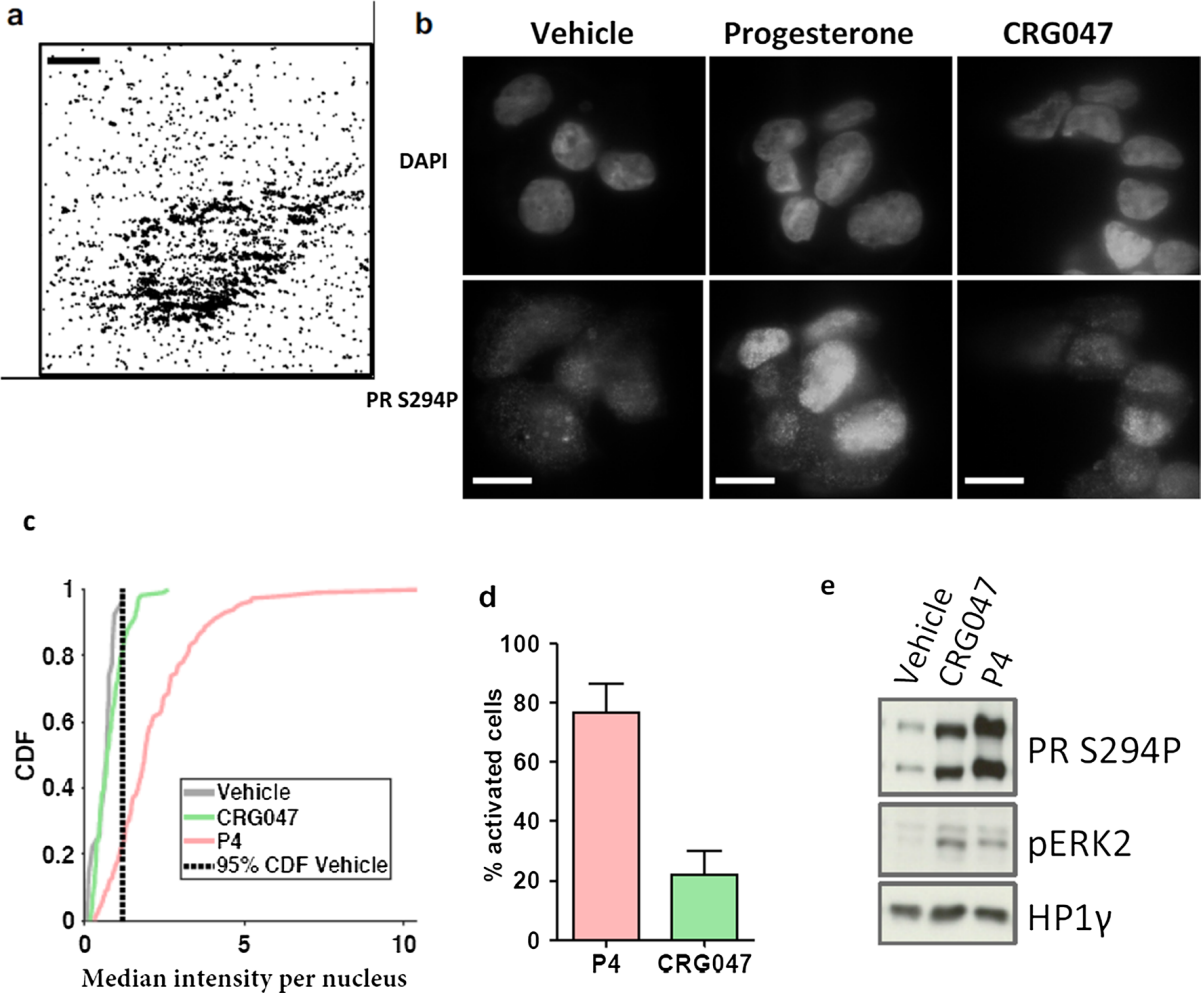
CRG047 interacts with and diffuses along the plasma cell membrane in T47D cells and activates MAPK pathway. **a** High density map of the Nile-Red signals found in the movie (Fig. S3), showing the accumulation of fluorescence around the cell membrane in contact with the glass surface. Scale bar: 20 μm. **b** Immunostaining. T47D cells imaged under HILO illumination in hormone-free conditions were exposed to vehicle, P4 or CRG047 as indicated. Upper panel: staining with DAPI for nuclei identification. Lower panel: PR Ser294 phosphorylation (PR S294P) signal after 30 min of exposure for each of the conditions. Scale bar: 10 μm. **c** Cumulative density function (CDF) of the median intensity per nucleus for vehicle (grey), CRG047 (green), and P4 (magenta). The dashed line depicts 95% CDF for the vehicle condition. The nuclei with a median intensity above this value are therefore considered as activated by the respective compound. The intensity is in arbitrary units. A total of 27 cells were analyzed for vehicle, 117 cells for P4 condition and 116 cells for CRG047 condition. **d** Histogram showing the percentage of cells activated upon exposure to progesterone or CRG047 (77% for P4; 22% for CRG047). Error bars indicate SD. **e** Western blot on T47D extracts. T47D cells were treated with either vehicle, P4 or CRG047 (in the presence of 0.001% Tween20). Upper panel: blot against PR Ser 294 phosphorylation. Middle panel: blot against ERK2 p42/p44 phosphorylation. Lower panel: blot against HP1g as loading control

To further assess CRG047 incorporation to the cell membrane of living breast cancer cells, we incubated T47D cells with a physiologically relevant 10 nM concentration of CRG047 in PBS for 30 min. We visualized the incorporation of CRG047 by means of single molecule imaging of the fluorescent organic dye Nile Red under total internal reflection fluorescence microscopy (TIRF). We observed that the incorporation of CRG047 at such low concentrations yielded single molecules on the cell membrane of living T47D cells (see attached Video 1, Supplementary Fig. S3). To efficiently visualize the incorporation of CRG047, we performed high-density single molecule maps (Fig. 4a) of Nile Red, reflecting not only that CRG047 is incorporated on the cell membrane but that it also diffuses laterally as expected from a lipid bilayer intercalating dye.

We proceeded to assess the activation of the MAPK signaling cascade exposing T47D cells to P4 or CRG047 compared to vehicle. We first tested the activation of ERK kinase activated phosphorylated PRS294P by means of a specific antibody using highly inclined and laminated optical sheet (HILO) fluorescence microscopy of an AlexaFluor 488 labeled secondary antibody. A significant fraction of cells showed a punctated fluorescence pattern characteristic of PR activation (Fig. 4b). By calculating the cumulative density function (CDF) of the fluorescence intensity per nucleus of the antibody PRS294P (Fig. 4c), we determined that 77% or 22% of the cells were activated when exposed to 10 nM P4 or CRG047, respectively (Fig. 4d). We confirmed in cell extracts both ERK1/2 activation and PR phosphorylation when the cells were exposed to P4 or CRG047 (Fig. 4e). Thus, these experiments show that the compound CRG047 binds to the cellular membrane of the breast cancer cells and is able to activate the ERK-dependent PR signaling cascade.

### Functional response of the cells: gene regulation and cell proliferation through PR-chromatin interaction

**Fig. 5:**
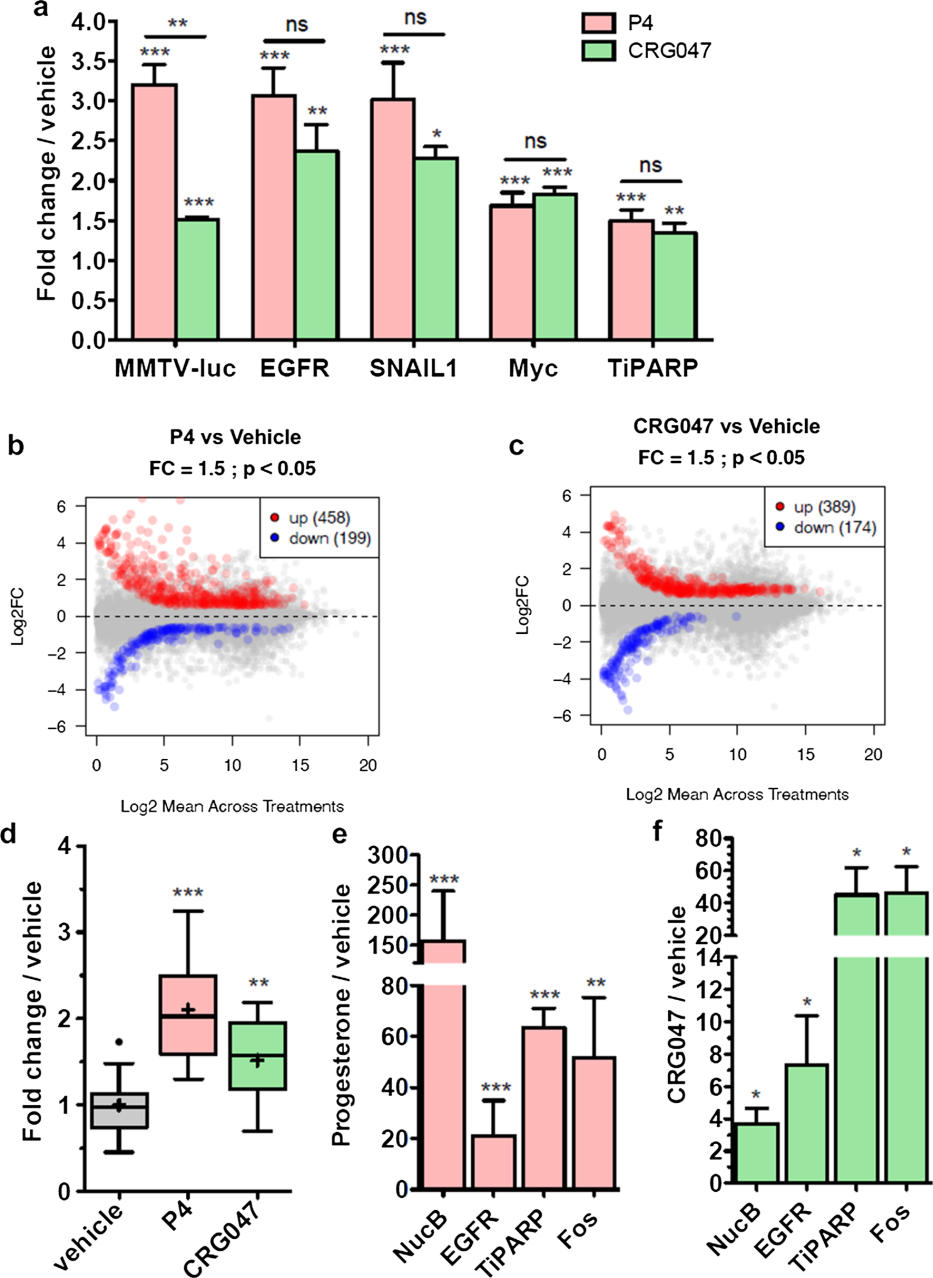
CRG047 activates transcription and proliferation through targeting PR to its HRE. For all graphs in this figure: All statistical analyses in this panel were done by 1 tail t test comparing the indicated bar with vehicle treated samples or as indicated. *p value < 0.05; **p value < 0.01; ***p value < 0.001; ns: *p* value > 0.05. **a** RNA induction in T47D cells. Expression of progesterone target genes was analyzed by qRT-PCR. T47D cells in hormone-free conditions were exposed to vehicle, P4 or CRG047, RNA was extracted and qPCR analysis was performed for the indicated genes. For each gene, vehicle average levels were set to 1 and numbers indicate fold change over vehicle average. Values represent average ± SD of 3 independent experiments. **b** Scatter plot of Pol II gene expression in T47D cells exposed to P4 and vehicle. The number of genes up- or down-regulated in the P4 condition (− 1.5 < FC > 1.5; adjusted *p* value < 0.05) is indicated. **c** Scatter plot of Pol II gene expression in T47D cells exposed to CRG047 and vehicle. The number of genes up- or down-regulated in the CRG047 condition (− 1.5 < FC > 1.5; adjusted *p* value < 0.05) is indicated. **d** Cell proliferation was measured by BrdU incorporation in T47D cells following the addition of vehicle, P4 or CRG047. Boxplots represent the average of 12 independent experiments. Dot indicates outlayer.’ + ’ indicates average. **e**, **f** ChIP against Progesterone receptor upon P4 (**e**) or CRG047 (f) induction of T47D cells. Values represent averages of three independent experiments ± SD

To evaluate the nuclear response to the membrane-attached progestin, we used qRT-PCR to measure mRNA levels for genes known to be activated by P4, including *cMyc*, *Snail1*, *TiPARP,* and *EGFR,* as well as the chromosomally integrated reporter *MMTV-luc*. All these genes were significantly activated by 10 nM P4 and also by 10 nM CRG047 (Fig. 5a).

We therefore performed a whole gene expression analysis of poly(A)-RNA by RNA-seq in cells exposed to 10 nM of either P4 or CRG047. After exposure to 10 nM P4, 458 genes were up-regulated and 199 were down-regulated, while upon exposure to 10 nM CRG047, 389 genes were up-regulated and 174 were down-regulated (Fig. 5b, c). This indicates that CRG047 regulates a large number of genes comparable to those regulated by P4, though the extent of the regulation by CRG047 was less pronounced (Fig. S4b, right panel). Note however that, in the absence of gene identities, it would be difficult to ascertain that both chemicals have similar effects on the cellular gene expression pattern. Even if a number of genes were activated/repressed by CRG047, neither GO nor KEGG Pathway analysis revealed any clear patterns of gene expression.

As a further indicator of biological response to progestin, we measured the incorporation of BrdU into DNA, an indicator of cells entering the S phase of the cell cycle. We shortly exposed T47D cells to 10 nM of either P4, CRG047 or vehicle, followed by incubation in hormone-free medium. We found that both P4 and CRG047 significantly activated DNA synthesis compared to vehicle exposure (Fig. 5d).

To explore whether this gene regulation by CRG047 was mediated by binding of PR to its known HREs near regulated genes, we performed ChIP-qPCR at P4 regulated genes after exposure to 10 nM P4 or CRG047. We found a significant binding of PR to the regulatory PREs in five P4 responsive genes including the promoter region of the MMTV- luc reporter (NucB). The effects of CRG047 were in the same direction than those of P4 (Fig. 5e for P4 and f for CRG047). Therefore, we conclude that the progestin-like compound can activate the cell response in a similar manner as progesterone.

### Neither CRG047 nor its putative hydrolysis product are detected within cell nuclei

To explore whether CRG047 did enter the cell nuclei, T47D cells were exposed shortly to 10 or 100 nM P4 or CRG047 and total cell extracts and nuclear extracts were subjected to quantitative mass spectrometry (MS). Analyses were performed in parallel for CRG047 (Fig. S5a–d) or P4 (Fig. S5e–h). Although the limit of detection was below 1 pmol (not shown), neither CRG047 nor P4 could be detected in nuclear extracts (compare panels a, b for CRG047 and panels e, f for P4). Similar analyses were performed with the progesterone analogue R5020 (promegestone) (panels i-l).

To exclude that CRG049—the steroid metabolite of CRG047 hydrolysis – could be responsible for some of the observed effects, the levels of CRG047 and CRG049 in total extracts from cells exposed to 10 nM and 100 nM CRG047 were measured by MS. The results are shown in Fig. S6. While CRG047 was clearly detected in the cell extracts (Fig. S6a), CRG049 was indistinguishable from noise at the same concentrations both in nuclear and total cell extracts (Fig. S6a). Thus, it seems unlikely that the hydrolysis of CRG047 was responsible for the observed effects in breast cancer cell function.

### Palmitoylated PR at Cys 820 is required for the response to CRG047

**Fig. 6:**
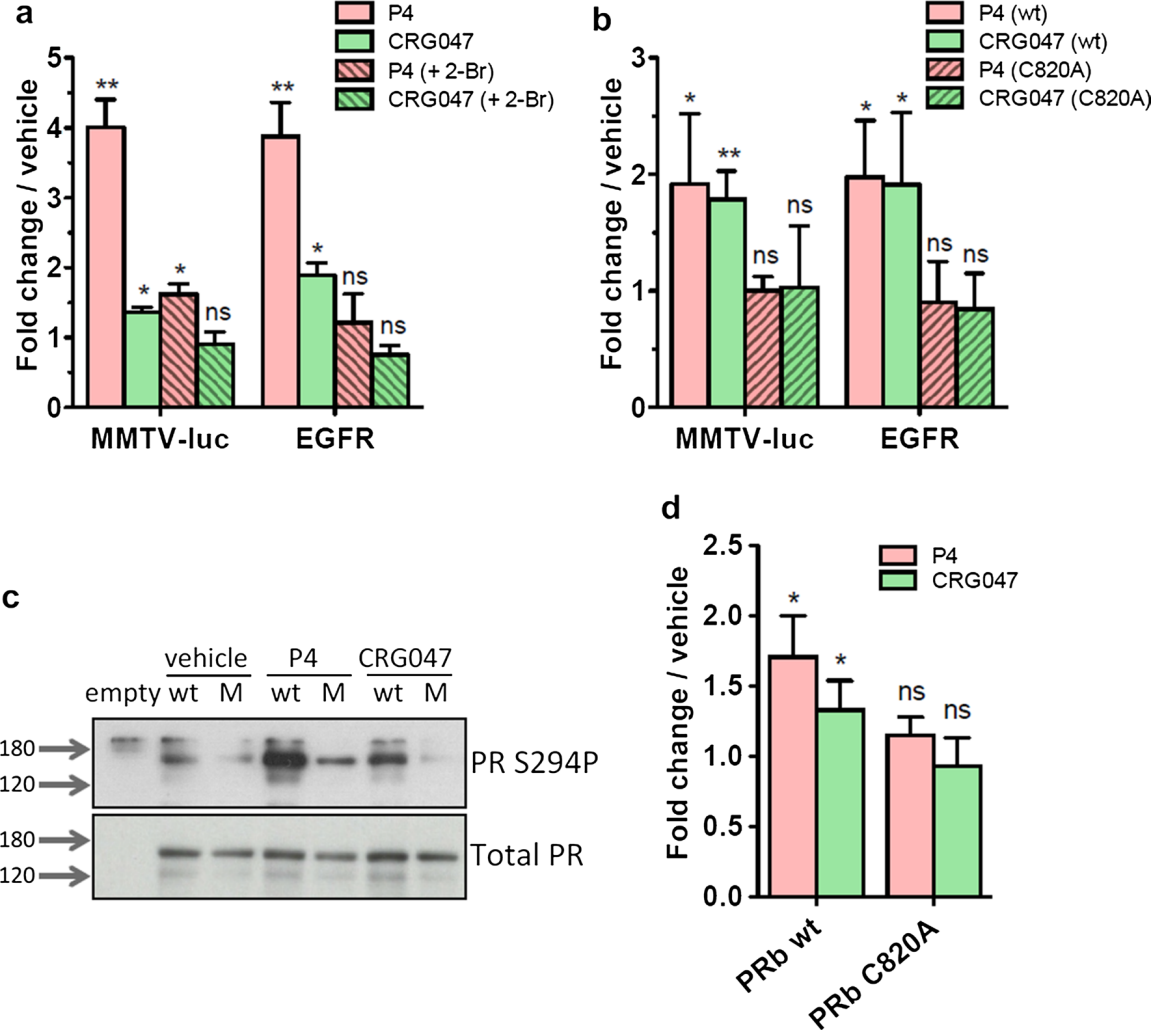
CRG047 action is dependent on PR palmitoylation. For all graphs in this figure: Statistical analyses in this panel were done by 1 tail t test comparing the indicated induction (P4 or CRG047) with vehicle in triplicates. *p value < 0.05; **p value < 0.01; ns: *p* value > 0.05. **a** Palmitoylation inhibition. T47D cells were incubated for 6 h with 2- bromopalmitate 10 μM (2-Br, dashed bars) prior to induction with the indicated compounds for 3 h. RNA signal for indicated genes is shown relative to vehicle induction. Bars represent the average ± SD of three independent experiments. **b** PR-negative breast cancer cell line T47D-Y stably expressing a single copy of the MMTV promoter (TYML) were transfected with wild type PRb (solid bars) or the palmitoylation mutant PRbC820A (dashed bars) plasmids and incubated with P4 or CRG047 as indicated. Average ± SD of three independent experiments is shown. Fold changes are relative to the average response of cells incubated with vehicle. **c** Western-blot against PR Ser294 phosphorylation (upper panel) or total progesterone receptor as loading control (lower panel). U2OS cells were transfected with empty plasmid, or with plasmids for the expression of either wild type PRb (wt) or the palmitoylation mutant PRb C820A (M) and incubated with vehicle, P4 or CRG047. Fold changes are relative to the average response of cells incubated with vehicle. **d** Histogram of three independent experiments (average ± SD) performed as in panel c. Signal was captured by pixel density and normalized against total PR signal as loading control

Given that other P4 binding proteins are known to inhabit the cell membrane [27], in order to ensure that the mbPR was responsible for the action of CRG047, we inhibited palmitoylation with 10 µM 2-bromopalmitate (2-Br) [9]. We found that this treatment effectively blocks the response.

of T47D to both P4 and CRG047 (Fig. 6a). In addition, we used a PR mutant in which the palmitoylation site (Cys-820) was mutated to Ala (PR C820A). This mutant PR does not integrate into the cell membrane [9]. We found that, when expressed in T47D cells deprived of wtPR that express a single MMTV promoter copy (TYML) [28], PR C820A could not mediate the cell response to 10 nM P4 or CRG047 in terms of activation of P4- responsive genes (Fig. 6b and Fig. S7a). Similar results were obtained when wtPR or PR C820A mutant were co-expressed along with the MMTV promoter plasmid in another cancer cell line defective in PR (U2OS cells) and exposed to P4 or CRG047 (Fig. S7b). In addition, with U2OS cells expressing either the wtPR or the mutant PR C820A, only the wtPR-expressing cells activated the ERK kinase and led to PR phosphorylation (Fig. 6c and d).

It could be argued that palmitoylation inhibitors would inhibit SRC/RAS association with the membrane, thus they should not be used as a valid evidence to support a role of PR palmitoylation. However, blocking palmitoylation during exposure to progesterone or CRG047 should have a strong effect on PR palmitoylation and a much smaller one on SRC/RAS palmitoylation, which is not activated by progesterone. We conclude that CRG047 action on T47D cells require the anchoring of PR to the cell membrane via palmitoylation.

## Discussion

The purpose of this work was to explore whether a membrane-attached progestin derivative could activate the cellular response of breast cancer cells to progestins without the requirement of the hormone penetrating into the cell and binding the intracellular PR. The results are compatible with this hypothesis, although we do not exclude other possible explanations.

In support of our hypothesis, the above results show that the progestin linked to a stable phospholipid (CRG047) binds the mbPR with high affinity and remains attached to the cell membrane during the time of exposure of the cells (30 min). Chemically, the amide bond linking progestin to the phospholipid is not hydrolyzed during exposure of the cells to CRG047 as the resulting compound (CRG049) cannot be detected in the cell extracts. Our designed compound CRG047 induces the activation of the ERK kinase pathway leading to phosphorylation of the iPR at S294, which results in PR binding to genomic PREs, regulation of the associated progesterone sensitive genes, and entry into the S phase of the cell cycle. Finally, our data show that anchoring of PR to the cell membrane via palmitoylation at C820 is essential for CRG047 action.

In fact, it has never been demonstrated that the PR that binds to chromatin PREs upon exposure of breast cancer cells to 10 nM or to 50 pM P4 is actually carrying a bound hormone ligand [11, 29], and we cannot demonstrate the presence of P4 in nuclear extracts of cells exposed to 10 nM P4. Thus, the main function of the hormone ligand could be to activate the kinase pathway via mbPR, leading to phosphorylation of the intracellular PR, detachment from chaperones, dimerization and transfer to the cell nucleus.

One limitation of our study is that our assays are focused on the initial response of the cells to hormone, as we did not dare to expose the cells to CRG047 for longer than 30 min. The reason was that we wanted to exclude the possibility that the mbPR could be reaching the cell interior during the metabolic processing of the membrane. This short exposure time may also be responsible for the low percentage of cells (~ 20%) that responded to CRG047, since judging from the experiments with phospholipid vesicles, binding of CRG047 increased after longer incubation times, most likely due to the fraction of compound forming micelles (not available for binding to the cell membranes) is only gradually being released. Nevertheless, even this short exposure time was sufficient to initiate the cell cycle in the next 16 h. Note that, in classical experiments with progesterone, the effects are measured after exposure to nM steroid concentrations for longer times (hours). Then, a large fraction of cells respond to the hormone. In this study we used lower concentrations and shorter exposure times to avoid membrane degradation or formation of membrane vesicles, this is probably the reason why only 20% of the cells responded.

A further limitation of our study is that we have not explored the binding of the compound CRG047 to other known membrane-attached progestin binding proteins [28]. PAQRs are seven-transmembrane-helix proteins that bind progesterone and adiponectin Q, and are expressed in many tissues including the nervous system. To our knowledge, these PAQRs receptors are not expressed in the T47D cell lines we have used for this study. Progesterone Membrane Components 1 and 2 (PGRMC1/2) are single- pass transmembrane receptors that bind progesterone, cortisol, androgens, cholesterol and heme [30]. We have not explored the possible function of PGRMCs in response to progestins, but we have shown through the palmitoylation of PR mutants that the effects of CRG047 require the binding of PR to the membrane.

Finally, the strategy used in this study has not been tested in experiments in living animals, as the phospholipid-containing compounds we used may bind many components of the blood and other fluids, making difficult their interaction with the target cells at the required low concentrations.

In any case, the relevance of the mbPR for the hormone response suggests that it could be a novel target for endocrine therapy of breast cancers. For instance, developing selective inhibitors of its palmitoylation, or blocking its interaction with the SRC kinase. It is very likely that ERα is bound to the cell membrane by a similar mechanism, since it shares the conserved palmitoylation signal [9]. Thus, a selective palmitoylation inhibitor would block the action of both estrogens and progestins. This strategy would require a better understanding of the complex formed by mbPR with ERα, the SRC kinase, and other associated proteins.

## Materials and methods

### Materials

Progesterone and R5020 were obtained from Perkin-Elmer. Lipofectamine 2000 Transfection reagent was from Thermo Fisher. 2-Bromopalmitate was acquired from Sigma. pcDNA3.1 ( +) SNAP-GFP-PRb wt and pcDNA3.1 ( +) SNAP-GFP-PRb C820A plasmids were generated from pTET-Splice SNAP-GFP-PRb plasmid kindly given by Gordon Hager, subcloned into pcDNA3.1( +) backbone. Mutagenesis of the palmitoylation site (aa Cys 820 into Ala) was done by GenScript Biotech (Netherlands) B.V. pAGEMMTVLu(MMTV-luc) was used to assess expression of the progesterone responsive promoter MMTV in U2OS cells.

### Compounds synthesis

For a full account of the synthesis of all new compounds used in this work see Supporting Information.

### Cell culture

T47D cells encompassing a single copy of the MMTV promoter were grown in RPMI 1640 (Gibco) supplemented with 10% fetal bovine serum (FBS), 100 U/ml penicillin, 100 µg/ml streptomycin, 2 mM L-glutamine and 10 µg/ml insulin. TYML cells and U2OS cells were grown in DMEM (Gibco) supplemented with 10% fetal bovine serum (FBS), 100 U/ml penicillin and 100 µg/ml streptomycin.

### Hormone induction

For hormone induction experiments, cells were plated in DMEM/RPMI media without phenol red, supplemented with 10% dextran-coated charcoal-treated FBS and incubated for 48 h. Cells were synchronized 16 h prior to hormone induction by starvation in serum-free medium.

For inductions, cells were incubated with 10 nM P4, 10 nM CRG047 or vehicle (ethanol) as indicated. Progesterone or progesterone-like compounds were resuspended in ethanol to 2 mM concentrations, and diluted with PBS to the indicated concentrations for cellular induction. Vehicle exposed cells were treated in the same manner relative to ethanol final concentrations.

For longer time points, hormone dilutions were replaced by fresh hormone-free media after 30 min of induction.

### Transient transfections

Wild type and mutant PR constructs were transfected into TYML or U2OS cells using Lipofectamine 2000 Transfection reagent (Thermo Fischer) following manufacturer’s instructions. 300.000 cells were plated in 35 mm wells and transfected with 0.5 µg of plasmid/well. U2OS cells were co-transfected with MMTV-luc plasmid for expression inducing experiments. Hormone induction experiments were carried 48 h post-transfection.

### Palmitoylation inhibitor

T47D cells were exposed to 10 μM 2-bromopalmitate (Sigma) for 6 h prior to hormone induction.

### Microscale thermophoresis (MST)

Histidine-tagged Progesterone Receptor (PR) B was purified as previously described [23] with a modification. Activation with a progesterone analog was skipped in order to avoid competition with the compounds to be analyzed. Progesterone-like compounds affinity for Progesterone Receptor was measured by Microscale Thermophoresis (MST). Non-labeled His-PRb recombinant protein or GST-PR Ligand Binding Domain (LBD), purified as described [25] (starting concentration 1750 nM), were titrated to a fixed concentration of Nile-Red-containing progesterone derivative compounds (2 µM) in a buffer containing 20 mM Tris pH 7.4, 90 mM NaCl, 1 mM DTT, 10% glycerol and 0.01% Tween 20. MST data was recorded at 20 °C using the blue LED at 10% and IR-Laser at 40% according to manufacturer’s instructions. The thermophoresis change as normalized fluorescence (ΔFnorm in [‰]) was plotted against the concentration of the unlabeled protein and fitted according to the law of mass action.

### Fluorescence spectroscopy measurements

Lipids were mixed in chloroform:methanol (2:1), and the solvent was evaporated to dryness under a stream of N2. Then the sample was kept under vacuum for 2 h to remove solvent traces, and the lipids were swollen in the appropriate buffer, 25 mM HEPES, 150 mM NaCl, pH 7.4. Small unilamellar vesicles (SUV) were obtained by sonicating the swollen lipid suspensions with a probe-type Soniprep 150 sonicator (MSK, London, U.K.) [31]. The lipid concentration was determined by phosphate analysis [32]. SUV were mixed with CRG047, and the mixture was left to equilibrate (usually 1 h). Fluorescence was measured in a QuantaMaster 40 spectrofluorometer (Photon Technology International, Lawrenceville, NJ) (λex = 550 nm, λem = 625 nm). The measurements were performed at a constant temperature of 22 °C. The extinction coefficient and quantum yield of CRG047 were obtained as described. [16]

### Confocal microscopy

Giant unilamellar vesicles (GUV) were prepared by electroformation on a pair of platinum (Pt) wires by a method first developed by Angelova and Dimitrov [33], modified as described previously [34]. Lipid stock solutions were prepared in 2:1 (v/v) chloroform/methanol at 0.2 mg/ml, and appropriate volumes of each one were mixed. Labelling was carried out by pre-mixing the desired fluorescent probes with the lipids in organic solvent. 3,3′-Dioctadecyloxacarbocyanine Perchlorate (DiO) was used as a general marker for lipid membrane. The average concentration of individual fluorescent probes in each sample was 0.5 mol%. 2.5 μl organic solutions of lipid mixtures containing the fluorescent probes were deposited on Pt wires. The Pt wires were placed under vacuum for 2 h to completely remove the organic solvent.

One side of the chamber was then sealed with a coverslip. 500 µl assay buffer, prepared with high-purity water (Millipore SuperQ) heated at 37 °C was added to the chamber until it covered the Pt wires, and connected to a TG330 function generator (Thurlby Thandar Instruments, Huntingdon, UK). AC field was applied in three steps, all of them performed at 37 °C: (1) frequency 500 Hz, amplitude 220 mV (35 V/m) for 5 min; (2) frequency 500 Hz, amplitude 1900 mV (313 V/m) for 20 min; (3) frequency 500 Hz, amplitude 5.3 V (870 V/m) for 90 min. The temperatures used for GUV formation correspond to those at which the different membranes display a single fluid phase.

GUVs were imaged with confocal microscopy using a Nikon D-eclipse C1 confocal system (Nikon corporation, Tokyo, Japan). The excitation wavelengths used for excitation were 488 nm for DiO and 561 nm for CRG047. Fluorescence emission was retrieved at 500–530 nm for DiO and at 573–613 nm for CRG047. When required, 2 µM CRG047 in DMSO was added to study its effect on the GUVs. All these experiments were carried out at 22 °C. Image treatment was performed using EZ-C1 3.20 software (Nikon Inc., Melville, NY).

### Immunostaining

Cells were plated in chambered cover glass. After induction, cells were fixed with 3% PFA in PBS for 10 min at RT, rinsed three times with PBS and blocked with 3% BSA, 0.2% Triton X-100 in PBS for 1 h. Primary antibody incubation was done in blocking buffer for 1 h at rt (anti PR Ser294Phospho, ab61785, Abcam). Primary antibody was washed three times in wash buffer (0.2% BSA, 0.05% Triton X-100 in PBS) and secondary (Alexa fluor 488 antibody, Thermo Fisher #A-11008) was added in blocking buffer for 40 min. Cells were washed again three times and DAPI was added for 1 min in a 10.000 × dilution in PBS and washed three times.

### Single molecule sensitive fluorescence microscopy

HILO or TIRF imaging were performed in a Nikon N-STORM 4.0 microscope system equipped with a TIRF 100x, 1.49 numerical aperture objective (Nikon, CFI SR HP Apochromat TIRF 100XC Oil). The dyes Nile Red or Alexa Fluor 488 were excited with a continuous 561 nm or 488 nm laser line, respectively. The emission fluorescence was collected and projected into an EM-CCD Andor Ixon Ultra Camera at a frame rate of 15 ms. The pixel size of the camera is 160 nm. High density maps were produced by collapsing (≈ 7700) single molecule localizations of Nile-Red labeled CRG047, incorporated on the cell membrane of a living T47D cell, into 1 single frame.

### Immunostaining quantification methodology. Quantification of activated cells using MATLAB

To quantify the percentage of activated cells under different conditions, we measured the median intensity of the fluorescence emission of each nucleus (Fig. S8a). to obtain such fluorescence intensity values, we first took the DAPI images (Fig. S8b) and used the MATLAB tool Assisted Free Hand to obtain the nucleus masks for each cell (Fig. S8c) by defining the contour of each nucleus. Second, we applied the masks for all the S294P images (Fig. S8d) and computed the median intensity of the fluorescence intensity for each nucleus. Finally, from the histogram distributions of median intensity we obtained the CDF of ethanol-exposed samples and took the 95% median intensity, which we used to define the inactive/active threshold. A 5% threshold was taken as a reference for activation because 5% of cells were activated by vehicle. Thus, those cells with a median intensity per pixel larger than the threshold are considered to be active. For the analysis, we took 27 cells exposed to ethanol as a negative control, 117 cells exposed to P4 and 116 cells exposed to CRG047.

### Expression analysis

Cells were induced for 30 min as described and after 3 h RNA was isolated using Qiagen RNAeasy kit following manufacturer’s instructions. RNA concentration was measured with a Qubit fluorometer and RNA was subjected to Bioanalyzer for quality control.

For gene expression analysis, RNA (250 ng) was subjected to cDNA synthesis using the qScript cDNA Synthesis kit (Quanta Biosciences). qPCR was carried out using the LightCycler FastStart DNA Master SYBR Green I kit (Roche), and specific primers selected from the list of available designed primers at Primer Bank (https://pga.mgh.harvard.edu/primerbank). [35] As a reference gene, GAPDH was used. Data shown represents the mean of at least three independent experiments. Fold change was calculated using Δ-Δct method. Specific primer sequences are listed in the table below.

**Table:** 

Gene	Forward	Reverse
MMTV-Luc	ATGGAACAACTTTACCGACCGCGC	AGAAGTCGGGGAAGCGGTTGCAAA
EGFR	CAGCCTATGTCCAGGTCGAG	TCGGCCAGTCTGTCTAAAGC
Snail1	TGACATCTGAGTGGGTCTGG	ACCCACACTGGCGAGAAG
Myc	AGAGTCTGGATCACCTTCTG	GGTTGTTGCTGATCTGTCTC
TiPARP	CTTTGGACAGGGAATACGCATGG	AATGGAGGCTGTGAGCAATGTGAG
GAPDH	GAGTCAACGGATTTGGTCGT	TTGATTTTGGAGGGATCTCG

### RNA-seq, pipeline and differential gene expression analysis

RNA-seq preparation and analysis was done as described by Ferrari*.* [36] Libraries were prepared using 1 μg polyA + RNA by PCR amplification of cDNA with bar-coded primers using the Illumina TruSeq kit at the CRG Genomic Facility. Libraries were sequenced using Illumina HIseq-2500 to obtain pair-ended (PE) 100-base-long reads.

Sequencing adapters and low-quality ends were trimmed from the reads with Trimmomatic, using the parameter values recommended by Bolger [37] and elsewhere.

(https://goo.gl/VzoqQq) (trimmomatic PE raw_fastq trimmed_fastq ILLUMINACLIP:TruSeq3-PE.fa:2:30:12:1:true LEADING:3 TRAILING:3 MAXINFO:50:0.999 MINLEN:36). The trimmed reads were aligned to GRCh38 [38] using STAR [39]. First, the genome index files for STAR were generated with: star–runMode genomeGenerate–genomeDir GENOME_DIR–genomeFastaFiles genome_fasta– runThreadN slots–sjdbOverhang read_length–sjdbGTFfile sjdb–outFileNamePrefix GENOME_DIR/ where genomeFasta is the FASTA file containing the GRCh38 sequence downloaded from the University of California Santa Cruz (UCSC) Genome Browser, excluding the random scaffolds and the alternative haplotypes; and sjdb is the GTF file with the GENCODE’s V24 annotation.

Second, trimmed reads were aligned to the indexed genome with: star–genomeDir GENOME_DIR/–genomeLoad NoSharedMemory–runThreadNslots–outFilterType “BySJout”–outFilterMultimapNmax 20–alignSJoverhangMin 8–alignSJDBoverhangMin 1–outFilterMismatchNmax 999–outFilterMismatchNoverLmax 0.04–alignIntronMin 20– alignIntronMax 1000000–alignMatesGapMax 1000000–readFilesInread1 read2– outSAMtype BAM SortedByCoordinate–outTmpDir TMP_DIR/–outFileNamePrefix ODIR1/$sample_id.–outWigType bedGraph–readFilesCommand zcat (https://docs.google.com/document/d/1yRZevDdjxkEmda9WF5- qoaRjIROZmicndPl3xetFftY/edit?usp = sharing).

To avoid mis-alignment, we only accepted uniquely mapped reads and we focused on AE placed within intergenic regions (at least 5 kb away from any human TSS) or on the opposite strand of a known annotated transcript.

Differences in gene expression were calculated using a DESeq2. Genes with fold change (FC) ± 1.5 (*p* value < 0.05; FDR < 0.01) were considered as significantly regulated.

### Cell proliferation

T47D cells were plated for hormone induction in a 96-well plate at 1 × 10^4^/well density and induced with either vehicle, P4 or CRG047 for 30 min, followed by 16 h of culture in hormone-free medium, and measured BrdU incorporation into DNA. ELISA BrdU Colorimetric assay (Roche) was performed according to manufacturer’s instructions. Average results from 12 independent experiments are shown.

## Mass spectrometry

### Chemicals, reagents and lipid standards

Optima® LC/MS grade water, methanol, acetonitrile, 2-propanol and formic acid were from Fisher Scientific (Fair Lawn, NJ, USA). Ammonium formate for mass spectrometry and toluene of analytical standard quality were from Sigma-Aldrich (Sigma Chemical Co., St Louis, MO, USA). Mass spectrometer calibration solutions, Pierce LTQ Velos ESI Positive Ion Calibration Solution and Pierce LTQ Velos ESI Negative Ion Calibration solutions, were provided by Thermo Fisher Scientific (Waltham, MA).

### Sample preparation for analysis

Cells were grown and induced as above. After induction cells were collected and total extract was obtained in lysis buffer. The Bligh and Dyer protocol was applied to extract the lipidic fraction [40]. Dried extracts were reconstituted with 50 µl MeOH:Toluene (9:1,v/v) and vortexed for 30 s before injection. UHPLC-HRMS analysis. CRG047, CRG049, progesterone and promegestone analyses were performed in an ULTIMATE 3000 (Thermo Scientific) UHPLC system coupled to a Qexactive HF-X (Thermo Scientific) quadrupole-orbitrap mass spectrometer. A reverse phase column (Acquity UPLC HSS T3, 100 × 2.1 mm, 1.8 µm) and a precolumn (Acquity UPLC HSS T3 1.8 µm VanGuard™) were used. Mobile phases and UHPLC settings are shown in Table S2.

HRMS was operated using the electrospray source in positive mode. The ionization source parameters are shown Table S2. MS data acquisition was performed in mode t- SIM/dd-MS^2^ with inclusion list containing the parent ion mass of target analytes. Data was processed using FreeStyle™ 1.6 SP1 (Thermo Scientific).

### Identification and quantification

Identity of each compound was confirmed by MS/MS in all samples and quantification was performed by the external standard method, using integration areas from extracted SIM ion chromatograms of the calibration regression of CRG047, CRG049, progesterone and promegestone internal standards. For this purpose, eight solutions containing the internal standards in concentrations ranging from 0.05 to 1000 nM were analyzed in triplicate. Method sensitivity was studied determining calibration curve slopes and detection (LOD) and quantification (LOQ) limit values. The LOD and LOQ for each internal standard were calculated as the lowest concentration that results in a CV ≤ 20%.

### Chromatin immunoprecipitation

ChIP assays were performed as described [41] using Progesterone Receptor antibody [Alpha PR6] (Abcam ab2765).

Quantification of chromatin immunoprecipitation was performed by real time PCR using Roche Lightcycler 2.0, with the specific oligos for the determined regions listed below.

**Table:** 

Gene	Forward	Reverse
MMTV-NucB	GGTTTACATAAGCATTTACATAAGATTTGG	GGTTACAAACTGTTCTTAAAACGAGGATG
EGFR	AAGAAAGTTGGGAGCGGTTC	CGTCCTTTCCTGTTTCCTTG
TiPARP	CGGCTGTGAGGAAGGAAG	TGGGGAGTAGGCAAATAAACA
Fos	CCGGGGATAGCCTCTCTTAC	GTGGGAATGAAGTTGGCACT
ß-actin	GCTGTTCCAGGCTCTGTTCC	GCTCACACGCCACAACATG

The fold enrichment of target sequence in the immunoprecipitated (IP) compared to input (Ref) fractions was calculated using the comparative Ct method (the number of cycles required to reach a threshold concentration) with the Eq. 2^Ct(IP)−Ct(Ref)^. Values were corrected by the human β-actin gene and presented as relative abundance over vehicle.

### Immunoblot

Induced cells were washed twice in cold PBS and collected in lysis buffer (25 mM Tris pH 7.5, 1% SDS, 1 mM EDTA, 1 mM EGTA and protease inhibitors). Protein extracts were quantified and equal amounts were loaded in an 8% SDS-PAGE, transferred into Nitrocellulose membrane, blotted against indicated primary antibodies and HRP-conjugated correspondent secondary antibodies. Primary antibodies: Total Progesterone Receptor antibody [Alpha PR6] (Abcam ab2765), Progesterone Receptor Ser294Phospho, (Abcam ab61785), Phospho-p44/42 MAPK (Erk1/2) (Thr202/Tyr204) (Cell Signaling #9106S), HP1 gamma (Millipore 05–690).

### Supplementary Information

Below is the link to the electronic supplementary material.

Supplementary file1 Experimental procedures and spectroscopic data for all new compounds (1H NMR, 13C NMR, 31P NMR), including images of NMR spectra (DOCX 25099 KB)

Supplementary file2 (AVI 9631 KB)

## Data Availability

Sequencing data have been deposited in GEO under accession number GSE226789. Datasets Generated: Reporting Standards: N/A.

## Declarations

### Research involving human and animal particpants

No human subjects have been used.
