# Supplementary material for: A progesterone derivative linked to a stable phospholipid activates breast cancer cell response without leaving the cell membrane
Source: Cell Mol Life Sci. 2024 Feb 22;81(1):98. doi: 10.1007/s00018-024-05116-3 (PMC10884080; doi:10.1007/s00018-024-05116-3)
Supplement: Supplementary file 1 — Supplementary file1 Experimental procedures and spectroscopic data for all new compounds (1H NMR, 13C NMR, 31P NMR), including images of NMR spectra (DOCX 25099 KB) [file 18_2024_5116_MOESM1_ESM.docx]

# Font-Mateu et al. SUPPLEMENTARY INFORMATION

**Table S1. Spectroscopic properties of probes CRG033, CRG034, CRG047 and CRG048 in EtOH.**

| **Compound** | **λex/λem (nm)** | **ε (M^-1^·cm^-1^)** | **ΦF** |
| --- | --- | --- | --- |
| **CRG033** | 550/625 | 3.64 x 10^4^ | 0.33 |
| **CRG034** | 550/625 | 4.17 x 10^4^ | 0.39 |
| **CRG047** | 550/625 | 4.22 x 10^4^ | 0.31 |
| **CRG048** | 550/625 | 4.24 x 10^4^ | 0.31 |

**Table S2. Ultra-High Performance Liquid Chromatography (UHPLC) and Mass Spectrometry (MS) analysis settings.**

| **UHPLC** | **MS** |  |
| --- | --- | --- |
|  | **ESI probe** | **Q exactive HF-X** |
| ULTIMATE 3000 UHPLC (Thermo  Scientific) | **Sheath Gas:**  35 | **List of target compounds:**  Progesterone [M+H]+ 315.2319  **CRG047** [M+2H/2]2+ 822.0225  **CRG049** [M+H]+ 317.2117  Promegestone (**R5020**) [M+H]+  326.5 |
| **Pre-column:** Acquity UPLC HSS T3 1.8 µm (Waters) | **Aux Gas:** 10 | **tSIM Settings**  R=60 K, FWHM at m/z 200  AGC target: 1e5 Maximum IT 110ms Loop count: 1  MSX count: 3  Isolation window: 4.0 m/z Isolation offset: 1.0 m/z |
| **Column:** Acquity UPLC HSS T3, 100x2.1 mm, 1.8 µm (Waters) | **Sweep gas flow rate:** 0 | **ddMS2 Settings**  R=30 K, FWHM at m/z 200  AGC target 2e5 Maximum IT: 50 ms Loop count: 1  MSX count:3  Isolation window: 0.4 m/z  HCD NCE: 27 |
| **Column temperature:** 65 ºC | **Spray Voltage:**  3.20 kV | **dd Settings**  Minimum AGC target: 8.00e3 Dynamic exclusion: 2s |
| **Flow rate:** 300 µL/min | **Cap Temp.:**  285 ºC |  |
| **Mobile phase A:** H2O with 10 mM ammonium formate and 0.1% formic  acid | **Heater Temp.:** 370  ºC |  |
| **Mobile phase B:** acetonitrile/Isopropanol (10:90, v/v) with 10 mM ammonium formate and  0.1% formic acid |  |  |
| **Gradient:** 0-1min, 50%B isocratic; 1-5 min, from 50 to 100% B; 5-6 min, 100  % B Isocratic; and re-equilibration of  the column to initial conditions. |  |  |
| **Automatic injector temperature:** 4ºC  **Injection volume:** 10 µL |  |  |

**SUPPLEMENTARY FIGURE LEGENDS**

**Figure S1.** Excitation (dashed line) and emission (continuous line) spectra of compounds CRG033 (a), CRG047 (b), CRG034 (c) and CRG048 (d) in EtOH. λex and λem were respectively 550 and 625 nm for all compounds.

**Figure S2.** a) Microscale thermophoresis (MST). Initial fluorescence representation of CRG033 interaction with full-length PR. b-d). MST dose/response representation of 2 readings of the same experiment (average ± SD is shown) of the indicated compounds. Normalized fluorescence plotted against increasing concentrations of PRb. 2 μM concentrations of the compounds were assessed with increasing concentrations of PRb.

**Figure S3.** TIRF imaging of single molecules diffusing on the cell membrane of breast cancer cells exposed to 10 nM of the progesterone derivative CRG047 dissolved in PBS. Single molecules are detected with a custom software and projected into 1 single frame to build up cartography maps as shown in Fig. 4a. (Note to editor: First frame shown, video attached as Supporting Information (SI) - "Supplementary Fig. S3".

**Figure S4.** a) Hierarchical clustering of the gene expression signatures of R5020 regulated genes following inhibition of the protein indicated (PKA, JAK, CDK, MSK, Akt, ER or SRC) with the gene expression signature following CRG047 treatment. Analysis was carried out using Morpheus (Broad Institute); K means clustering on rows indicates 6 significantly distinct gene groups. Each row indicates a specific gene and the data was log2 normalized per row before clustering. b) Correlation matrix between significant genes from both treatments, clustered by genes down-regulated (red) and up-regulated (blue). Boxplots depict correlation of genes enclosed in each category, showing most of the genes although having a low FC over vehicle they fall on the same side of activation (positive or negative). c) Similarity matrix comparing the gene expression signatures of R5020 regulated genes following inhibition of the protein indicated (PKA, JAK, CDK, MSK, Akt, ER or SRC) with the gene expression signature following CRG047 treatment. Analysis was carried out using Morpheus (Broad Institute); negative Pearson correlations are shown in blue and positive in red (-1 to 1).

**Figure S5.** Mass spectrometry data on analyzed compounds. UHPLC-ESI-MS/MS SIM traces of precursor ions of CRG047 (a-d), P4 (e-h), R5020 (i-l) and CRG049 (m-p) compound for exposure to 10 nM and 100 nM as indicated in nuclear extracts (NE) and total extracts (TE). Panels q-t show the signals arising from internal standards (IS) of CRG047, P4, R5020 and CRG049 at indicated concentrations respectively.

**Figure S6.** a) Table represents pmol/106 cells of the indicated compounds detected (CRG047 or CRG049) following addition of CRG047 at the concentrations indicated. b-c) Calibration curves of indicated compounds for mass spectrometry analysis derived from internal standards for CRG047 and CRG049 as indicated.

**Figure S7.** a) PR-negative breast cancer cell line T47D-Y stably expressing a single copy of the MMTV promoter was transfected with plasmids for the expression of either wild type PRb, palmitoylation mutant PRbC820A or with empty plasmids and incubated with either vehicle, progesterone (P4) or CRG047 as indicated. RNA signal for MMTV is shown. Average ± SD of 3 independent experiments is shown. Fold changes are relative to the average response of vehicle-incubated cells. *: p-value < 0.05; **: p-value < 0.01; ns: p-value > 0.05. Statistical analysis was done by 1 tail T-test comparing the indicated conditions. b) U2OS cells were co-transfected with plasmids expressing a MMTV-luc reporter and either wild type PRb or palmitoylation mutant PRbC820A and incubated with either vehicle, progesterone (P4) or CRG047 and qPCR was done against MMTV. Histograms show average ± SD of 2 independent experiments.

**Figure S8.** Procedure for the quantification of immunofluorescence. a) PR Ser 294 phosphorylation (S294P) immunofluorescent image. b) DAPI image. c) Masks for each nucleus obtained from the DAPI image (b). d) The result of applying the mask (c) to the S294P image (a). Images (a-d) have been normalized to the maximum intensity value for better visualization. Scale bar for all panels: 10 μm.

**Figure S9.** ^1^H (a) and ^13^C (b) NMR spectra of compound **CRG002** in CDCl3. **Figure S10.** ^1^H (a) and ^13^C (b) NMR spectra of compound **CRG041** in CDCl3. **Figure S11.** ^1^H (a) and ^13^C (b) NMR spectra of compound **CRG003** in CDCl3.

**Figure S12.** ^1^H (a) and ^13^C (b) NMR spectra of compound **CRG042** in CDCl3. **Figure S13.** ^1^H (a) and ^13^C (b) NMR spectra of compound **CRG011** in CDCl3. **Figure S14.** ^1^H (a) and ^13^C (b) NMR spectra of compound **CRG036** in CDCl3. **Figure S15.** ^1^H (a) and ^13^C (b) NMR spectra of compound **CRG012** in CDCl3. **Figure S16.** ^1^H (a) and ^13^C (b) NMR spectra of compound **CRG037** in CDCl3. **Figure S17.** ^1^H (a) and ^13^C (b) NMR spectra of compound **CRG005** in CDCl3. **Figure S18.** ^1^H (a) and ^13^C (b) NMR spectra of compound **CRG006** in CDCl3. **Figure S19.** ^1^H (a) and ^13^C (b) NMR spectra of compound **CRG013** in CDCl3. **Figure S20.** ^1^H (a) and ^13^C (b) NMR spectra of compound **CRG007** in CDCl3. **Figure S21.** ^1^H (a) and ^13^C (b) NMR spectra of compound **CRG043** in CDCl3. **Figure S22.** ^1^H (a) and ^13^C (b) NMR spectra of compound **CRG008** in CDCl3. **Figure S23.** ^1^H (a) and ^13^C (b) NMR spectra of compound **CRG044** in CDCl3. **Figure S24.** ^1^H (a) and ^13^C (b) NMR spectra of compound **CRG009** in CDCl3. **Figure S25.** ^1^H (a) and ^13^C (b) NMR spectra of compound **CRG045** in CDCl3. **Figure S26.** ^1^H (a) and ^13^C (b) NMR spectra of compound **CRG014** in CDCl3. **Figure S27.** ^1^H (a) and ^13^C (b) NMR spectra of compound **CRG038** in CDCl3. **Figure S28.** ^1^H (a) and ^13^C (b) NMR spectra of compound **CRG015** in CDCl3. **Figure S29.** ^1^H (a) and ^13^C (b) NMR spectra of compound **CRG039** in CDCl3. **Figure S30.** ^1^H (a) and ^13^C (b) NMR spectra of compound **CRG016** in CDCl3. **Figure S31.** ^1^H (a) and ^13^C (b) NMR spectra of compound **CRG046** in CDCl3. **Figure S32.** ^1^H (a) and ^13^C (b) NMR spectra of compound **CRG049** in CDCl3.

**Figure S33.** ^1^H (a) and ^13^C (b) NMR spectra of compound **CRG023** in CDCl3. **Figure S34.** ^1^H (a) and ^13^C (b) NMR spectra of compound **CRG024** in CDCl3. **Figure S35.** ^1^H (a) and ^13^C (b) NMR spectra of compound **CRG029** in CDCl3. **Figure S36.** ^1^H (a) and ^13^C (b) NMR spectra of compound **CRG031** in CDCl3.

**Figure S37.** ^1^H (a), ^13^C (b) and ^31^P (c) NMR spectra of compound **CRG032** in CD3OD/CD3CN (1:1).

**Figure S38.** ^1^H (a), ^13^C (b) and ^31^P (c) NMR spectra of compound **CRG033** in CD3OD/CD3CN (1:1).

**Figure S39.** ^1^H (a), ^13^C (b) and ^31^P (c) NMR spectra of compound **CRG047** in CD3OD. **Figure S40.** ^1^H (a), ^13^C (b) and ^31^P (c) NMR spectra of compound **CRG034** in CD3OD. **Figure S41.** ^1^H (a), ^13^C (b) and ^31^P (c) NMR spectra of compound **CRG048** in CD3OD.

**ORGANIC SYNTHESIS**

# Synthetic route for probes CRG033, CRG047, CRG034 and CRG048.

# Note: NMR spectra of the compounds mentioned below are shown in the supplementary figures S9 to S41.

- 1. Synthesis of PEG-linkers CRG003, CRG042, CRG012 and CRG037.

The synthesis of the heterobifunctional poly(ethylene glycol) (PEG) linkers CRG003, CRG042, CRG012 and CRG037 is depicted in Scheme S1. For the synthesis of amino-PEG-azides CRG003 and CRG042, both terminal hydroxyl groups of tetra or octaethylene glycol (compounds 1 and 2) were mesylated yielding the corresponding dimesyl intermediates which were subsequently transformed to the di-azido PEGs CRG002 and CRG041. Selective transformation of one of the azido groups to an amine *via* Staundinger reduction gave the desired heterobifunctional linkers CRG003 and CRG042.

On the other hand, desymmetrization of 1 and 2 by conversion of one of their hydroxyl groups to an azido functionality allowed the obtention of monoazide products CRG011 and CRG036 in moderate yields. Finally, treatment of the resulting alcohols with MsCl and TsCl, yielded the sulfonate esters CRG012 and CRG037, respectively.


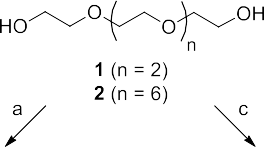

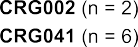

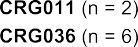

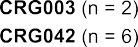

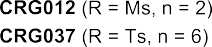


**Scheme S1.** Synthesis of PEG-linkers CRG003, CRG042, CRG012 and CRG037. Reagents and conditions: (**a**) (i) MsCl, TEA, CH2Cl2, 0 °C to rt. (ii) NaN3, DMF, 65 °C, 97% for CRG002, quant. yield for CRG041; (**b**) PPh3, 0.5 M HCl /Et2O (1:1), rt, 75% for CRG003, 72% for CRG042; (**c**) (i) MsCl, TEA, CH2Cl2, 0 °C to rt. (ii) NaN3, DMF, 65 °C, 36% for CRG011, 41% for CRG036; (**d**) For CRG012: MsCl, TEA, CH2Cl2, 0 °C to rt, 94%. For CRG037: TsCl, KOH, CH2Cl2, 0 °C, 93%.

- 1. Synthesis of steroidal moieties CRG006 and CRG013.

With the required heterobifunctional linkers in hand, the differently functionalized steroidal scaffolds CRG006 and CRG013 were also prepared (Scheme S2). First, protection of the 3β- hydroxyl group of pregnenolone (**3**) as a *tert*-butyldimethylsilyl ether afforded compound CRG005, which was treated with sodium hypobromide in dioxane/water to give the corresponding 17β-carboxylic acid CRG006. Subsequent reduction of CRG006 using lithium aluminium hydride provided alcohol CRG013 in 61% yield over three steps.


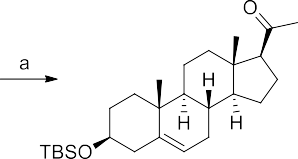


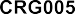


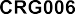


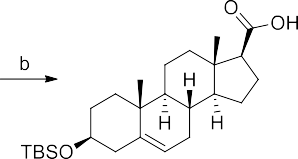

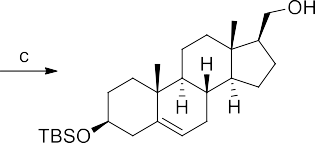

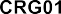


**Scheme S2.** Synthesis of steroidal moieties CRG006 and CRG013. Reagents and conditions: (**a**) TBSCl, imidazole, DMF, 0 °C to rt, 94%; (**b**) NaOBr, H2O/dioxane, 0 °C to rt, 83%; (c) LiAlH4, THF, 0 °C to rt, 78%.

- 1. Synthesis of azides CRG009, CRG045, CRG016 and CRG046.

In order to obtain the series of probes with an amide bond between the steroidal moiety and the azido-terminated linker, amines CRG003 and CRG042 were acylated with carboxylic acid CRG006, yielding amides CRG007 and CRG043, respectively (Scheme S3). Alternatively, treatment of alcohol CRG013 with sulfonate esters CRG012 and CRG037 under basic conditions gave rise to the ether-linked compounds CRG014 and CRG038, respectively.

Although tosylate CRG037 appeared to be more reactive towards the alcoxide generated from CRG013 than its mesylate counterpart (compound CRG012), both reactions occurred in low to moderate yields. Apart from the low reactivity of the starting materials, the observed decrease in yield could also be attributed to the fact the TBS group presented certain lability under the reaction conditions, leading to the formation of considerable amounts of deprotected CRG013 during the course of the reaction (as seen by 1H/13C-NMR characterization of the crude reaction mixture).

Once both amide and ether-linked derivatives were obtained, the synthetic route continued with the TBAF-mediated removal of the TBS protecting group, which took place efficiently, affording the 3β-alcohols CRG008, CRG044, CRG015 and CRG039. Finally, Oppenauer oxidation of the hydroxy group at C3 (which occurred with concomitant isomerisation of the double bond) afforded the α,β-unsaturated ketones CRG009, CRG045, CRG016 and CRG046, respectively.


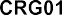


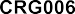

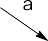

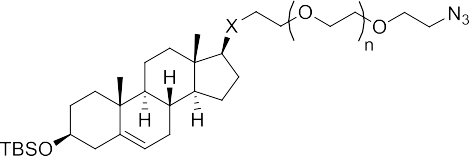

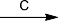


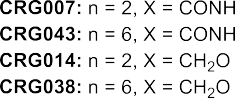

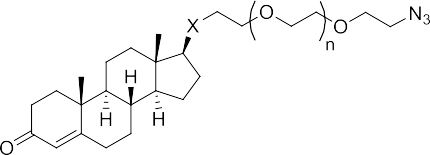

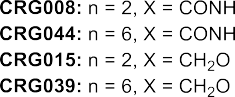

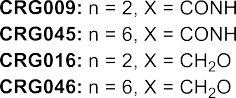


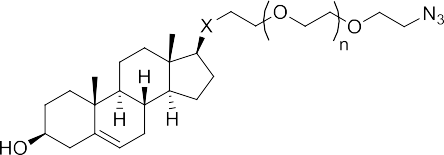

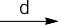


**Scheme S3.** Synthesis of azides CRG009, CRG045, CRG016 and CRG046. Reagents and conditions: (**a**) Selected amine, EDC, HOBt, TEA, CH2Cl2, 0 °C to rt, 80% for CRG007, 92% for CRG043; (**b**) Selected sulfonate ester, NaH, THF, 65 °C, 24% for CRG014, 53% for CRG038; (**c**) TBAF, THF, reflux, 76-97%; (d) Al(O*^t^*Bu)3, acetone/toluene, reflux, 58-92%.

- 1. Synthesis of probes CRG033, CRG047, CRG034 and CRG048.

As depicted in Scheme S4, the synthesis of the alkyne-terminated phosphatidylcholine derivative CRG032 started with (*S*)-(-)-glycidol (**4**), which served as the source of chirality. After protection of **4** as the trityl ether CRG023, the epoxide was regioselectively opened with the *in situ* generated alcoxyde of hexadecanol, yielding alcohol CRG024 with retention of the configuration at the C2 position.

Treatment of CRG024 with an excess of 1,8-dibromooctane under basic conditions afforded an inseparable and enriched crude mixture of the bromo derivative 5, which was directly treated with the phenoxyde form of 2-hydroxy Nile Red,1 allowing the nucleophilic displacement of the remaining bromo group at the distal position of the side chain of the lipid structure and yielding compound CRG029 in 54% yield over two steps.

Acid-mediated removal of the trityl group gave compound CRG031 which was transformed to the zwitterionic aminophosphate CRG032 in a two-step sequence consisting of a phosphorylation of the primary hydroxyl at C1 followed by a nucleophilic opening of the resulting cyclic phosphate by 3-dimethylamino-1-propyne.2,3

The desired probes were finally assembled by means of a Cu(I)-catalyzed azide-alkyne [3 + 2] dipolar cycloaddition (CuAAC) between the terminal alkyne in CRG032 and the azido group present in fragments CRG009, CRG045, CRG016 and CRG046, giving probes CRG033, CRG047, CRG034 and CRG048, respectively.


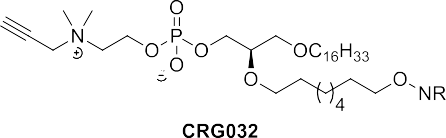

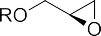

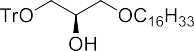

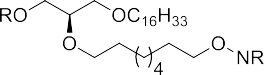

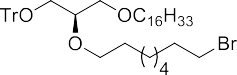

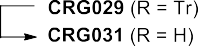


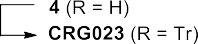

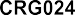


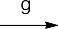

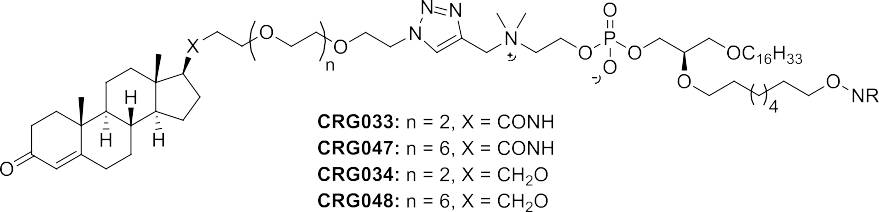


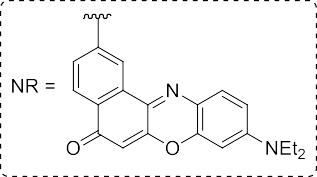


**Scheme S4.** Synthesis of probes CRG033, CRG047, CRG034 and CRG048. Reagents and conditions: (**a**) TrCl, TEA, CH2Cl2, 0 °C to rt, 92%; (**b**) Hexadecanol, KO*^t^*Bu, DMF, 80 °C, 55%; (**c**) 1,8-dibromooctane, NaH, DMF, 65 °C; (**d**) 9-(diethylamino)-2-hydroxy-5*H*-benzo[*a*]phenoxazin- 5-one (2-hydroxy Nile Red), K2CO3, DMF, 65 °C, 54% over two steps; (**e**) *p*-TsOH, MeOH/CHCl3, 0 °C to rt, 91%; (**f**) (I) 2-chloro-1,3,2-dioxaphospholane 2-oxide, TEA, DMAP, toluene, 0 °C to rt

(II) 3-dimethylamino-1-propyne, MeCN, 80 °C, 42% over two steps; (**g**) Selected azide, CuI, DIPEA, H2O/MeCN (1:1), rt, 51-95%.

- 1. Synthesis of carboxylic acid CRG049.

Compound CRG049 was obtained (Scheme S5) by oxidation of the commercially available progesterone (**6**) following an adapted version of the synthetic protocol described by Lao *et al*.4


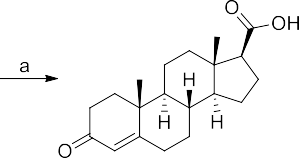

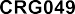


**Scheme S5.** Synthesis of carboxylic acid CRG049. Reagents and conditions: (**a**) NaOBr, H2O/dioxane, 0 °C to rt, 74%.

# Synthetic experimental part (Synthetic methods).

- 1. General remarks.

Unless otherwise stated, reactions were carried out under nitrogen atmosphere. Dry solvents were obtained by passing through an activated alumina column on a Solvent Purification System (SPS). Acetone was dried over anhydrous CaCl2 and distilled prior to use. Commercially available reagents and solvents were used with no further purification. All reactions were monitored by TLC analysis using ALUGRAM® SIL G/UV254 precoated aluminum sheets (Machery-Nagel). UV light was used as the visualizing agent and a 5% (w/v) ethanolic solution of phosphomolybdic acid as the developing agent. Flash column chromatography was carried out with the indicated solvents using flash-grade silica gel (37-70 μm). Yields refer to chromatographically and spectroscopically pure compounds, unless otherwise stated.

NMR spectra were recorded at room temperature on a Varian Mercury 400 instrument. The chemical shifts (δ) are reported in ppm relative to the solvent signal, and coupling constants (*J*) are reported in Hertz (Hz). ^31^P chemical shifts are relative to an 85 % H3PO4 external reference (0 ppm). In the case of NMR spectra recorded in CD3OD/CD3CN mixtures, chemical shifts are expressed relative to the residual peak of CD3OD. The following abbreviations are used to define the multiplicities in 1H NMR spectra: s = singlet, d = doublet, t = triplet, q = quartet, dd = doublet of doublets, ddd = doublet of doublet of doublets, dt = doublet of triplets, td = triplet of doublets, m = multiplet, br = broad signal and app = apparent. High Resolution Mass Spectrometry analyses were carried out on an Acquity UPLC system coupled to a LCT Premier orthogonal accelerated time-of-flight mass spectrometer (Waters) using the electrospray ionization (ESI) technique. Optical rotations were measured at room temperature on a Perkin Elmer 341 polarimeter.

- 1. General synthetic methods.

# General procedure 1: Preparation of diazido derivatives from polyethylene glycols.

MsCl (3.0 eq.) was added dropwise to a mixture of TEA (3.0 eq.) and the corresponding diol (15 mmol) in CH2Cl2 (100 mL) stirring at 0 °C. After the addition was complete, the reaction was allowed to proceed at 0 °C for 2 h and then at rt for a further 2 h. The reaction mixture was then poured into 0.5 M HCl and the mixture was extracted with CH2Cl2. The combined organic layers were washed with brine, dried over anhydrous MgSO4, filtered and evaporated to dryness. The resulting residue was dissolved in DMF (60 mL) and the mixture was treated with NaN3 (5.0 eq.) and stirred overnight at 65 °C. DMF was removed *in vacuo* and the residue was resuspended in Et2O, filtered over celite, and washed with Et2O. Finally, the combined filtrates were concentrated *in vacuo* to give the corresponding diazido derivatives, which were used without further purification.

# General procedure 2: Monoreduction of diazido derivatives.

To an ice-cooled solution of the corresponding diazido derivative (15 mmol) in 0.5 M HCl (60 mL) was added dropwise a solution of triphenyl phosphine (0.9 eq.) in Et2O (60 mL). After stirring overnight at rt, the reaction mixture was washed with EtOAc in order to remove the unreacted starting materials and triphenylphosphine oxide that was formed during the

reaction. The aqueous layer was collected, cooled to 0 °C, and treated with 1M aq. KOH until the pH of the solution was around 12. This solution was finally extracted with CH2Cl2 and the combined organic layers were dried over anhydrous MgSO4, filtered and evaporated to dryness to give the required mono amines, which were used without further purification.

# General procedure 3: Preparation of monoazido derivatives from polyethylene glycols.

MsCl (0.9 eq.) was added dropwise to a mixture of TEA (1.5 eq.) and the corresponding diol (14 mmol) in CH2Cl2 (55 mL) stirring at 0 °C. After the addition was complete, the reaction was allowed to proceed at 0 °C for 1 h and then at rt for a further 2 h. The reaction mixture was then poured into 0.5 M HCl and the mixture was extracted with CH2Cl2. The combined organic layers were washed with brine, dried over anhydrous MgSO4, filtered and evaporated to dryness to give a mixture of mono- and di-mesylated derivatives. The resulting residue was dissolved in DMF (35 mL) and the mixture was treated with NaN3 (5.0 eq.) and stirred over night at 65 °C. DMF was removed *in vacuo* and the residue was resuspended in Et2O, filtered over celite, and washed with Et2O. Finally, the combined filtrates were concentrated under reduced pressure to give a crude mixture, which was purified as indicated for each compound.

# General procedure 4: EDC/HOBt-mediated coupling for amide-bond formation.

HOBt (1.2 eq.) and EDC (1.2 eq.) were added portionwise to a solution of **CRG006** (0.75 mmol) in CH2Cl2 (15 mL) at 0 °C. The resulting solution was stirred at the same temperature for 15 min and was added dropwise to an ice-cooled solution of the selected amine (1.1 eq.) and TEA (1.5 eq.) in CH2Cl2 (10 mL). After stirring at rt for 2h, the reaction mixture was diluted with water and was extracted with CH2Cl2. The combined organic layers were washed with brine, dried over anhydrous MgSO4, filtered and evaporated *in vacuo* to give a residue, which was purified as indicated for each compound.

# General procedure 5: fluoride-mediated deprotection of TBS groups.

To a solution of the corresponding TBS-protected alcohol (0.5 mmol) in THF (8.5 mL) was added dropwise TBAF (1 M in THF, 2 eq.) at 0 °C. The reaction mixture was refluxed for 4h, then cooled down to rt and quenched with saturated aq. NH4Cl (10 mL). The resulting mixture was extracted with Et2O and the combined organic layers were washed with brine, dried over anhydrous MgSO4, filtered, and evaporated to give the crude products. Purification by flash chromatography on silica gel using the indicated conditions afforded the required alcohols.

# General procedure 6: Oppenauer oxidation of allylic alcohols.

Aluminum *tert*-butoxide (1.5 eq.) was added in one portion to a solution of the corresponding allylic alcohol (0.5 mmol) in acetone (3 mL) and toluene (7 mL). The reaction mixture was refluxed for 7 h and cooled to room temperature. H2O was then added and the mixture was extracted with Et2O. The organic layers were combined, washed with brine, dried over anhydrous MgSO4, filtered and evaporated to give a crude mixture, which was purified as indicated for each compound.

# General procedure 7: Williamson ether synthesis.

To a solution of alcohol CRG013 (0.3 mmol) in THF (2 mL) was added portionwise NaH (60 wt.% in mineral oil, 2.0 eq.) at 0 °C and the mixture was stirred at the same temperature for 10 min. A solution of the corresponding sulfonate ester (1.0 eq.) in THF (1 mL) was then added dropwise and the resulting mixture was stirred at 65 °C overnight. After cooling down to rt, the reaction mixture was carefully diluted with water and extracted with Et2O. The combined organic layers were washed with brine, dried over anhydrous MgSO4, filtered and evaporated to give a residue, which was purified as indicated for each compound.

# General procedure 8: CuAAC between CRG032 and selected azides.

To a solution of CRG032 (0.025 mmol) and the selected azide (1.15 eq.) in H2O/MeCN (1:1) (2 mL), DIPEA (2 eq.) and CuI (0.15 eq.) were added. After stirring at rt for 1 h, the reaction mixture was diluted with water (5 mL) and was extracted with CHCl3. The combined organic layers were washed with brine, dried over anhydrous MgSO4 and concentrated to give a residue which was purified as indicated for each compound.

- 1. Synthesis and characterization of compounds.

# 1-azido-2-(2-(2-(2-azidoethoxy)ethoxy)ethoxy)ethane (CRG002).

Compound **CRG002** (colourless oil, 3.66 g, 97%) was obtained from tetraethylene glycol (3.00 g, 15.45 mmol), MsCl (3.59 mL, 46.34 mmol), TEA (6.46 mL, 46.34 mmol) and NaN3 (5.02 g,

77.20 mmol), according to general procedure 1.

^1^H NMR (400 MHz, CDCl3) δ 3.71 – 3.64 (m, 12H), 3.39 (t, *J* = 5.1 Hz, 4H). ^13^C NMR (101 MHz, CDCl3) δ 70.8, 70.8, 70.2, 50.8. HRMS calcd. for C8H16N6O3Na ([M + Na]^+^): 267.1182, found:

267.1176.

# 1,23-diazido-3,6,9,12,15,18,21-heptaoxatricosane (CRG041).


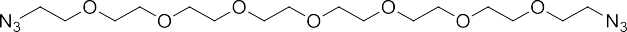


Compound **CRG041** (colourless oil, 205 mg, quant.) was obtained from octaethylene glycol (180 mg, 0.486 mmol), MsCl (113 µL, 1.46 mmol), TEA (203 µL, 1.46 mmol) and NaN3 (158 mg, 0.486 mmol), according to general procedure 1.

^1^H NMR (400 MHz, CDCl3) δ 3.69 – 3.62 (m, 28H), 3.38 (t, *J* = 5.1 Hz, 4H). ^13^C NMR (101 MHz, CDCl3) δ 70.7, 70.7, 70.6, 70.6, 70.0, 50.7. HRMS calcd. for C16H32N6O7Na ([M + Na]^+^): 443.2230,

found: 443.2236.

# 2-(2-(2-(2-azidoethoxy)ethoxy)ethoxy)ethanamine (CRG003).

Compound **CRG003** (pale yellow oil, 2.44 g, 75%) was obtained from **CRG002** (3.65 g, 14.94 mmol) and PPh3 (3.53 g, 13.45 mmol), according to general procedure 2.

^1^H NMR (400 MHz, CDCl3) δ 3.70 – 3.61 (m, 10H), 3.51 (t, *J* = 5.2 Hz, 2H), 3.39 (t, *J* = 4.0 Hz, 2H),

2.86 (t, *J* = 5.2 Hz, 2H). ^13^C NMR (101 MHz, CDCl3) δ 73.5, 70.8, 70.7, 70.7, 70.4, 70.1, 50.8, 41.8. HRMS calcd. for C8H19N4O3 ([M + H]^+^): 219.1457, found: 219.1441.

# 23-azido-3,6,9,12,15,18,21-heptaoxatricosan-1-amine (CRG042).


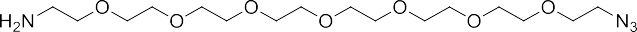


Compound **CRG042** (colourless oil, 168 mg, 72%) was obtained from **CRG041** (250 mg, 0.595 mmol) and PPh3 (141 mg, 0.535 mmol), according to general procedure 2.

^1^H NMR (400 MHz, CDCl3) δ 3.85 – 3.57 (m, 26H), 3.50 (t, *J* = 5.2 Hz, 2H), 3.37 (t, *J* = 4.0 Hz, 2H),

2.85 (t, *J* = 5.0 Hz, 2H). ^13^C NMR (101 MHz, CDCl3) δ 73.3, 70.7, 70.7, 70.6, 70.6, 70.6, 70.3, 70.0,

50.7, 41.8. HRMS calcd. for C16H35N4O7 ([M + H]^+^): 395.2506, found: 395.2492.

# 2-(2-(2-(2-azidoethoxy)ethoxy)ethoxy)etanol (CRG011).

Compound **CRG011** (colourless oil, 1.09 g, 36%) was obtained from tetraethylene glycol (2.7 g, 13.90 mmol), MsCl (968 µL, 12.51 mmol), TEA (2.91 mL, 20.85 mmol) and NaN3 (4.52 g, 69.50

mmol), according to general procedure 3. The title compound was purified by flash chromatography on silica gel (from 0 to 2.5% MeOH in CH2Cl2).

^1^H NMR (400 MHz, CDCl3) δ 3.75 – 3.71 (m, 2H), 3.71 – 3.64 (m, 10H), 3.64 – 3.59 (m, 2H), 3.40

(t, *J* = 5.1 Hz, 2H), 2.13 (br s, 1H). ^13^C NMR (101 MHz, CDCl3) δ 72.6, 70.8, 70.7, 70.7, 70.4, 70.1,

61.8, 50.7. HRMS calcd. for C8H17N3O4Na ([M + Na]^+^): 242.1117, found: 242.1128.

# 23-azido-3,6,9,12,15,18,21-heptaoxatricosan-1-ol (CRG036).


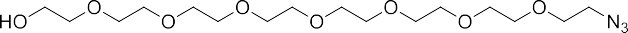


Compound **CRG036** (colourless oil, 110 mg, 41%) was obtained from octaethylene glycol (250 mg, 0.675 mmol), MsCl (47 µL, 0.607 mmol), TEA (141 µL, 1.01 mmol) and NaN3 (220 mg, 3.38 mmol), according to general procedure 3. The title compound was purified by flash chromatography on silica gel (from 0 to 6% MeOH in CH2Cl2).

^1^H NMR (400 MHz, CDCl3) δ 3.73 – 3.68 (m, 2H), 3.68 – 3.61 (m, 26H), 3.61 – 3.56 (m, 2H), 3.38

(t, *J* = 4.0 Hz, 2H). ^13^C NMR (101 MHz, CDCl3) δ 72.7, 70.7, 70.7, 70.7, 70.6, 70.6, 70.6, 70.6, 70.5,

70.3, 70.1, 61.7, 50.7. HRMS calcd. for C16H34N3O8 ([M + H]^+^): 396.2346, found: 396.2319.

# 2-(2-(2-(2-azidoethoxy)ethoxy)ethoxy)ethyl methanesulfonate (CRG012).

TEA (1.06 mL, 7.64 mmol) and MsCl (443 µL, 5.73 mmol) were added dropwise to a solution of **CRG011** (837 mg, 3.82 mmol) in CH2Cl2 (35 mL) at 0 °C. After stirring at rt for 3.5 h, the reaction mixture was poured into 0.5 M HCl and the mixture was extracted with CH2Cl2. The combined organic layers were washed with brine, dried over anhydrous MgSO4, filtered and evaporated to dryness. Purification of the residue by flash chromatography (from 0 to 70% EtOAc in hexane) gave **CRG012** (1.07 g, 94%) as a colourless oil.

^1^H NMR (400 MHz, CDCl3) δ 4.42 – 4.34 (m, 2H), 3.80 – 3.74 (m, 2H), 3.71 – 3.62 (m, 10H), 3.39

(t, *J* = 4.0 Hz, 2H), 3.07 (s, 3H). ^13^C NMR (101 MHz, CDCl3) δ 70.8, 70.7, 70.7, 70.1, 69.4, 69.1,

50.8, 37.8. HRMS calcd. for C9H19N3O6NaS ([M + Na]^+^): 320.0892, found: 320.0888.

# 23-azido-3,6,9,12,15,18,21-heptaoxatricosyl 4-methylbenzenesulfonate (CRG037).


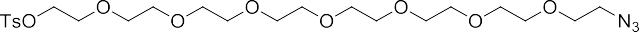


To an ice-cooled solution of **CRG036** (100 mg, 0.253 mmol) in CH2Cl2 (5 mL) was added KOH (57 mg, 1.01 mmol) followed by TsCl (72 mg, 0.379 mmol) and the reaction mixture was stirred at 0 °C for 4 h. A mixture of ice and water was then added and the mixture was extracted with CH2Cl2. The combined organic layers were dried over MgSO4, filtered and concentrated under reduced pressure. Flash chromatography of the residue (from 0 to 3.5% MeOH in CH2Cl2) gave **CRG037** (129 mg, 93%) as a colorless oil.

^1^H NMR (400 MHz, CDCl3) δ 7.80 (d, *J* = 8.3 Hz, 2H), 7.34 (d, *J* = 8.5 Hz, 2H), 4.19 – 4.12 (m, 2H),

3.71 – 3.54 (m, 28H), 3.39 (t, *J* = 4.0 Hz, 2H), 2.45 (s, 3H). ^13^C NMR (101 MHz, CDCl3) δ 144.9,

133.1, 129.9, 128.1, 70.9, 70.8, 70.8, 70.7, 70.7, 70.7, 70.6, 70.1, 69.4, 68.8, 50.8, 21.8. HRMS calcd. for C23H40N3O10S ([M + H]^+^): 550.2434, found: 550.2464.

# 3β-(*tert*-Butyldimethylsilyloxy)-pregn-5-en-20-one (CRG005).

To a stirred solution of pregnenolone (**3**) (3.00 g, 9.48 mmol) in DMF (30 mL) was added imidazole (2.00 g, 29.39 mmol) and TBSCl (2.21 g, 14.69 mmol) at 0 °C. After stirring at rt for 16 h, the reaction mixture was diluted with water and extracted with Et2O. The combined organic layers were washed with brine, dried over anhydrous MgSO4, filtered and evaporated to dryness. The crude residue was purified by flash chromatography (from 0 to 5% EtOAc in hexane) to afford **CRG005** (3.82 g, 94%) as a white solid.

[α]^20^ = +21.0 (*c* 1.0, CHCl3). ^1^H NMR (400 MHz, CDCl3) δ 5.32 (dd, *J* = 3.0, 2.3 Hz, 1H), 3.48 (ddd,

D

*J* = 15.7, 10.9, 4.7 Hz, 1H), 2.53 (t, *J* = 9.0 Hz, 1H), 2.12 (s, 3H), 2.32 – 0.92 (m, 19H), 1.00 (s, 3H),

0.89 (s, 9H), 0.63 (s, 3H), 0.06 (s, 6H). ^13^C NMR (101 MHz, CDCl3) δ 209.6, 141.7, 121.0, 72.7,

63.9, 57.1, 50.2, 44.2, 42.9, 39.0, 37.5, 36.8, 32.2, 32.0, 32.0, 31.7, 26.1, 24.6, 23.0, 21.2, 19.6,

18.4, 13.4, -4.4. HRMS calcd. for C27H48O2Si ([M + H]^+^): 431.3345, found: 431.3337.

# 3β-(*tert*-Butyldimethylsilyloxy)-androst-5-en-17β-carboxylic acid (CRG006).


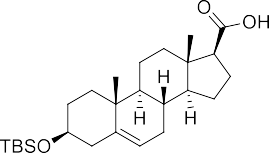


To a stirred solution of NaOH (6.41 g, 160.19 mmol) in water (55 mL) and dioxane (35 mL) was added dropwise Br2 (2.05 mL, 40.05 mmol) at 0 °C and the mixture was stirred at the same temperature for 10 min. 65 mL of this solution were added to an ice-cooled solution of **CRG005** (1.00 g, 2.32 mmol) in dioxane (65 mL) and water (8 mL) and the mixture was stirred overnight at rt. After cooling down to 0 °C, the reaction mixture was quenched with saturated aqueous Na2SO3, acidified with 1 M HCl and extracted with EtOAc. The combined organic layers were washed with brine, dried over anhydrous MgSO4, filtered and evaporated to dryness. Purification of the residue by flash chromatography (from 0 to 20% EtOAc in hexane) provided **CRG006** (830 mg, 83%) as a white solid.

[α]^20^ = –17.8 (*c* 1.0, CHCl3). ^1^H NMR (400 MHz, CDCl3) δ 5.31 (dd, *J* = 3.3, 1.9 Hz, 1H), 3.48 (ddd,

D

*J* = 15.7, 11.0, 4.8 Hz, 1H), 2.39 (t, *J* = 9.3 Hz, 1H), 2.33 – 0.92 (m, 19H), 1.00 (s, 3H), 0.89 (s, 9H),

0.74 (s, 3H), 0.06 (s, 6H). ^13^C NMR (101 MHz, CDCl3) δ 180.1, 141.8, 120.9, 72.7, 56.5, 55.3, 50.3,

44.3, 42.9, 38.2, 37.5, 36.8, 32.2, 32.0, 26.1, 24.7, 23.6, 21.0, 19.6, 18.4, 13.3, -4.4. HRMS calcd. for C26H43O3Si ([M – H]^–^): 431.2981, found: 431.2972.

# 3β-(*tert*-Butyldimethylsilyloxy)-17β-(hydroxymethyl)-androst-5-ene (CRG013).

To a stirred suspension of LiAlH4 (179 mg, 4.71 mmol) in THF (3 mL) was added dropwise a solution of **CRG006** (680 mg, 1.57 mmol) in THF (6 mL) at 0 °C. The reaction mixture was stirred at rt for 16 h, cooled down to 0 °C and quenched by the dropwise addition of water (10 mL). The resulting white slurry was filtered over celite and washed with Et2O. The combined filtrates were dried over anhydrous MgSO4, filtered and concentrated under reduced pressure to give the crude product. Purification by flash chromatography (from 0 to 12% EtOAc in hexane) afforded **CRG013** (515 mg, 78%) as a white solid.

[α]^20^ = –45.3 (*c* 1.0, CHCl3). ^1^H NMR (400 MHz, CDCl3) δ 5.32 (dd, *J* = 3.2, 2.1 Hz, 1H), 3.73 (dd,

D

*J* = 10.5, 6.9 Hz, 1H), 3.55 (dd, *J* = 10.5, 7.5 Hz, 1H), 3.48 (ddd, *J* = 15.6, 10.9, 4.7 Hz, 1H), 2.32 –

0.91 (m, 20H), 1.01 (s, 3H), 0.89 (s, 9H), 0.66 (s, 3H), 0.06 (s, 6H). ^13^C NMR (101 MHz, CDCl3) δ

141.8, 121.2, 72.8, 64.8, 56.4, 53.1, 50.6, 43.0, 41.8, 38.8, 37.6, 36.8, 32.2, 32.1, 31.8, 26.1, 25.7,

24.8, 20.9, 19.6, 18.4, 12.6, -4.4. HRMS calcd. for C26H47O2Si ([M + H]^+^): 419.3345, found:

419.3338.

# *N*-(2-(2-(2-(2-azidoethoxy)ethoxy)ethoxy)ethyl)-3β-(*tert*-Butyldimethylsilyloxy)-androst-5- en-17β-carboxamide (CRG007).


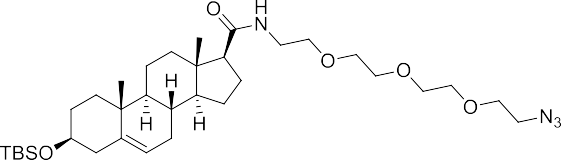


Compound **CRG007** (colourless oil, 382 mg, 80%) was obtained from **CRG006** (325 mg, 0.751 mmol), HOBt (122 mg, 0.901 mmol), EDC (173 mg, 0.901 mmol), **CRG003** (180 mg, 0.826 mmol) and TEA (157 µL, 1.13 mmol), according to general procedure 4. The title compound was purified by flash chromatography on silica gel (from 0 to 80% EtOAc in hexane).

[α]^20^ = –32.9 (*c* 1.0, CHCl3). ^1^H NMR (400 MHz, CDCl3) δ 5.77 (br s, 1H), 5.31 (dd, *J* = 3.5, 1.8 Hz,

D

1H), 3.76 – 3.58 (m, 10H), 3.57 – 3.41 (m, 5H), 3.39 (t, *J* = 4.0 Hz, 2H), 2.31 – 0.91 (m, 20H), 1.00

(s, 3H), 0.88 (s, 9H), 0.68 (s, 3H), 0.05 (s, 6H). ^13^C NMR (101 MHz, CDCl3) δ 172.8, 141.6, 121.0,

72.7, 70.9, 70.8, 70.7, 70.3, 70.3, 70.2, 57.1, 56.6, 50.8, 50.3, 43.8, 42.9, 39.1, 38.5, 37.5, 36.8,

32.2, 32.1, 32.0, 26.1, 24.7, 23.6, 21.1, 19.6, 18.4, 13.2, -4.46, -4.46. HRMS calcd. for C34H61N4O5Si ([M + H]^+^): 633.4411, found: 633.4409.

# *N*-(23-azido-3,6,9,12,15,18,21-heptaoxatricosyl)-3β-(*tert*-Butyldimethylsilyloxy)-androst-5- en-17β-carboxamide (CRG043).


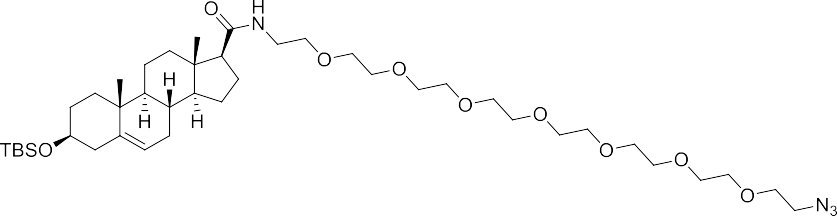


Compound **CRG043** (colourless oil, 274 mg, 92%) was obtained from **CRG006** (160 mg, 0.370 mmol), HOBt (60 mg, 0.444 mmol), EDC (85 mg, 0.444 mmol), **CRG042** (160 mg, 0.407 mmol) and TEA (77 µL, 0.555 mmol), according to general procedure 4. The title compound was purified by flash chromatography on silica gel (from 0 to 2.4% MeOH in CH2Cl2).

[α]^20^ = –27.2 (*c* 1.0, CHCl3). ^1^H NMR (400 MHz, CDCl3) δ 5.84 (br s, 1H), 5.31 (dd, *J* = 3.4, 1.8 Hz,

D

1H), 3.73 – 3.58 (m, 25H), 3.58 – 3.39 (m, 6H), 3.38 (t, *J* = 4.0 Hz, 2H), 2.32 – 0.91 (m, 20H), 0.99

(s, 3H), 0.88 (s, 9H), 0.68 (s, 3H), 0.05 (s, 6H). ^13^C NMR (101 MHz, CDCl3) δ 172.8, 141.6, 121.0,

72.6, 70.8, 70.8, 70.7, 70.7, 70.6, 70.6, 70.3, 70.3, 70.1, 57.1, 56.5, 50.8, 50.3, 43.8, 42.9, 39.1,

38.5, 37.5, 36.7, 32.1, 32.0, 32.0, 26.0, 24.7, 23.6, 21.1, 19.5, 18.3, 13.2, -4.5, -4.5. HRMS calcd. for C42H77N4O9Si ([M + H]^+^): 809.5460, found: 809.5480.

# *N*-(2-(2-(2-(2-azidoethoxy)ethoxy)ethoxy)ethyl)-3β-hydroxy-androst-5-en-17β-carboxamide (CRG008).


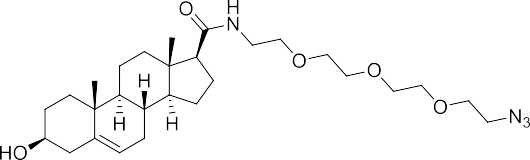


Compound **CRG008** (colourless oil, 279 mg, 92%) was obtained from **CRG007** (372 mg, 0.588 mmol) and TBAF (1.18 mL, 1.18 mmol), according to general procedure 5. The title compound was purified by flash chromatography on silica gel (from 0 to 100% EtOAc in hexane).

[α]^20^ = –45.0 (*c* 1.0, CHCl3). ^1^H NMR (400 MHz, CDCl3) δ 5.79 (br s, 1H), 5.35 (app d, *J* = 5.1 Hz,

D

1H), 3.77 – 3.58 (m, 10H), 3.58 – 3.41 (m, 5H), 3.39 (t, *J* = 4.0 Hz, 2H), 2.34 – 0.92 (m, 20H), 1.01

(s, 3H), 0.69 (s, 3H). ^13^C NMR (101 MHz, CDCl3) δ 172.8, 140.9, 121.4, 71.7, 70.8, 70.7, 70.7, 70.3,

70.2, 70.2, 57.1, 56.5, 50.7, 50.2, 43.8, 42.3, 39.1, 38.4, 37.4, 36.6, 32.0, 31.9, 31.7, 24.6, 23.6,

21.1, 19.5, 13.2. HRMS calcd. for C28H47N4O5 ([M + H]^+^): 519.3546, found: 519.3559.

#
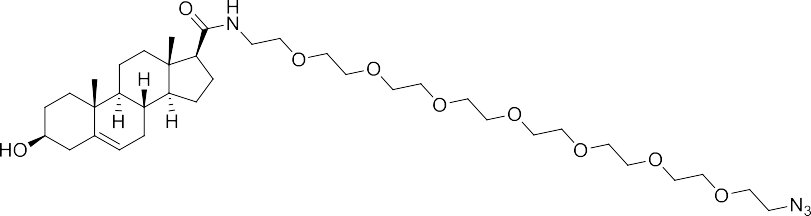
*N*-(23-azido-3,6,9,12,15,18,21-heptaoxatricosyl)-3β-hydroxy-androst-5-en-17β-carboxamide (CRG044).

Compound **CRG044** (colourless oil, 213 mg, 97%) was obtained from **CRG043** (255 mg, 0.315 mmol) and TBAF (630 µL, 0.630 mmol), according to general procedure 5. The title compound was purified by flash chromatography on silica gel (from 0 to 5% MeOH in CH2Cl2).

[α]^20^ = –33.1 (*c* 0.9, CHCl3). ^1^H NMR (400 MHz, CDCl3) δ 5.86 (br s, 1H), 5.35 (dd, *J* = 3.6, 1.7 Hz,

D

1H), 3.73 – 3.40 (m, 31H), 3.38 (t, *J* = 4.0 Hz, 2H), 2.34 – 0.92 (m, 20H), 1.01 (s, 3H), 0.68 (s, 3H).

^13^C NMR (101 MHz, CDCl3) δ 172.9, 140.9, 121.5, 71.7, 70.8, 70.8, 70.7, 70.7, 70.6, 70.6, 70.3,

70.2, 70.1, 57.1, 56.5, 50.8, 50.2, 43.8, 42.3, 39.1, 38.5, 37.4, 36.7, 32.0, 31.9, 31.7, 24.7, 23.6,

21.1, 19.5, 13.2. HRMS calcd. for C36H63N4O9 ([M + H]^+^): 695.4595, found: 695.4606.

# *N*-(2-(2-(2-(2-azidoethoxy)ethoxy)ethoxy)ethyl)-3-oxo-androst-4-en-17β-carboxamide (CRG009).


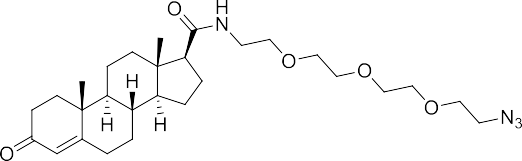


Compound **CRG009** (colourless oil, 162 mg, 63%) was obtained from **CRG008** (260 mg, 0.501 mmol) and Al(O*^t^*Bu)3 (185 mg, 0.752 mmol), according to general procedure 6. The title compound was purified by flash chromatography on silica gel (from 0 to 1.8% MeOH in CH2Cl2).

[α]^20^ = +71.8 (*c* 0.6, CHCl3). ^1^H NMR (400 MHz, CDCl3) δ 5.79 (br s, 1H), 5.73 (s, 1H), 3.76 – 3.58

D

(m, 10H), 3.57 – 3.40 (m, 4H), 3.38 (t, *J* = 4.0 Hz, 2H), 2.49 – 0.91 (m, 20H), 1.19 (s, 3H), 0.73 (s,

3H). ^13^C NMR (101 MHz, CDCl3) δ 199.5, 172.6, 171.1, 124.0, 70.8, 70.7, 70.7, 70.3, 70.2, 70.2,

57.0, 55.6, 53.9, 50.7, 43.7, 39.1, 38.7, 38.3, 35.8, 35.7, 34.0, 32.9, 32.0, 24.5, 23.6, 21.0, 17.5,

13.3. HRMS calcd. for C28H45N4O5 ([M + H]^+^): 517.3390, found: 517.3397.

#
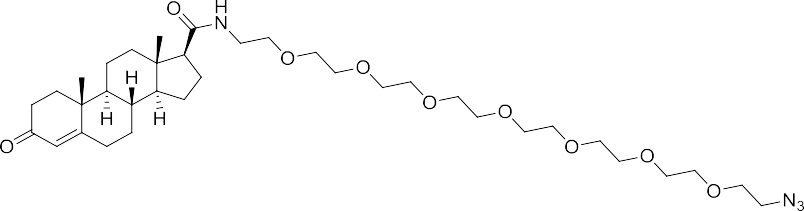
*N*-(23-azido-3,6,9,12,15,18,21-heptaoxatricosyl)-3-oxo-androst-4-en-17β-carboxamide (CRG045).

Compound **CRG045** (colourless oil, 98 mg, 58%) was obtained from **CRG044** (170 mg, 0.245 mmol) and Al(O*^t^*Bu)3 (90 mg, 0.367 mmol), according to general procedure 6. The title compound was purified by flash chromatography on silica gel (from 0 to 3% MeOH in CH2Cl2).

[α]^20^ = +53.2 (*c* 1.0, CHCl3). ^1^H NMR (400 MHz, CDCl3) δ 5.89 (br s, 1H), 5.73 (s, 1H), 3.71 – 3.58

D

(m, 25H), 3.58 – 3.41 (m, 5H), 3.38 (t, *J* = 4.0 Hz, 2H), 2.49 – 0.91 (m, 20H), 1.18 (s, 3H), 0.72 (s,

3H). ^13^C NMR (101 MHz, CDCl3) δ 199.6, 172.7, 171.2, 124.0, 70.8, 70,8, 70.7, 70.7, 70,7, 70,7,

70.6, 70.3, 70.3, 70.2, 57.0, 55.6, 53.9, 50.8, 43.8, 39.2, 38.7, 38.3, 35.9, 35.8, 34.1, 32.9, 32.1,

24.6, 23.6, 21.1, 17.5, 13.4. HRMS calcd. for C36H61N4O9 ([M + H]^+^): 693.4439, found: 693.4421.

#
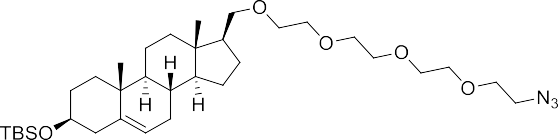
3β-(*tert*-Butyldimethylsilyloxy)-17β-(13-azido-2,5,8,11-tetraoxatridecyl)-androst-5-ene (CRG014).

Compound **CRG014** (colourless oil, 37 mg, 24%) was obtained from **CRG013** (104 mg, 0.248 mmol), **CRG012** (74 mg, 0.248 mmol) and NaH (20 mg, 0.497 mmol), according to general procedure 7. The title compound was purified by flash chromatography on silica gel (from 0 to 25% EtOAc in hexane).

[α]^20^ = –27.3 (*c* 1.0, CHCl3). ^1^H NMR (400 MHz, CDCl3) δ 5.31 (dd, *J* = 3.2, 2.0 Hz, 1H), 3.70 –

D

3.43 (m, 16H), 3.39 (t, *J* = 4.0 Hz, 2H), 3.33 (dd, *J* = 9.4, 7.4 Hz, 1H), 2.32 – 0.90 (m, 20H), 1.00 (s,

3H), 0.89 (s, 9H), 0.63 (s, 3H), 0.05 (s, 6H). ^13^C NMR (101 MHz, CDCl3) δ 141.7, 121.2, 73.2, 72.8,

70.9, 70.8, 70.8, 70.5, 70.2, 56.3, 50.8, 50.6, 49.9, 43.0, 41.8, 38.5, 37.6, 36.8, 32.2, 32.1, 31.8,

26.2, 26.1, 24.9, 20.9, 19.6, 18.4, 12.5, -4.4. HRMS calcd. for C34H61N3O5SiNa ([M + Na]^+^):

642.4278, found: 642.4277.

# 3β-(*tert*-Butyldimethylsilyloxy)-17β-(25-azido-2,5,8,11,14,17,20,23-octaoxapentacosyl)- androst-5-ene (CRG038).


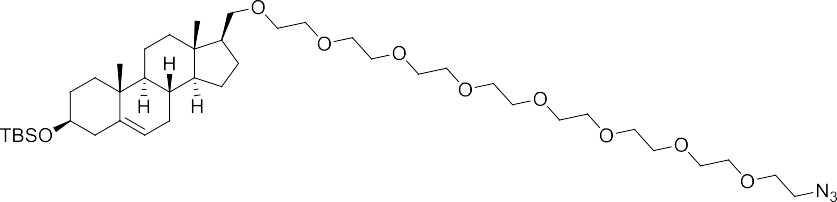


Compound **CRG038** (colourless oil, 135 mg, 53%) was obtained from **CRG013** (135 mg, 0.322 mmol), **CRG037** (177 mg, 0.322 mmol) and NaH (26 mg, 0.645 mmol), according to general procedure 7. The title compound was purified by flash chromatography on silica gel (from 0 to 100% EtOAc in hexane).

[α]^20^ = –22.3 (*c* 0.6, CHCl3). ^1^H NMR (400 MHz, CDCl3) δ 5.31 (dd, *J* = 3.2, 2.0 Hz, 1H), 3.70 –

D

3.42 (m, 32H), 3.39 (t, *J* = 4.0 Hz, 2H), 3.33 (dd, *J* = 9.4, 7.4 Hz, 1H), 2.31 – 0.89 (m, 20H), 1.00 (s,

3H), 0.88 (s, 9H), 0.63 (s, 3H), 0.05 (s, 6H). ^13^C NMR (101 MHz, CDCl3) δ 141.7, 121.2, 73.2, 72.7,

70.8, 70.8, 70.8, 70.8, 70.7, 70.7, 70.4, 70.2, 56.3, 50.8, 50.6, 49.9, 43.0, 41.8, 38.5, 37.5, 36.8,

32.2, 32.1, 31.8, 26.1, 26.1, 24.9, 20.9, 19.6, 18.4, 12.5, -4.5. HRMS calcd. for C42H77N3O9SiNa

([M + Na]+): 818.5327, found: 818.5339.

# 17β-(13-azido-2,5,8,11-tetraoxatridecyl)-androst-5-en-3β-ol (CRG015).


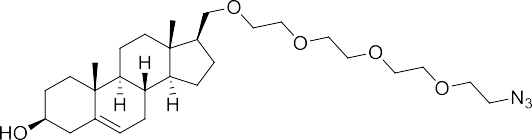


Compound **CRG015** (colourless oil, 81 mg, 86%) was obtained from **CRG014** (115 mg, 0.186 mmol) and TBAF (371 µL, 0.371 mmol), according to general procedure 5. The title compound was purified by flash chromatography on silica gel (from 0 to 45% EtOAc in hexane).

[α]^20^ = –35.2 (*c* 1.0, CHCl3). ^1^H NMR (400 MHz, CDCl3) δ 5.35 (dd, *J* = 3.3, 1.9 Hz, 1H), 3.74 –

D

3.44 (m, 16H), 3.39 (t, *J* = 5.1 Hz, 2H), 3.33 (dd, *J* = 9.4, 7.4 Hz, 1H), 2.34 – 0.78 (m, 20H), 1.01 (s,

3H), 0.63 (s, 3H). ^13^C NMR (101 MHz, CDCl3) δ 140.9, 121.7, 73.2, 71.8, 70.8, 70.8, 70.7, 70.4,

70.2, 56.3, 50.8, 50.5, 49.9, 42.4, 41.8, 38.5, 37.4, 36.7, 32.1, 31.8, 31.8, 26.1, 24.8, 20.9, 19.5,

12.5. HRMS calcd. for C28H47N3O5Na ([M + Na]^+^): 528.3413, found: 528.3419.

# 17β-(25-azido-2,5,8,11,14,17,20,23-octaoxapentacosyl)-androst-5-en-3β-ol (CRG039).


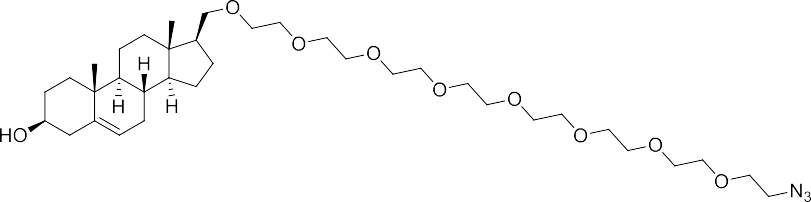


Compound **CRG039** (colourless oil, 53 mg, 76%) was obtained from **CRG038** (82 mg, 0.103 mmol) and TBAF (206 µL, 0.206 mmol), according to general procedure 5. The title compound was purified by flash chromatography on silica gel (from 0 to 4% MeOH in CH2Cl2).

[α]^20^ = –23.9 (*c* 0.7, CHCl3). ^1^H NMR (400 MHz, CDCl3) δ 5.33 (dd, *J* = 3.2, 2.0 Hz, 1H), 3.85 –

D

3.40 (m, 32H), 3.37 (t, *J* = 4.0 Hz, 2H), 3.32 (dd, *J* = 9.4, 7.4 Hz, 1H), 2.31 – 0.90 (m, 20H), 0.99 (s,

3H), 0.62 (s, 3H). ^13^C NMR (101 MHz, CDCl3) δ 140.9, 121.7, 73.2, 71.8, 70.8, 70.8, 70.7, 70.7,

70.7, 70.7, 70.7, 70.4, 70.1, 56.3, 50.8, 50.5, 49.9, 42.4, 41.8, 38.4, 37.4, 36.7, 32.1, 31.8, 31.8,

26.1, 24.8, 20.9, 19.5, 12.5. HRMS calcd. for C36H64N3O9 ([M + H]^+^): 682.4643, found: 682.4656.

# 17β-(13-azido-2,5,8,11-tetraoxatridecyl)-androst-4-en-3-one (CRG016).


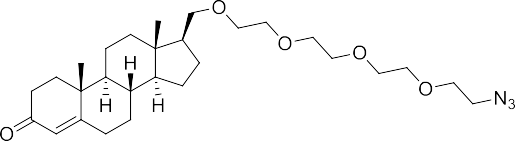


Compound **CRG016** (colourless oil, 62 mg, 89%) was obtained from **CRG015** (70 mg, 0.138 mmol) and Al(O*^t^*Bu)3 (51 mg, 0.208 mmol), according to general procedure 6. The title compound was purified by flash chromatography on silica gel (from 0 to 1% MeOH in CH2Cl2). [α]^20^ = +66.4 (*c* 1.0, CHCl3). ^1^H NMR (400 MHz, CDCl3) δ 5.72 (s, 1H), 3.73 – 3.47 (m, 15H), 3.39

D

(t, *J* = 4.0 Hz, 2H), 3.34 (dd, *J* = 9.4, 7.2 Hz, 1H), 2.48 – 0.90 (m, 20H), 1.18 (s, 3H), 0.67 (s, 3H).

^13^C NMR (101 MHz, CDCl3) δ 199.7, 171.6, 123.9, 73.1, 70.9, 70.8, 70.8, 70.5, 70.2, 55.4, 54.2,

50.8, 49.8, 41.9, 38.8, 38.4, 35.8, 35.5, 34.1, 33.1, 32.2, 26.0, 24.7, 20.9, 17.5, 12.5. HRMS calcd. for C28H46N3O5 ([M + H]^+^): 504.3437, found: 504.3435.

# 17β-(25-azido-2,5,8,11,14,17,20,23-octaoxapentacosyl)-androst-4-en-3-one (CRG046).


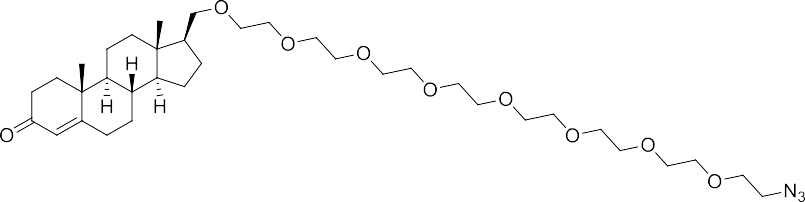


Compound **CRG046** (colourless oil, 75 mg, 92%) was obtained from **CRG039** (82 mg, 0.120 mmol) and Al(O*^t^*Bu)3 (44 mg, 0.180 mmol), according to general procedure 6. The title compound was purified by flash chromatography on silica gel (from 0 to 2% MeOH in CH2Cl2).

[α]^20^ = +53.3 (*c* 0.7, CHCl3). ^1^H NMR (400 MHz, CDCl3) δ 5.72 (s, 1H), 3.85 – 3.44 (m, 31H), 3.38

D

(t, *J* = 4.0 Hz, 2H), 3.34 (dd, *J* = 9.4, 7.2 Hz, 1H), 2.48 – 0.90 (m, 20H), 1.18 (s, 3H), 0.66 (s, 3H).

^13^C NMR (101 MHz, CDCl3) δ 199.7, 171.6, 123.9, 73.1, 70.8, 70.8, 70.7, 70.7, 70.7, 70.7, 70.7,

70.4, 70.1, 55.4, 54.2, 50.8, 49.8, 41.9, 38.8, 38.3, 35.8, 35.5, 34.1, 33.0, 32.2, 25.9, 24.7, 20.9,

17.5, 12.5. HRMS calcd. for C36H61N3O9Na ([M + H]^+^): 702.4306, found: 702.4361.

# (*R*)-Glycidol trityl ether (CRG023)


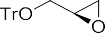


(*S*)-(-)-Glycidol (250 mg, 3.37 mmol) was added dropwise to a stirred solution of trityl chloride (1.03 g, 3.71 mmol) and TEA (517 µL, 3.71 mmol) in CH2Cl2 (3.5 mL) at 0 °C. After the addition was complete, the mixture was allowed to warm to rt and was stirred overnight. The reaction mixture was then poured into saturated aq. NH4Cl (10 mL) and the resulting mixture was extracted with CH2Cl2. The organic extracts were washed with brine, dried over anhydrous MgSO4, filtered and concentrated under reduced pressure. Flash chromatography of the residue (from 0 to 5% EtOAc in hexane) gave **CRG023** as a white solid (986 mg, 92%).

D

[α]20

D

= +11.0 (*c* 1.0, CHCl3) [lit.^5^ [α]^23^

= +11.2 (*c* 1.0, CHCl3]. ^1^H NMR (400 MHz, CDCl3) δ 7.50 –

7.42 (m, 6H), 7.33 – 7.21 (m, 9H), 3.37 – 3.28 (m, 1H), 3.19 – 3.09 (m, 2H), 2.81 – 2.75 (m, 1H),

2.63 (dd, *J* = 5.1, 2.3 Hz, 1H). ^13^C NMR (101 MHz, CDCl3) δ 143.9, 128.8, 128.0, 127.2, 86.8, 64.9,

51.2, 44.8. HRMS calcd. for C22H20O2Na ([M + Na]^+^): 339.1361, found: 339.1346.

# (*R*)-1-(hexadecyloxy)-3-(trityloxy)propan-2-ol (CRG024).

To a solution of **CRG023** (640 mg, 2.02 mmol) in DMF (15 mL) were added portionwise KO*t*Bu (681 mg, 6.07 mmol) and hexadecanol (736 mg, 3.03 mmol) at rt. The resulting heterogeneous mixture was stirred at 80 °C for 1h. After cooling down to rt, water (15 mL) was added and the resulting mixture was extracted with Et2O. The combined organic layers were washed with brine, dried over anhydrous MgSO4, filtered and evaporated to dryness. Purification of the residue by flash chromatography (from 0 to 18% Et2O in hexane) afforded **CRG024** as a white solid (623 mg, 55%).

D

[α]20

D

= +3.1 (*c* 1.0, CHCl3) [lit.^6^ [α]^26^

= +2.3 (*c* 1.02, CHCl3]. ^1^H NMR (400 MHz, CDCl3) δ 7.46 –

7.41 (m, 6H), 7.33 – 7.20 (m, 9H), 3.99 – 3.90 (m, 1H), 3.54 (dd, *J* = 9.7, 4.3 Hz, 1H), 3.50 – 3.38

(m, 3H), 3.25 – 3.14 (m, 2H), 2.41 (d, *J* = 4.6 Hz, 1H), 1.58 – 1.49 (m, 2H), 1.37 – 1.16 (m, 26H),

0.88 (t, *J* = 6.8 Hz, 3H). ^13^C NMR (101 MHz, CDCl3) δ 144.0, 128.8, 128.0, 127.2, 86.8, 72.2, 71.8,

70.0, 64.8, 32.1, 29.9, 29.8, 29.8, 29.8, 29.8, 29.7, 29.5, 26.3, 22.9, 14.3. HRMS calcd. for C38H54O3Na ([M + Na]^+^): 581.3971, found: 581.3977.

# (*R*)-9-(diethylamino)-2-((8-((1-(hexadecyloxy)-3-(trityloxy)propan-2-yl)oxy)octyl)oxy)-5*H*- benzo[*a*]phenoxazin-5-one (CRG029).


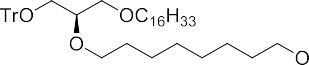


To a solution of **CRG024** (300 mg, 0.537 mmol) in DMF (5.5 mL) was added portionwise NaH (60 wt.% in mineral oil, 64mg, 1.61 mmol) at 0 °C and the mixture was stirred at the same temperature for 10 min. 1,8-dibromooctane (494 µL, 2.68 mmol) was then added dropwise and the resulting mixture was stirred at 65 °C overnight. After cooling down to rt, the reaction mixture was carefully diluted with water and extracted with Et2O. The combined organic layers were washed with brine, dried over anhydrous MgSO4, filtered and concentrated under reduced pressure. The residue was dissolved in CH2Cl2 and was filtered through a short pad of silica gel firstly eluting with hexane in order to remove the excess of 1,8-dibromooctane and then with hexane/EtOAc (95:5), affording the corresponding bromo derivative **5** as a crude intermediate. The resulting residue was dissolved in DMF (2 mL) and was directly added to a solution of 2-hydroxy Nile Red^1^ (216 mg, 0.645 mmol) and K2CO3 (149 mg, 1.07 mmol) in DMF (4 mL) at rt. The reaction mixture was stirred overnight at 65 °C, cooled to rt and diluted with water (10 mL). The resulting dark red solution was extracted with Et2O (5x) and the combined organic layers were washed with brine, dried over anhydrous MgSO4, filtered and evaporated to dryness. Purification of the residue by flash chromatography (from 0 to 40% EtOAc in hexane) gave **CRG029** as a dark violet oil (289 mg, 54% over two steps).

^1^H NMR (400 MHz, CDCl3) δ 8.18 (d, *J* = 8.7 Hz, 1H), 8.01 (d, *J* = 2.6 Hz, 1H), 7.56 (d, *J* = 9.0 Hz,

1H), 7.49 – 7.37 (m, 6H), 7.30 – 7.16 (m, 9H), 7.12 (dd, *J* = 8.7, 2.6 Hz, 1H), 6.61 (dd, *J* = 9.1, 2.7

Hz, 1H), 6.42 (d, *J* = 2.7 Hz, 1H), 6.27 (s, 1H), 4.10 (t, *J* = 6.5 Hz, 2H), 3.59 – 3.34 (m, 11H), 3.20 –

3.10 (m, 2H), 1.86 – 1.77 (m, 2H), 1.63 – 1.12 (m, 44H), 0.85 (t, *J* = 6.8 Hz, 3H). 13C NMR (101

MHz, CDCl3) δ 183.4, 162.0, 152.2, 150.8, 147.0, 144.3, 140.3, 134.2, 131.2, 128.9, 127.8, 127.0,

125.6, 124.9, 118.4, 109.6, 106.8, 105.4, 96.5, 86.6, 78.5, 71.8, 71.3, 70.8, 68.5, 63.8, 45.2, 32.1,

30.3, 29.8, 29.8, 29.8, 29.7, 29.6, 29.6, 29.5, 29.4, 26.3, 26.3, 26.2, 22.8, 14.3, 12.8. HRMS calcd. for C66H87N2O6 ([M + H]^+^): 1003.6564, found: 1003.6587.

# (*S*)-9-(diethylamino)-2-((8-((1-(hexadecyloxy)-3-hydroxypropan-2-yl)oxy)octyl)oxy)-5*H*- benzo[*a*]phenoxazin-5-one (CRG031).


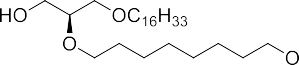


To an ice-cooled solution of **CRG029** (160 mg, 0.160 mmol) in MeOH (3 mL) and CHCl3 (250 µL), *p*-TsOH (55 mg, 0.319 mmol) was added in one portion. After stirring at rt for 2.5 h, the reaction was carefully quenched with saturated aqueous NaHCO3 (15 mL) and the resulting mixture was extracted with CH2Cl2. The combined organic extracts were washed with brine, dried over anhydrous MgSO4, filtered and evaporated *in vacuo*. The residue was purified by flash chromatography (from 0 to 70% EtOAc in hexane) to yield **CRG031** (110 mg, 91%) as a dark violet oil.

^1^H NMR (400 MHz, CDCl3) δ 8.19 (d, *J* = 8.7 Hz, 1H), 8.01 (d, *J* = 2.6 Hz, 1H), 7.57 (d, *J* = 9.0 Hz,

1H), 7.14 (dd, *J* = 8.7, 2.6 Hz, 1H), 6.62 (dd, *J* = 9.1, 2.7 Hz, 1H), 6.42 (d, *J* = 2.7 Hz, 1H), 6.27 (s,

1H), 4.15 (t, *J* = 6.5 Hz, 2H), 3.72 (dd, *J* = 11.3, 3.7 Hz, 1H), 3.66 – 3.57 (m, 2H), 3.57 – 3.37 (m,

10H), 1.90 – 1.80 (m, 2H), 1.68 – 1.09 (m, 44H), 0.87 (t, *J* = 6.9 Hz, 3H). ^13^C NMR (101 MHz, CDCl3)

δ 183.4, 162.0, 152.1, 150.8, 146.9, 140.2, 134.2, 131.2, 127.8, 125.6, 124.8, 118.4, 109.6, 106.7,

105.4, 96.4, 78.4, 72.0, 71.0, 70.5, 68.5, 63.2, 45.2, 32.1, 30.2, 29.8, 29.8, 29.8, 29.8, 29.7, 29.6,

29.5, 29.5, 29.4, 26.2, 26.2, 26.2, 22.8, 14.3, 12.8. HRMS calcd. for C47H73N2O6 ([M + H]^+^):

761.5469, found: 761.5490.

# (*R*)-2-((8-((9-(diethylamino)-5-oxo-5*H*-benzo[*a*]phenoxazin-2-yl)oxy)octyl)oxy)-3- (hexadecyloxy)propyl (2-(dimethyl(prop-2-yn-1-yl)ammonio)ethyl) phosphate (CRG032).


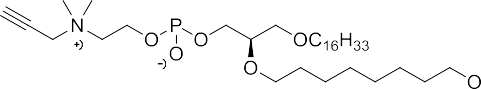


A solution of **CRG031** (110 mg, 0.145 mmol) in toluene (1.5 mL) at 0 °C was successively treated with TEA (60 µL, 0.434 mmol), DMAP (4 mg, 0.029 mmol) and 2-chloro-1,3,2-dioxaphospholane 2-oxide (40 µL, 0.434 mmol). After stirring at 0 °C for 1.5 h, the reaction mixture was transferred to a sealed tube *via* cannula under positive nitrogen pressure. The crude solution was diluted with MeCN (13 mL) before 3-dimethylamino-1-propyne (621 µL, 5.77 mmol) was added dropwise. The tube was flushed with argon, sealed and the mixture stirred at 80 °C for 48 h. The reaction mixture was then transferred to a round-bottom flask, concentrated *in vacuo* and purified by flash chromatography on silica gel (from 90:10:0 to 67:30:3 CHCl3/MeOH/H2O), yielding the zwitterionic amino phosphate **CRG032** (58 mg, 42% over two steps) as a dark violet oil.

^1^H NMR (400 MHz, CD3OD/CD3CN (1:1)) δ 8.09 (d, *J* = 8.8 Hz, 1H), 8.00 (s, 1H), 7.58 (d, *J* = 9.1

Hz, 1H), 7.18 (d, *J* = 8.8 Hz, 1H), 6.83 (d, *J* = 7.1 Hz, 1H), 6.56 (s, 1H), 6.20 (s, 1H), 4.37 (d, *J* = 2.5

Hz, 2H), 4.28 (br s, 2H), 4.17 (t, *J* = 6.4 Hz, 2H), 3.89 (td, *J* = 10.5, 5.3 Hz, 2H), 3.73 – 3.67 (m, 2H),

3.67 – 3.40 (m, 12H), 3.25 (s, 6H), 1.92 – 1.82 (m, 2H), 1.63 – 1.18 (m, 44H), 0.89 (t, *J* = 6.9 Hz,

4H). ^13^C NMR (101 MHz, CD3OD/CD3CN (1:1)) δ 184.0, 163.0, 153.5, 152.6, 147.9, 139.3, 135.2,

132.1, 128.2, 126.0, 125.9, 119.0, 111.6, 107.2, 104.8, 97.0, 83.0, 79.1 (d, *J*C-P = 7.9 Hz), 72.4,

72.2, 71.4, 71.2, 69.3, 66.2 (d, *J*C-P = 5.1 Hz), 65.2 (d, *J*C-P = 6.8 Hz), 60.0 (d, *J*C-P = 4.2 Hz), 56.3,

52.1, 46.0, 32.8, 31.0, 30.7, 30.6, 30.6, 30.6, 30.4, 30.4, 30.3, 30.1, 27.1, 27.0, 26.9, 23.5, 14.4,

12.9, 9.2. ^31^P NMR (162 MHz, CD3OD/CD3CN (1:1)) δ 0.69. HRMS calcd. for C54H85N3O9P ([M +

H]+): 950.6023, found: 950.6133.

# Probe CRG033.


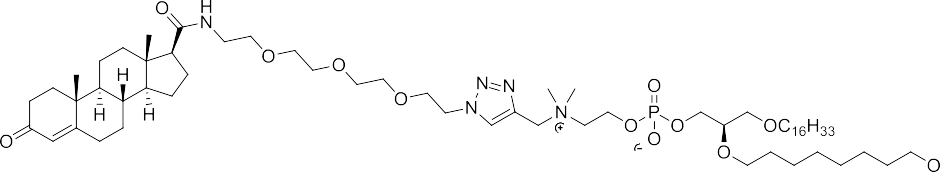


Compound **CRG033** (dark violet oil, 19 mg, 95%) was obtained from **CRG032** (13 mg, 0.014 mmol), **CRG009** (8 mg, 0.016 mmol), DIPEA (5 µL, 0.027 mmol) and CuI (0.391 mg, 0.002 mmol), according to general procedure 8. The title compound was purified by flash chromatography on silica gel (CHCl3/MeOH/H2O (78:20:2)).

^1^H NMR (400 MHz, CD3OD/CD3CN (1:1)) δ 8.32 (s, 1H), 8.06 (d, *J* = 8.8 Hz, 1H), 7.95 (d, *J* = 2.5

Hz, 1H), 7.54 (d, *J* = 9.1 Hz, 1H), 7.14 (dd, *J* = 8.8, 2.5 Hz, 1H), 7.09 – 7.02 (m, 1H), 6.79 (dd, *J* =

9.2, 2.6 Hz, 1H), 6.52 (d, *J* = 2.6 Hz, 1H), 6.16 (s, 1H), 5.66 (s, 1H), 4.73 (s, 2H), 4.63 (t, *J* = 5.1 Hz,

2H), 4.34 (br s, 2H), 4.14 (t, *J* = 6.5 Hz, 2H), 3.94 – 3.85 (m, 4H), 3.68 – 3.36 (m, 24H), 3.29 – 3.22

(m, 1H), 3.17 (s, 6H), 2.50 – 0.83 (m, 66H), 1.18 (s, 3H), 0.88 (t, *J* = 6.9 Hz, 3H), 0.68 (s, 3H). 13C NMR (101 MHz, CD3OD/CD3CN (1:1)) δ 201.5, 184.4, 174.7, 174.2, 163.1, 153.7, 152.6, 148.0,

139.5, 136.2, 135.3, 132.2, 130.1, 128.4, 126.1, 125.9, 124.1, 119.1, 111.6, 107.4, 105.0, 97.0,

79.1 (d, *J*C-P = 7.9 Hz), 72.4, 71.5, 71.3, 71.2, 71.2, 71.0, 70.5, 69.9, 69.4, 66.1 (d, *J*C-P = 5.1 Hz),

64.9 (d, *J*C-P = 9.1 Hz), 60.4, 60.0 (d, *J*C-P = 4.2 Hz), 57.4, 56.4, 54.9, 52.0, 51.4, 46.0, 44.9, 39.9,

39.7, 38.8, 36.5, 34.6, 33.6, 33.0, 32.9, 31.0, 30.7, 30.6, 30.6, 30.4, 30.3, 30.3, 30.3, 30.1, 27.1,

27.0, 26.9, 25.3, 24.3, 23.6, 21.8, 17.7, 17.7, 14.4, 13.8, 12.9. ^31^P NMR (162 MHz, CD3OD/CD3CN

(1:1)) δ 0.35. HRMS calcd. for C82H129N7O14P ([M + H]^+^): 1466.9335, found: 1466.9370.

# Probe CRG047.


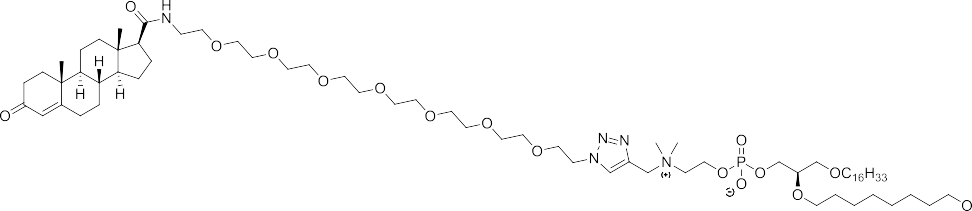


Compound **CRG047** (dark violet oil, 22 mg, 61%) was obtained from **CRG032** (25 mg, 0.026 mmol), **CRG045** (21 mg, 0.030 mmol), DIPEA (9 µL, 0.053 mmol) and CuI (0.752 mg, 0.004 mmol), according to general procedure 8. The title compound was purified by flash chromatography on silica gel (CHCl3/MeOH/H2O (78:20:2)).

^1^H NMR (400 MHz, CD3OD) δ 8.40 (s, 1H), 8.04 (d, *J* = 8.7 Hz, 1H), 7.94 (d, *J* = 2.4 Hz, 1H), 7.54

(d, *J* = 9.1 Hz, 1H), 7.51 – 7.45 (m, 1H), 7.12 (dd, *J* = 8.8, 2.5 Hz, 1H), 6.79 (dd, *J* = 9.2, 2.6 Hz, 1H),

6.53 (d, *J* = 2.6 Hz, 1H), 6.16 (s, 1H), 5.66 (s, 1H), 4.77 (s, 2H), 4.67 – 4.62 (m, 2H), 4.36 (br s, 2H),

4.12 (t, *J* = 6.3 Hz, 2H), 3.95 – 3.87 (m, 4H), 3.71 – 3.35 (m, 40H), 3.29 – 3.23 (m, 1H), 3.20 (s,

6H), 2.50 – 0.82 (m, 66H), 1.18 (s, 3H), 0.87 (t, *J* = 6.9 Hz, 3H), 0.68 (s, 3H). 13C NMR (101 MHz,

CD3OD) δ 202.0, 184.9, 175.2, 174.8, 163.4, 153.9, 152.9, 148.3, 139.5, 136.5, 135.5, 132.5,

130.5, 128.5, 126.3, 124.2, 119.3, 111.8, 107.5, 105.0, 97.2, 79.4 (d, *J*C-P = 4.0 Hz), 72.6, 71.8,

71.6, 71.6, 71.5, 71.5, 71.4, 71.4, 71.2, 70.8, 70.2, 69.5, 66.5 (d, *J*C-P = 5.1 Hz), 65.0 (d, *J*C-P = 5.1

Hz), 60.7, 60.3 (d, *J*C-P = 3.0 Hz), 57.6, 56.7, 55.3, 52.0, 51.6, 46.2, 45.1, 40.3, 39.9, 39.0, 36.8,

36.8, 34.7, 33.9, 33.2, 33.1, 31.2, 30.9, 30.8, 30.8, 30.8, 30.7, 30.6, 30.5, 30.4, 27.3, 27.2, 27.2,

25.5, 24.5, 23.8, 22.0, 17.7, 14.5, 13.9, 13.0. ^31^P NMR (162 MHz, CD3OD) δ 0.69. HRMS calcd. for C90H146N7O18P ([M + 2H]^2+^): 822.0231, found: 822.0209.

# Probe CRG034.


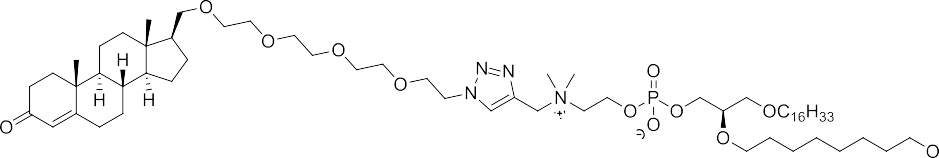


Compound **CRG034** (dark violet oil, 15 mg, 75%) was obtained from **CRG032** (13 mg, 0.014 mmol), **CRG016** (8 mg, 0.016 mmol), DIPEA (5 µL, 0.027 mmol) and CuI (0.391 mg, 0.002 mmol), according to general procedure 8. The title compound was purified by flash chromatography on silica gel (CHCl3/MeOH/H2O (83.5:15:1.5)).

^1^H NMR (400 MHz, CD3OD) δ 8.39 (s, 1H), 8.04 (d, *J* = 8.8 Hz, 1H), 7.93 (d, *J* = 2.5 Hz, 1H), 7.53

(d, *J* = 9.1 Hz, 1H), 7.12 (dd, *J* = 8.8, 2.6 Hz, 1H), 6.78 (dd, *J* = 9.2, 2.7 Hz, 1H), 6.52 (d, *J* = 2.6 Hz,

1H), 6.15 (s, 1H), 5.63 (s, 1H), 4.76 (s, 2H), 4.67 – 4.62 (m, 2H), 4.35 (br s, 2H), 4.12 (t, *J* = 6.4 Hz,

2H), 3.95 – 3.87 (m, 4H), 3.67 – 3.40 (m, 26H), 3.29 – 3.23 (m, 1H), 3.20 (s, 6H), 2.48 – 0.75 (m,

66H), 1.15 (s, 3H), 0.87 (t, *J* = 6.9 Hz, 3H), 0.62 (s, 3H). ^13^C NMR (101 MHz, CD3OD) δ 202.1, 184.8,

174.9, 163.4, 153.9, 152.9, 148.2, 139.5, 136.5, 135.5, 132.5, 130.5, 128.5, 126.3, 126.2, 124.2,

119.3, 111.8, 107.5, 105.0, 97.2, 79.4 (d, *J*C-P = 8.1 Hz), 73.9, 72.6, 71.8, 71.6, 71.6, 71.5, 71.5,

71.4, 70.2, 69.5, 66.4 (d, *J*C-P = 6.1 Hz), 64.9 (d, *J*C-P = 8.1 Hz), 60.6, 60.3 (d, *J*C-P = 5.1 Hz), 56.6,

55.5, 52.0, 51.6, 51.0, 46.2, 42.9, 39.9, 39.5, 36.7, 36.5, 34.7, 33.9, 33.3, 33.1, 31.2, 30.9, 30.9,

30.8, 30.8, 30.7, 30.7, 30.6, 30.5, 30.4, 27.4, 27.2, 27.2, 26.8, 25.5, 23.8, 21.9, 17.7, 14.5, 13.0,

12.8. ^31^P NMR (162 MHz, CD3OD) δ 0.72. HRMS calcd. for C82H130N6O14P ([M + H]^+^): 1453.9383,

found: 1453.9371.

# Probe CRG048.


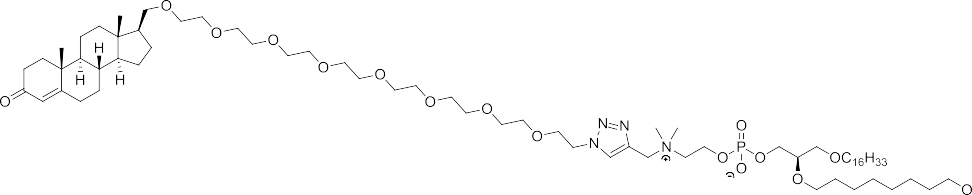


Compound **CRG048** (dark violet oil, 34 mg, 79%) was obtained from **CRG032** (25 mg, 0.026 mmol), **CRG046** (21 mg, 0.030 mmol), DIPEA (9 µL, 0.053 mmol) and CuI (0.752 mg, 0.004 mmol), according to general procedure 8. The title compound was purified by flash chromatography on silica gel (CHCl3/MeOH/H2O (78.5:20:1.5)).

^1^H NMR (400 MHz, CD3OD) δ 8.40 (s, 1H), 8.06 (d, *J* = 8.8 Hz, 1H), 7.98 (d, *J* = 2.5 Hz, 1H), 7.57

(d, *J* = 9.1 Hz, 1H), 7.15 (dd, *J* = 8.8, 2.6 Hz, 1H), 6.81 (dd, *J* = 9.2, 2.6 Hz, 1H), 6.56 (d, *J* = 2.6 Hz,

1H), 6.18 (s, 1H), 5.65 (s, 1H), 4.76 (s, 2H), 4.67 – 4.62 (m, 2H), 4.36 (br s, 2H), 4.14 (t, *J* = 6.4 Hz,

2H), 3.96 – 3.87 (m, 4H), 3.71 – 3.38 (m, 41H), 3.20 (s, 6H), 2.49 – 2.37 (m, 66H), 1.17 (s, 3H),

0.87 (t, *J* = 6.9 Hz, 3H), 0.65 (s, 3H). ^13^C NMR (101 MHz, CD3OD) δ 202.1, 184.8, 175.0, 163.4,

153.9, 152.9, 148.2, 139.5, 136.5, 135.5, 132.5, 130.5, 129.9, 128.5, 126.3, 124.2, 119.2, 111.8,

107.5, 105.0, 97.2, 79.4 (d, *J*C-P = 9.1 Hz), 73.9, 72.6, 71.8, 71.6, 71.6, 71.6, 71.6, 71.5, 71.5, 71.4,

71.4, 70.2, 69.5, 66.4 (d, *J*C-P = 5.1 Hz), 65.0 (d, *J*C-P = 5.1 Hz), 60.7, 60.3 (d, *J*C-P = 4.1 Hz), 56.6,

55.6, 52.0, 51.6, 51.0, 46.2, 42.9, 40.0, 39.5, 36.7, 36.6, 34.7, 33.9, 33.3, 33.1, 31.3, 30.9, 30.9,

30.8, 30.8, 30.7, 30.6, 30.5, 30.4, 27.4, 27.2, 27.2, 26.8, 25.5, 23.8, 21.9, 17.7, 14.5, 13.0, 12.8.

^31^P NMR (162 MHz, CD3OD) δ 0.79. HRMS calcd. for C90H147N6O18P ([M + 2H]^2+^): 815.5255,

found: 815.5259.

# 3-oxo-androst-4-en-17β-carboxylic acid (CRG049).

To a stirred solution of NaOH (827 mg, 20.67 mmol) in water (7 mL) was added dropwise Br2 (277 µL, 5.41 mmol) at 0 °C and the mixture was stirred at the same temperature for 10 min. The resulting yellowish solution was added dropwise to an ice-cooled solution of progesterone

(**6**) (500 mg, 1.59 mmol) in dioxane (15 mL) and water (5 mL) and the mixture was stirred at rt for 1.5 h. A saturated aqueous Na2SO3 solution (5 mL) was added dropwise until the solution turned colourless and the resulting mixture was refluxed for 15 min. After cooling down to 0 °C, the pH of the solution was adjusted to 2-3 with 1 M HCl. Dioxane was carefully evaporated under reduced pressure and the resulting precipitate was filtered, washed with water, and

dried to give the crude product. Purification by flash chromatography (from 0 to 70% EtOAc in hexane) provided **CRG049** (372 mg, 74%) as a pale-yellow solid.

D

[α]20

D

= +155.5 (*c* 1.0, CHCl3) [lit.^7^ [α]^25^

= +140.3 (*c* 1.0, CHCl3]. ^1^H NMR (400 MHz, CDCl3) δ 5.74

(s, 3H), 2.49 – 0.92 (m, 20H), 1.19 (s, 3H), 0.78 (s, 3H). ^13^C NMR (101 MHz, CDCl3) δ 199.8, 179.5,

171.3, 124.1, 55.5, 55.1, 53.8, 44.2, 38.8, 38.0, 35.9, 35.8, 34.1, 32.9, 32.0, 24.5, 23.5, 21.0, 17.5,

13.4. HRMS calcd. for C20H29O3 ([M + H]^+^): 317.2117, found: 317.2116.

# Fluorescence quantum yield measurements

Relative quantum yields were measured on a SpectraMax M5 (Molecular Devices) spectrophotometer using a 1 cm path length quartz cuvette. Rhodamine B was obtained from Sigma-Aldrich and was used without further purification. Absolute ethanol (analytical quality) was deoxygenated prior to use. Fluorescence quantum yields were calculated using the following equation:

*F* ·β ·η 2

Φ *S* = *S ref S* ·Φ*ref*

·η

*F*

*F*

*ref*

·β*S*

2 *F*

*ref*

Where Φ*^S^* and Φ*^ref^* are the fluorescence quantum yield of the sample and that of the standard

*F*

*F*

(Rhodamine B, Φ*^ref^* = 0.7),^8^ respectively. FS and Fref represent the area of fluorescent emission

*F*

in units of photons. η*S*

and η*ref*

are the refractive indices of the solvent used (ethanol, η =

1.361). β*S* and β*ref*

are the correction absorption factors, β = 1 −10^−^ *^A^* where A = absorbance.

In order to minimize reabsorption effects, the solutions for quantum yield measurements were prepared such that the optical density was generally about 0.04 at λex = 550 nm.

# Supporting information (organic synthesis) references

1. S. J. Briggs, M.; Bruce, I.; N. Miller, J.; J. Moody, C.; C. Simmonds, A.; Swann, E. Synthesis of functionalised fluorescent dyes and their coupling to amines and amino acids. *J. Chem. Soc. Perkin Trans. 1* **1997**, No. 7, 1051–1058.
2. Goretta, S. A.; Kinoshita, M.; Mori, S.; Tsuchikawa, H.; Matsumori, N.; Murata, M. Effects of chemical modification of sphingomyelin ammonium group on formation of liquid- ordered phase. *Bioorg. Med. Chem.* **2012**, *20* (13), 4012–4019.
3. Sandbhor, M. S.; Key, J. A.; Strelkov, I. S.; Cairo, C. W. A Modular Synthesis of Alkynyl- Phosphocholine Headgroups for Labeling Sphingomyelin and Phosphatidylcholine. *J. Org. Chem.* **2009**, *74* (22), 8669–8674.
4. Lao, K.; Sun, J.; Wang, C.; Lyu, W.; Zhou, B.; Zhao, R.; Xu, Q.; You, Q.; Xiang, H. Design, synthesis and biological evaluation of novel androst-3,5-diene-3-carboxylic acid derivatives as inhibitors of 5α-reductase type 1 and 2. *Steroids* **2017**, *124*, 29–34.
5. White, J. D.; Lincoln, C. M.; Yang, J.; Martin, W. H. C.; Chan, D. B. Total Synthesis of Solandelactones A, B, E, and F Exploiting a Tandem Petasis−Claisen Lactonization Strategy. *J. Org. Chem.* **2008**, *73* (11), 4139–4150.
6. Nakamura, N.; Miyazaki, H.; Ohkawa, N.; Oshima, T.; Koike, H. An efficient synthesis of platelet-activating factor (PAF) via 1-O-alkyl-2-O-(3-isoxazolyl)-SN-glycero-3-phospho- choline, a new PAF agonist utilization of the 3-isoxazolyloxy group as a protected

hydroxyl. *Tetrahedron Lett.* **1990**, *31* (5), 699–702.

1. Rey, J.; O’Riordan, T. J. C.; Hu, H.; Snyder, J. P.; White, A. J. P.; Barrett, A. G. M. Design and Diastereoselective Synthesis of C-2,C-20-Diaryl Steroidal Derivatives. *Eur. J. Org. Chem.* **2012**, *2012* (20), 3781–3794.
2. Arbeloa, F. L.; Ojeda, P. R.; Arbeloa, I. L. Flourescence self-quenching of the molecular forms of Rhodamine B in aqueous and ethanolic solutions. *J. Lumin.* **1989**, *44* (1), 105– 112.

**Supplementary Figure S1**

**Supplementary Figure S2**

**Supplementary figure S3**

**Supplementary figure S4**

**Supplementary figure S5**

**Supplementary figure S6**

**Supplementary figure S7**

**Supplementary figure S8**

**Supplementary figure S9**


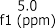

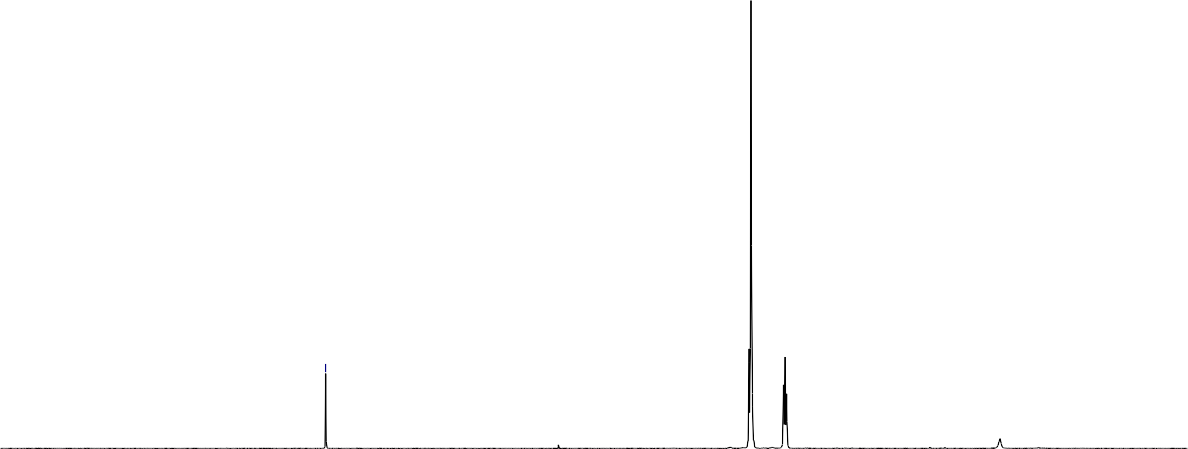


**a**


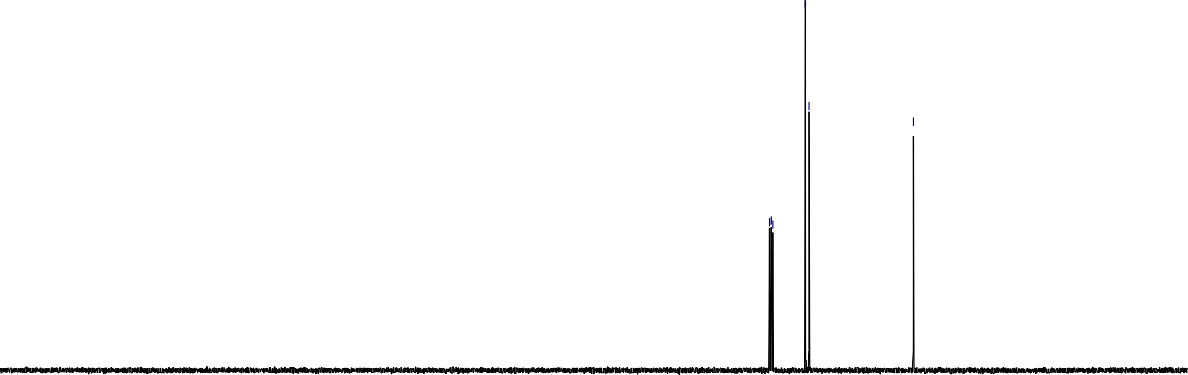


**b**

**Supplementary figure S10**


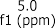

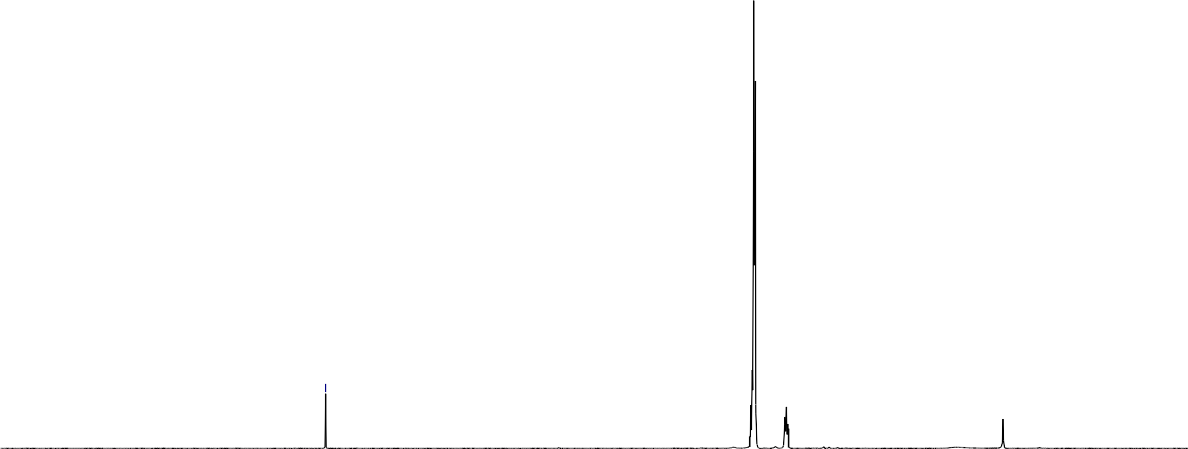

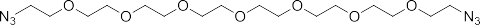


**a**


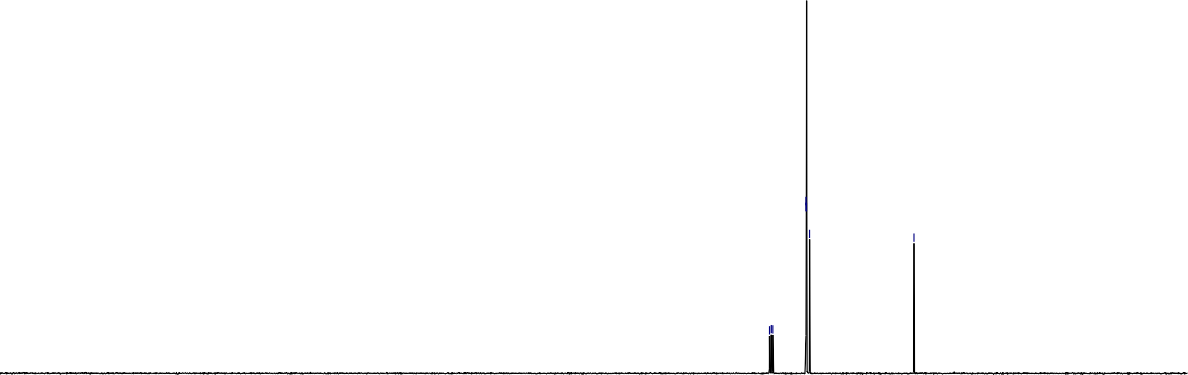

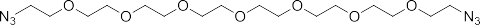


**b**

**Supplementary figure S11**


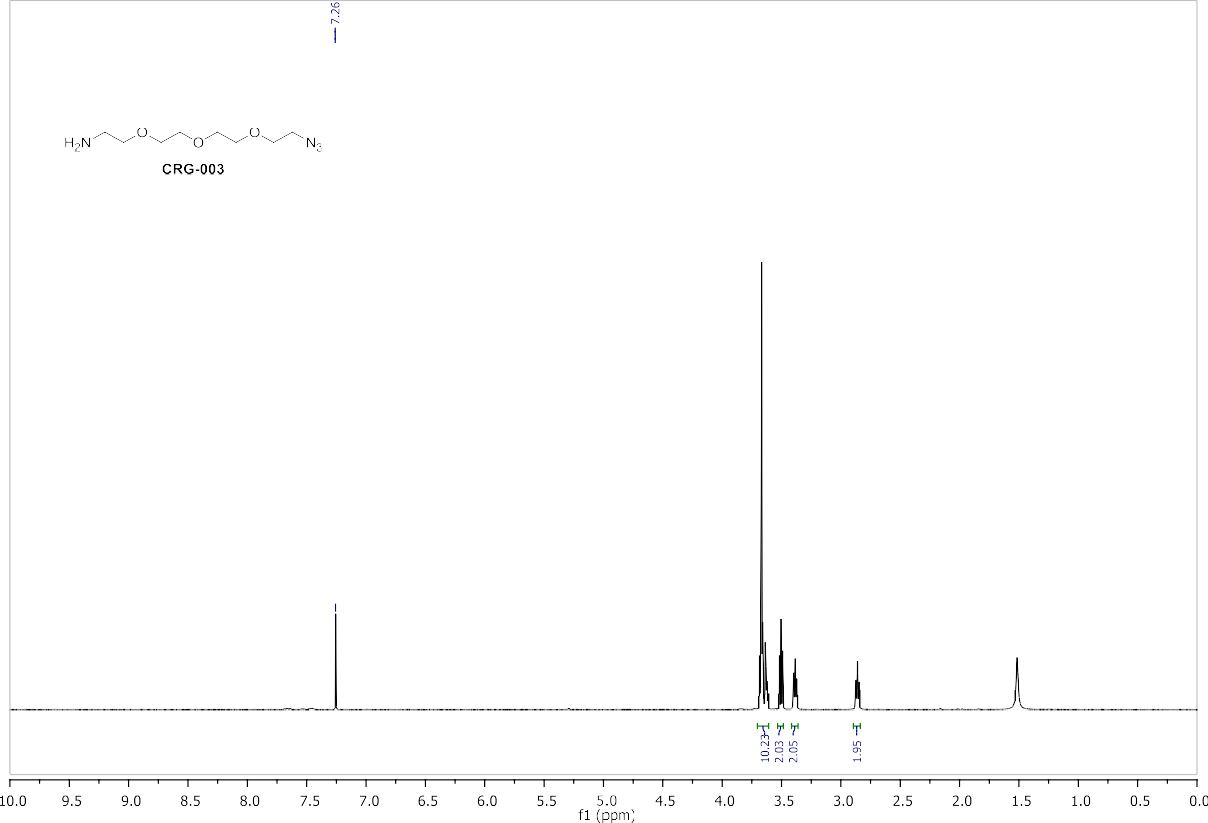


**a**


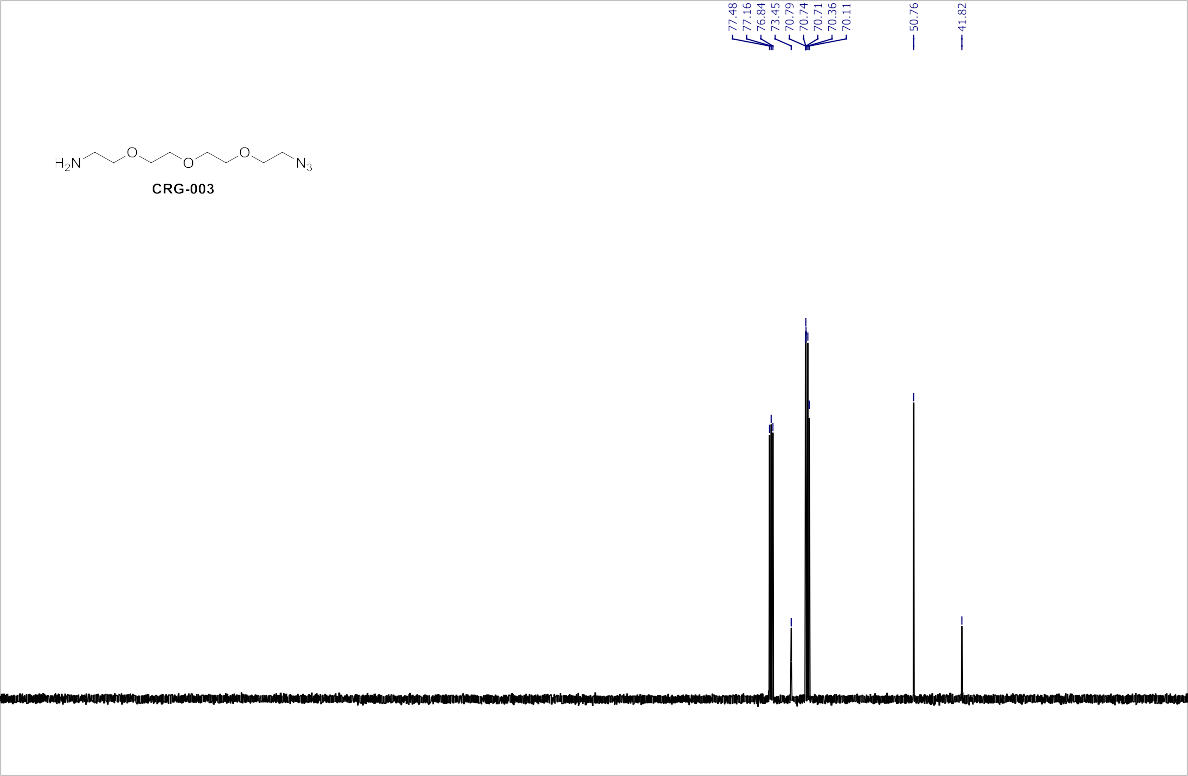


**b**

**Supplementary figure S12**


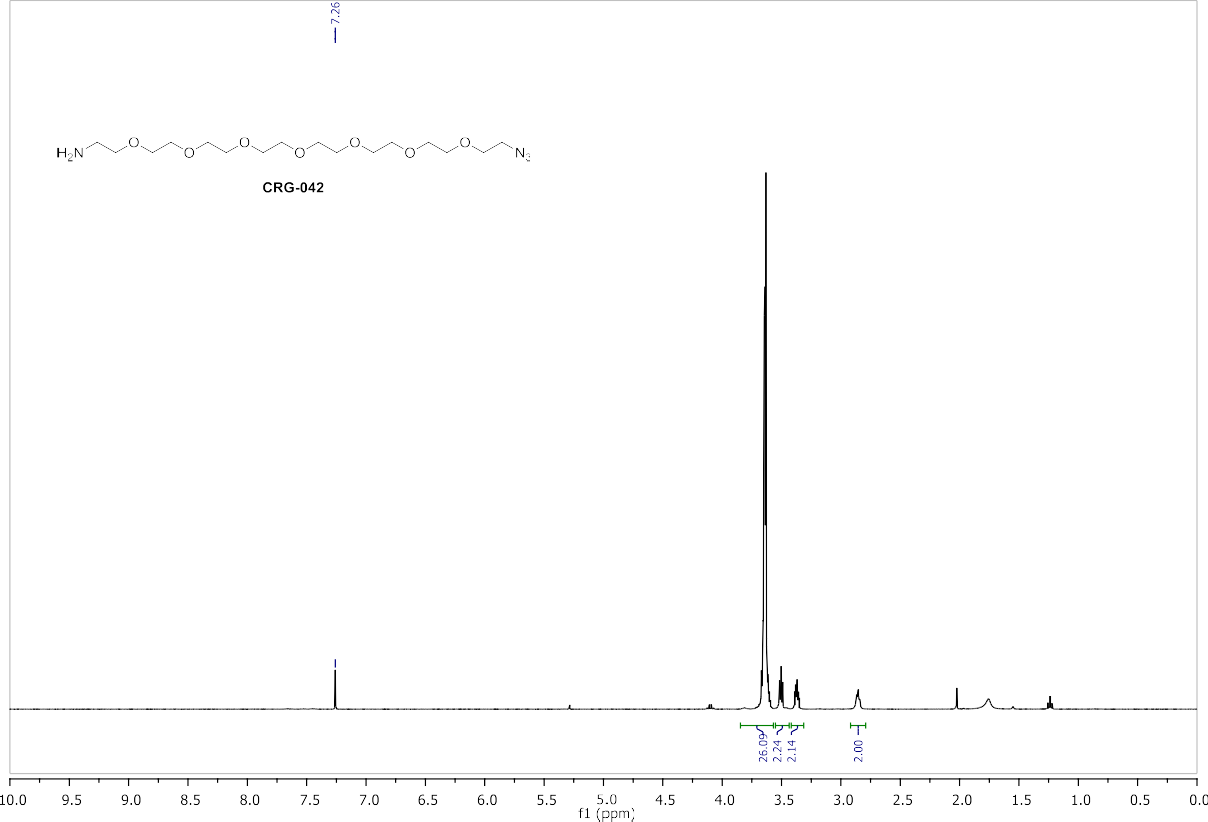


**a**


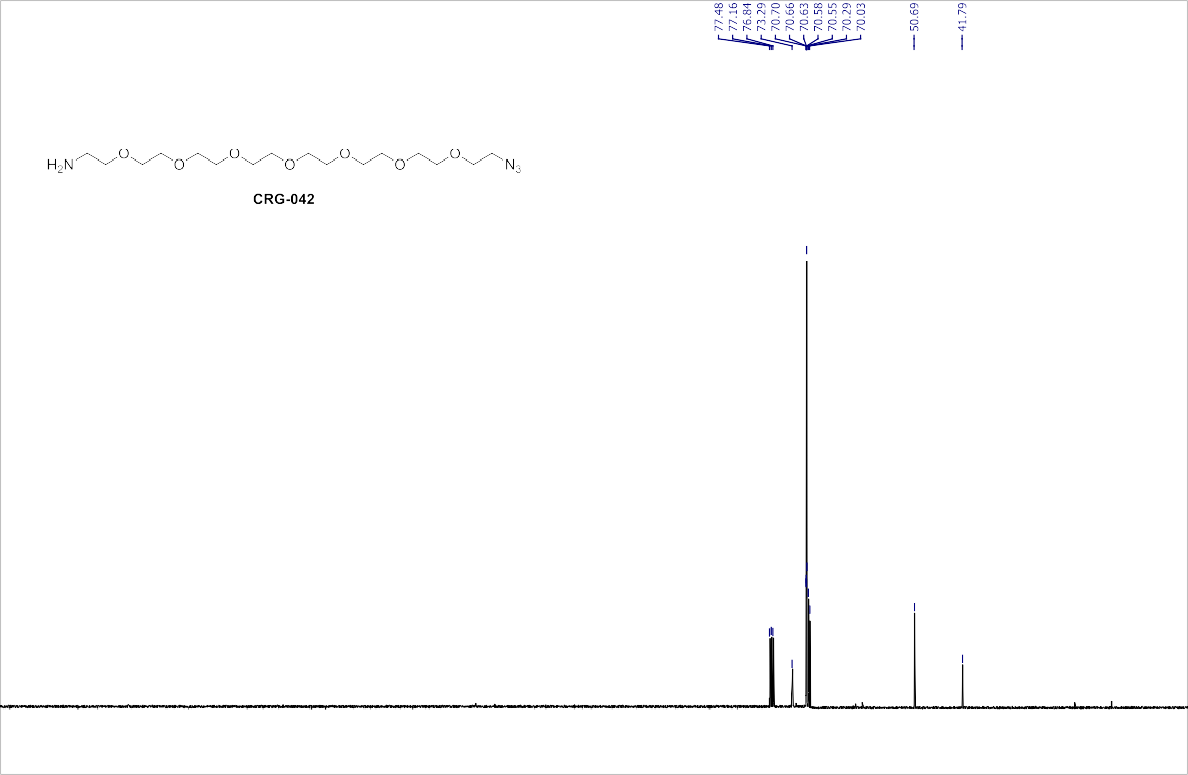


**b**

**Supplementary figure S13**


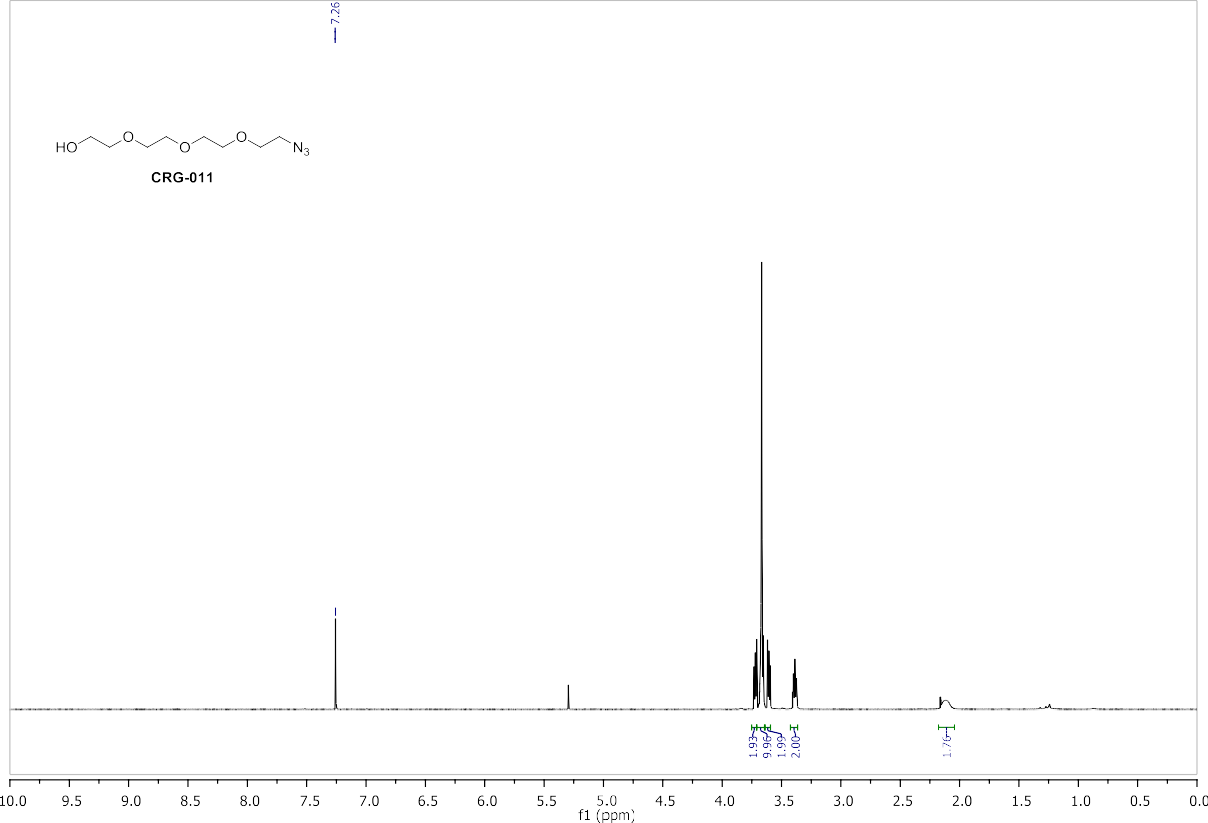


**a**


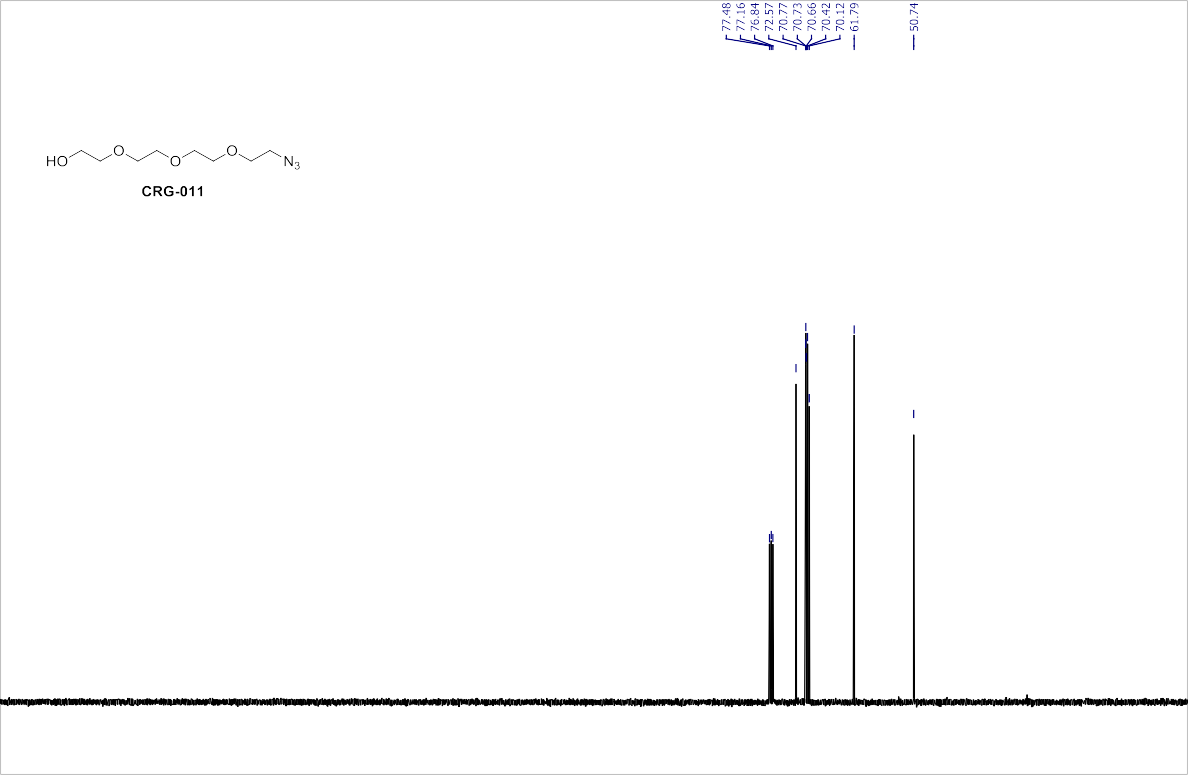


**b**

**Supplementary figure S14**


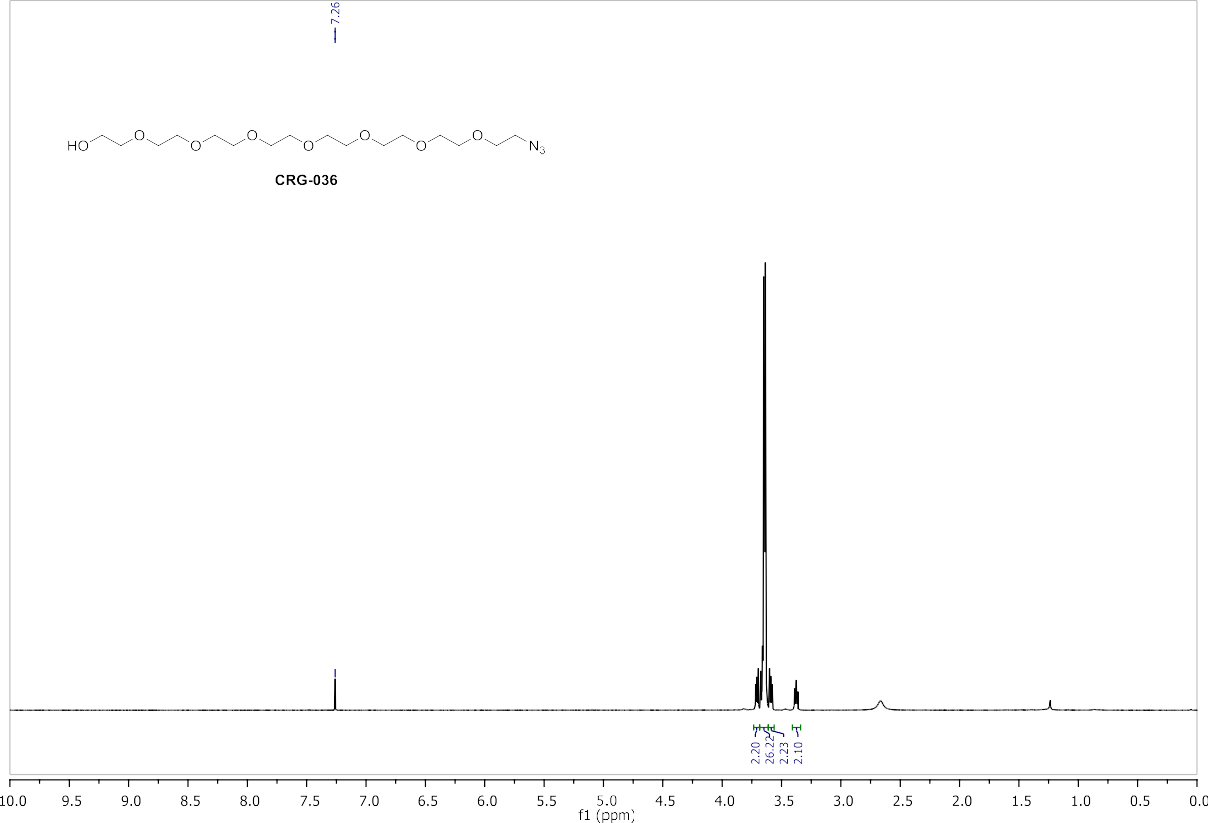


**a**


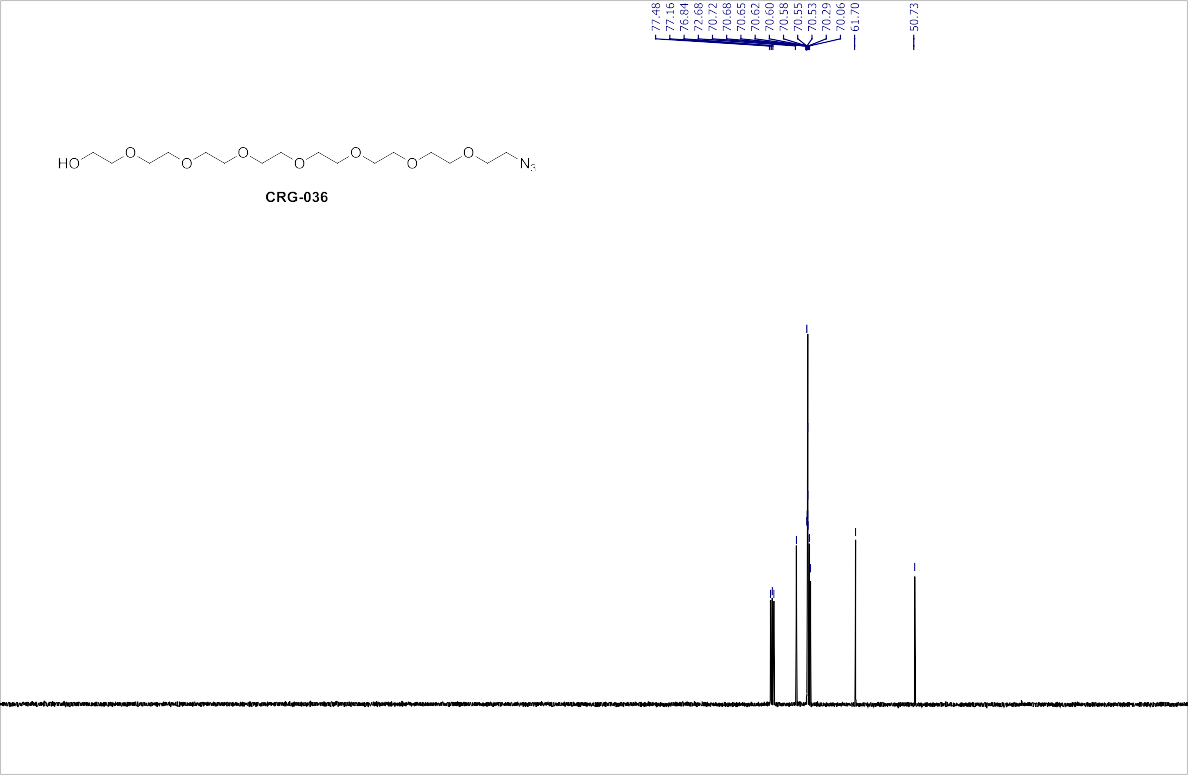


**b**

**Supplementary figure S15**


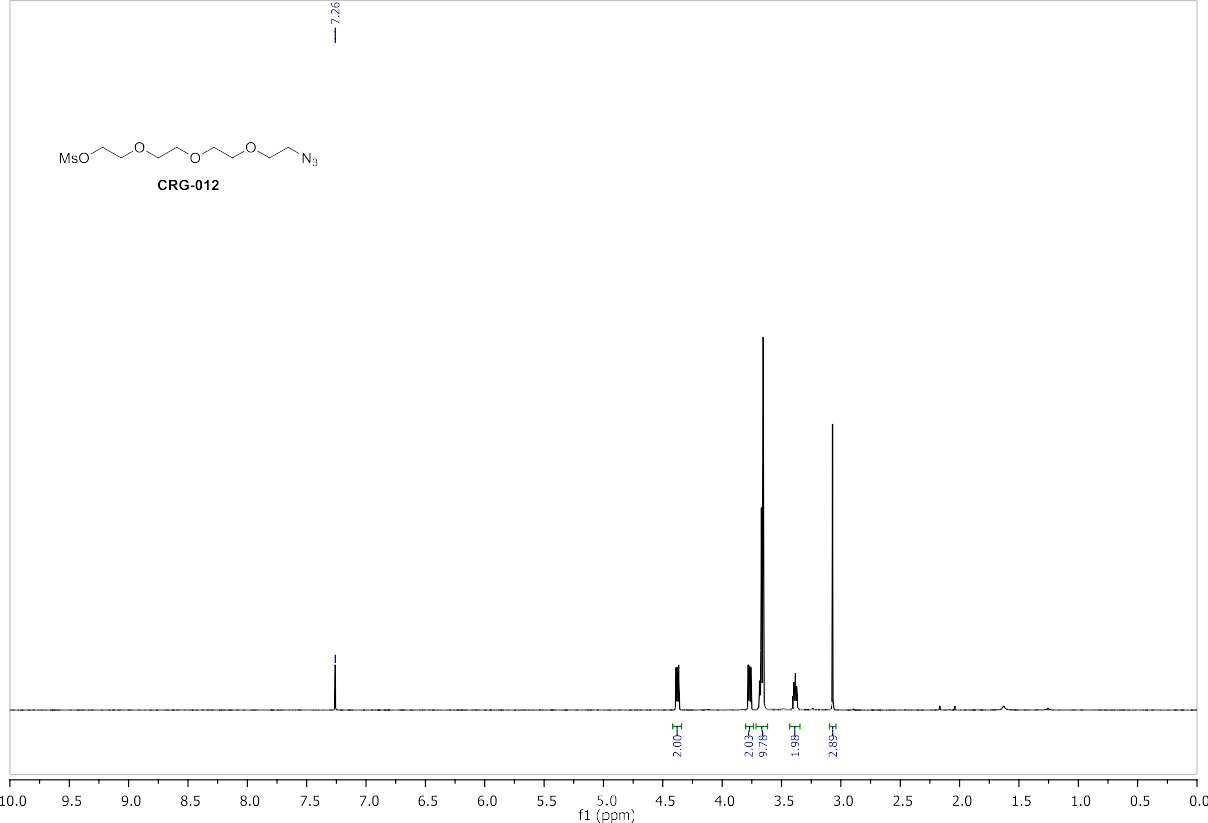


**a**


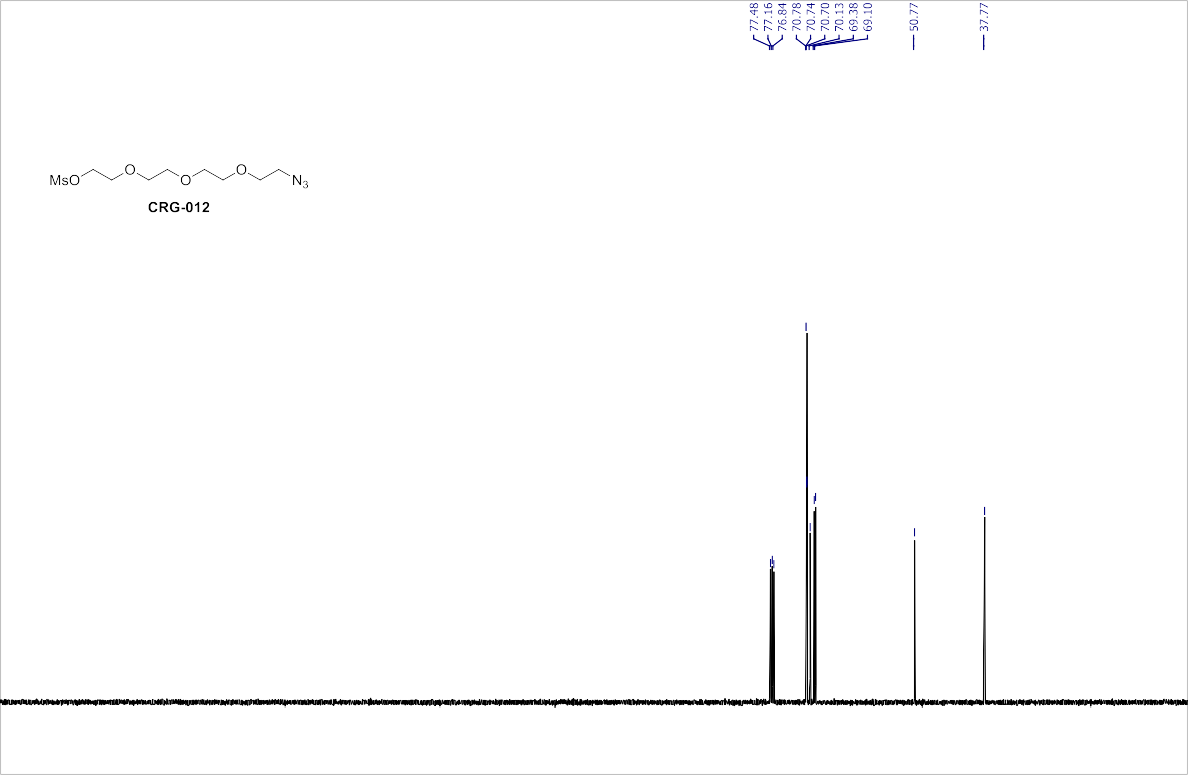


**b**

**Supplementary figure S16**


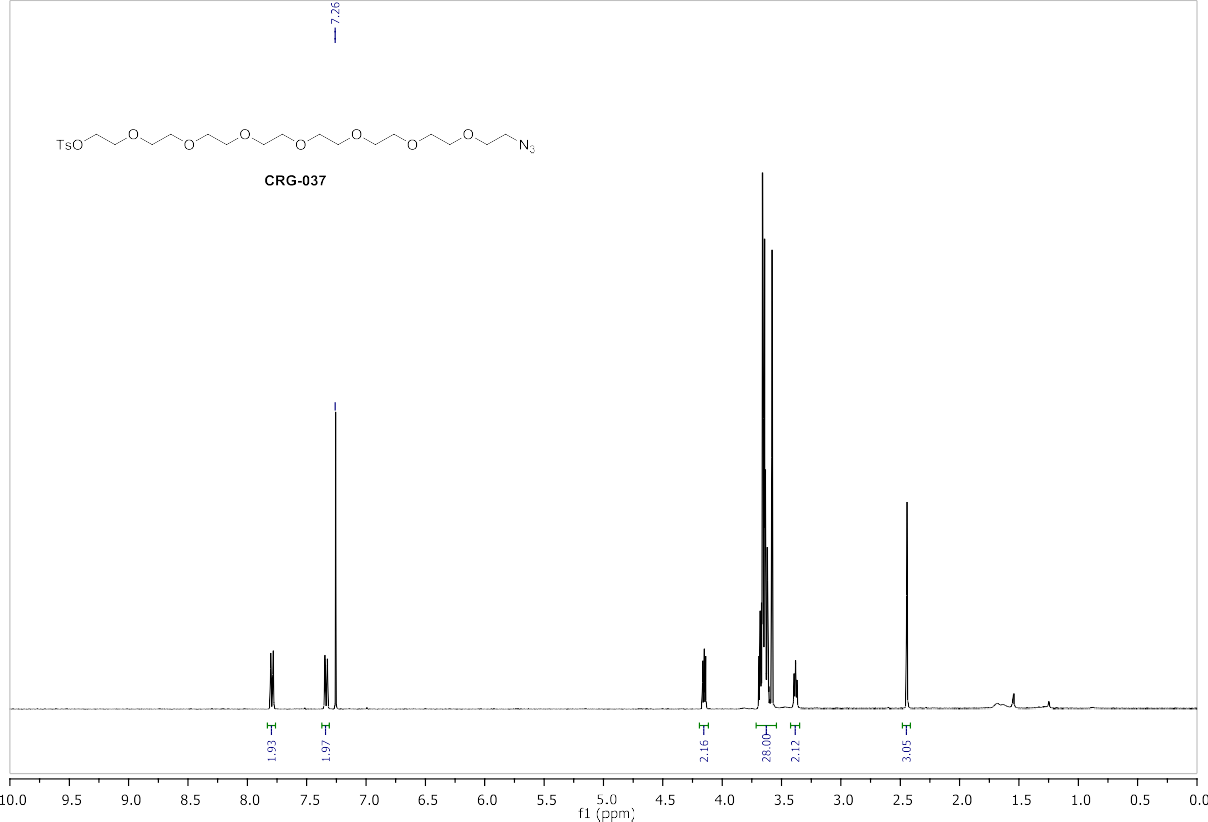


**a**


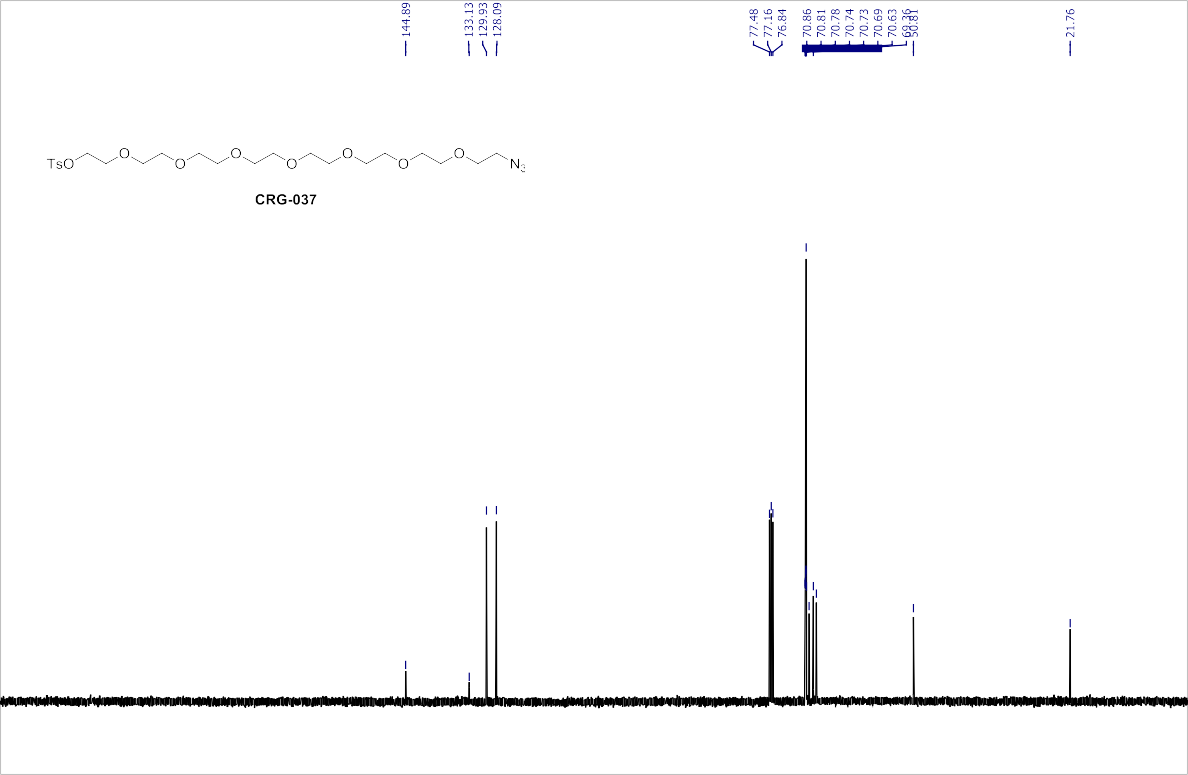


**b**

**Supplementary figure S17**


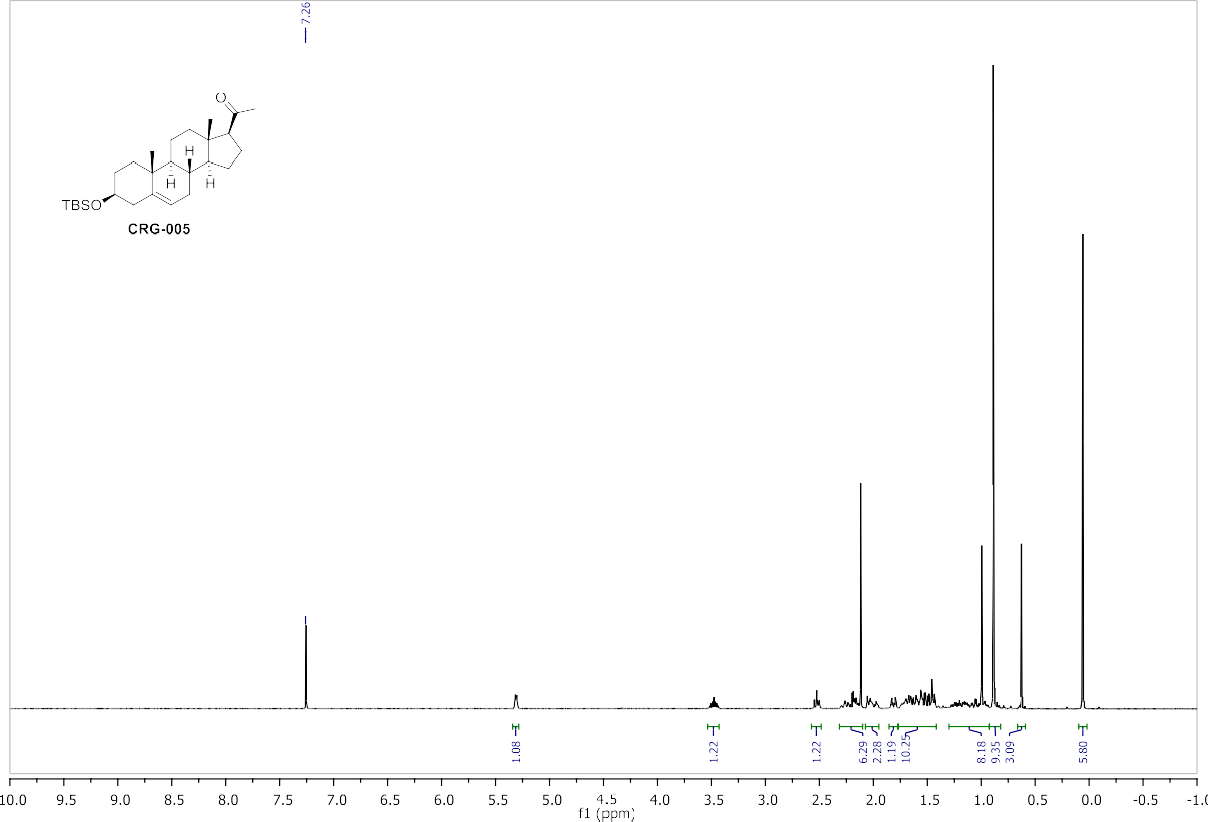


**a**


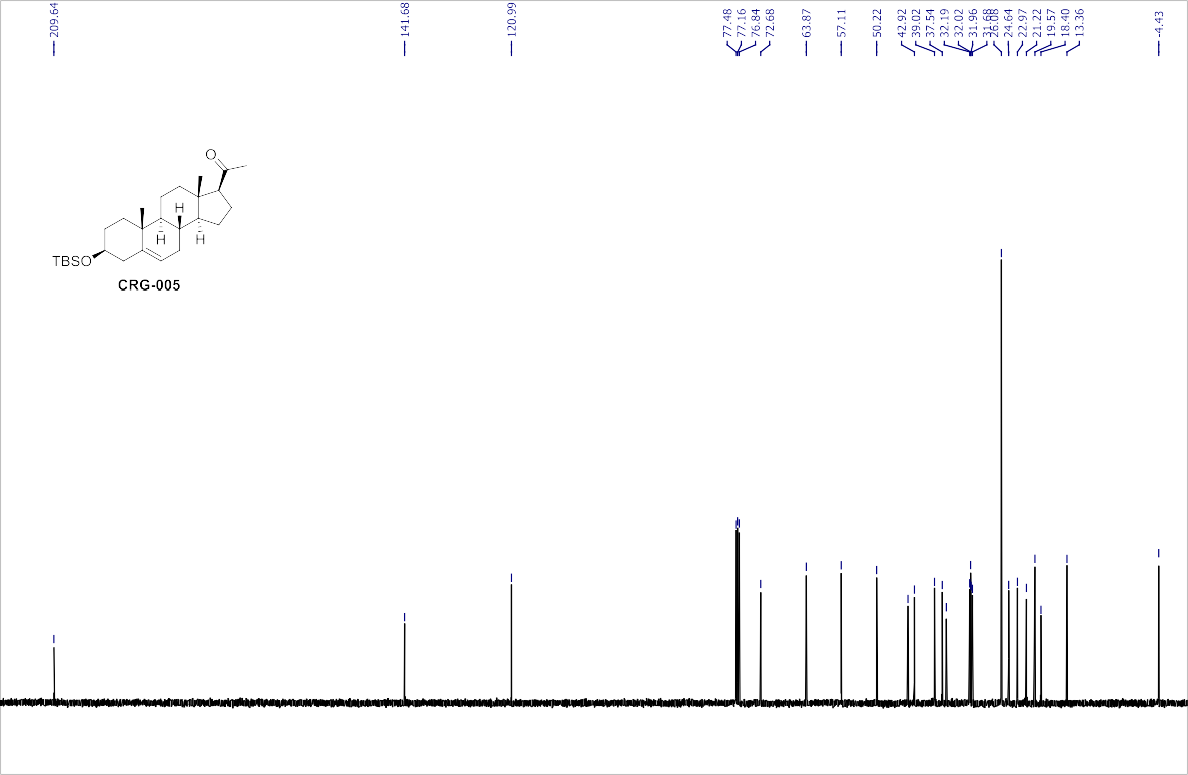


**b**

**Supplementary figure S18**


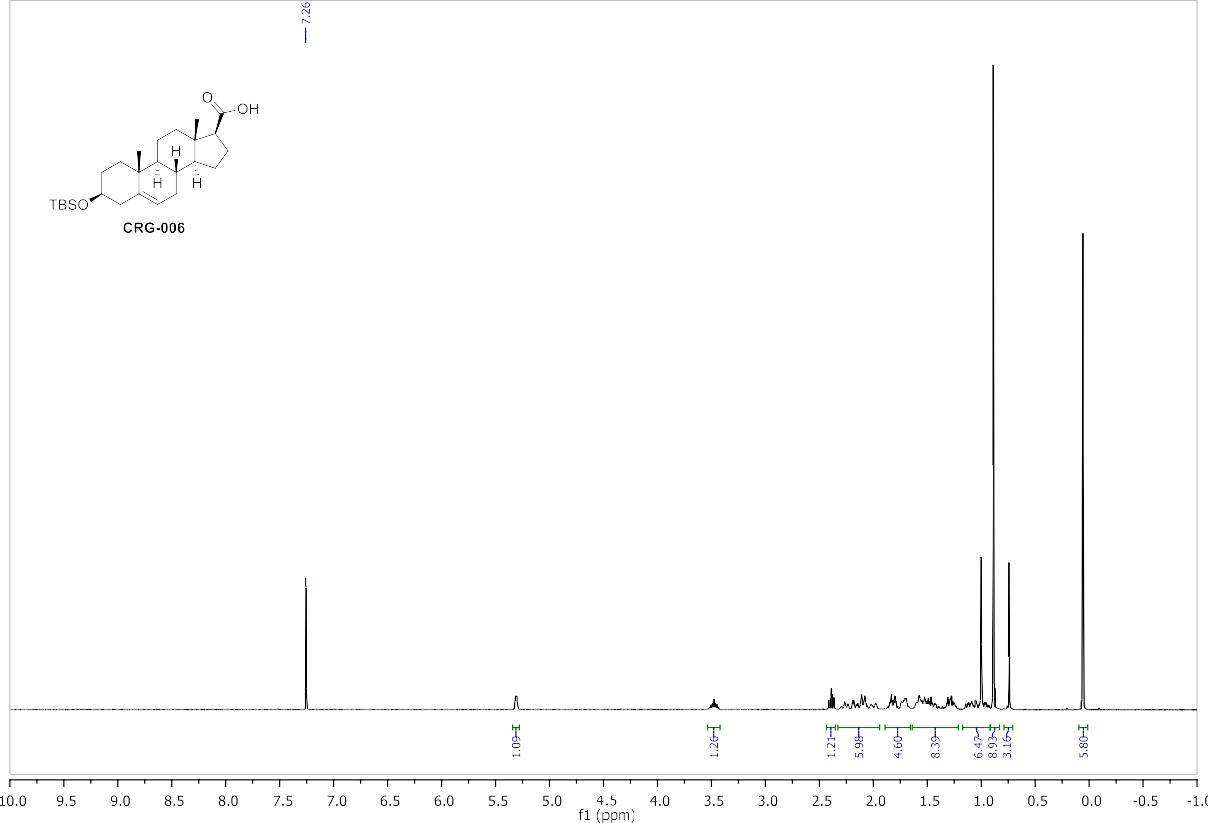


**a**


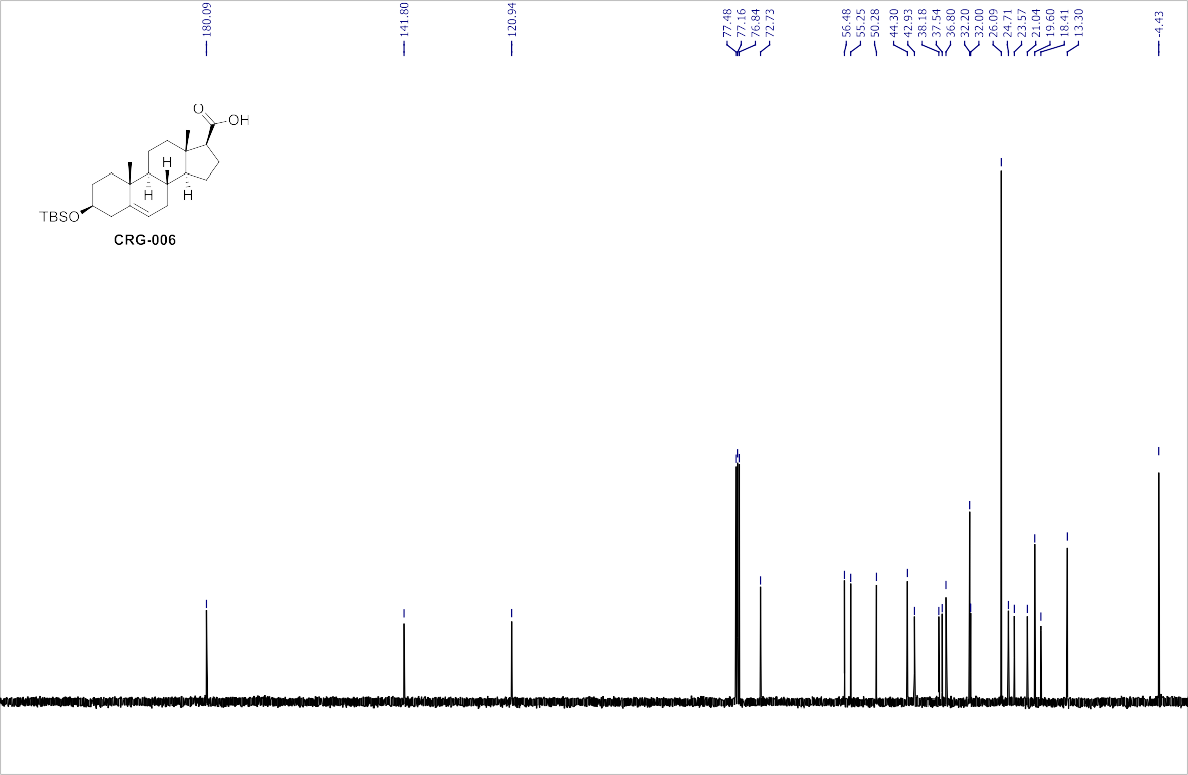


**b**

**Supplementary figure S19**


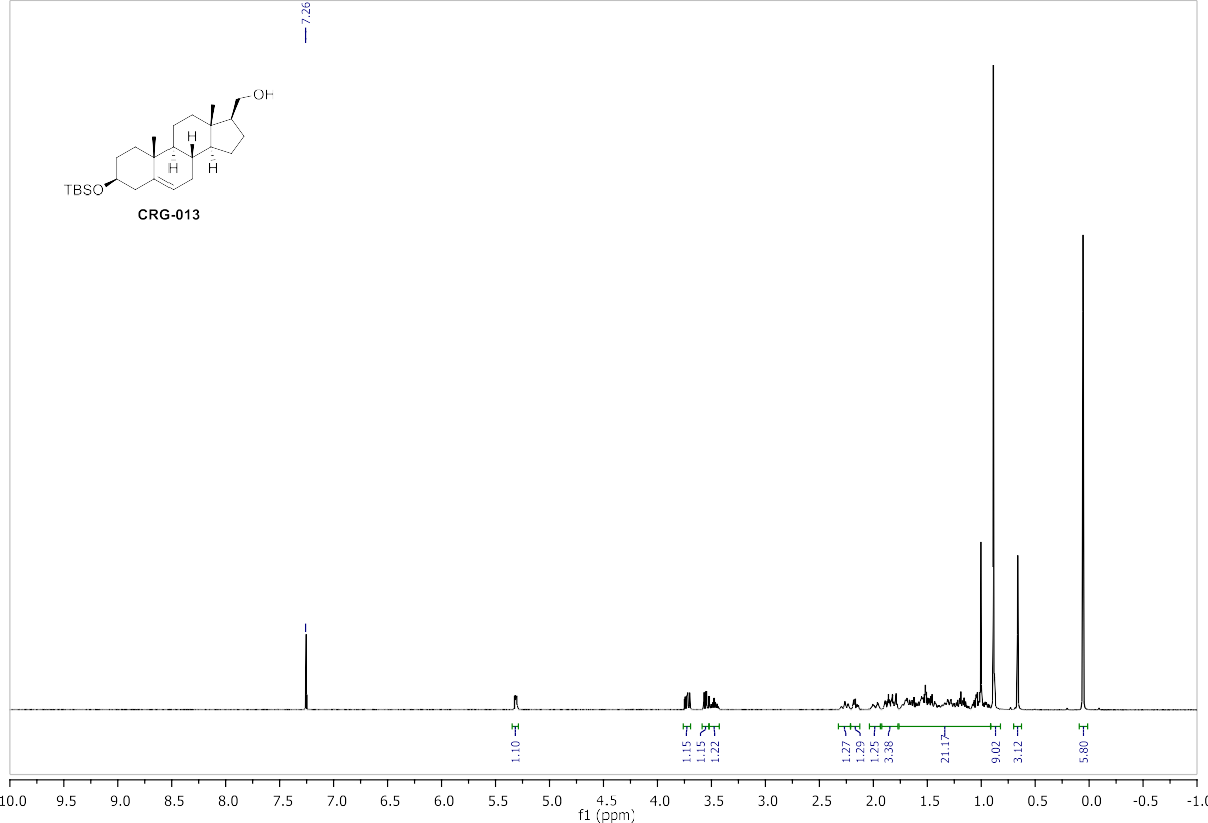


**a**


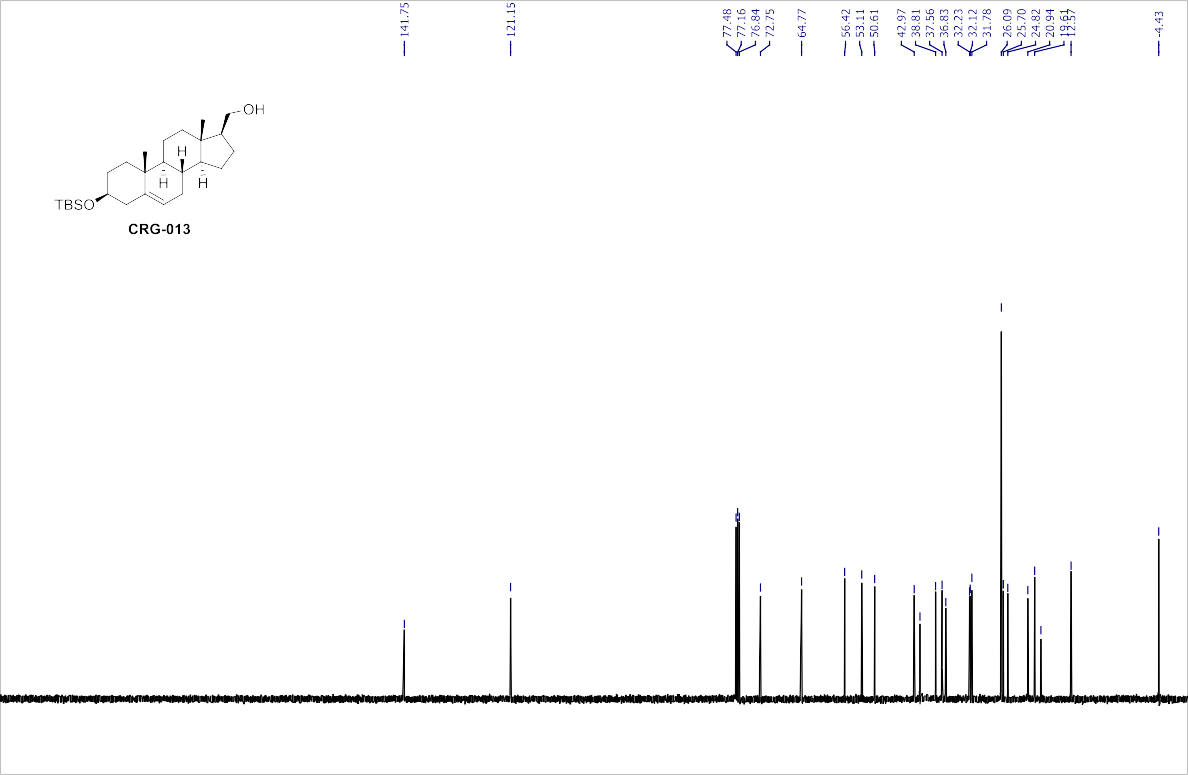


**b**

**Supplementary figure S20**


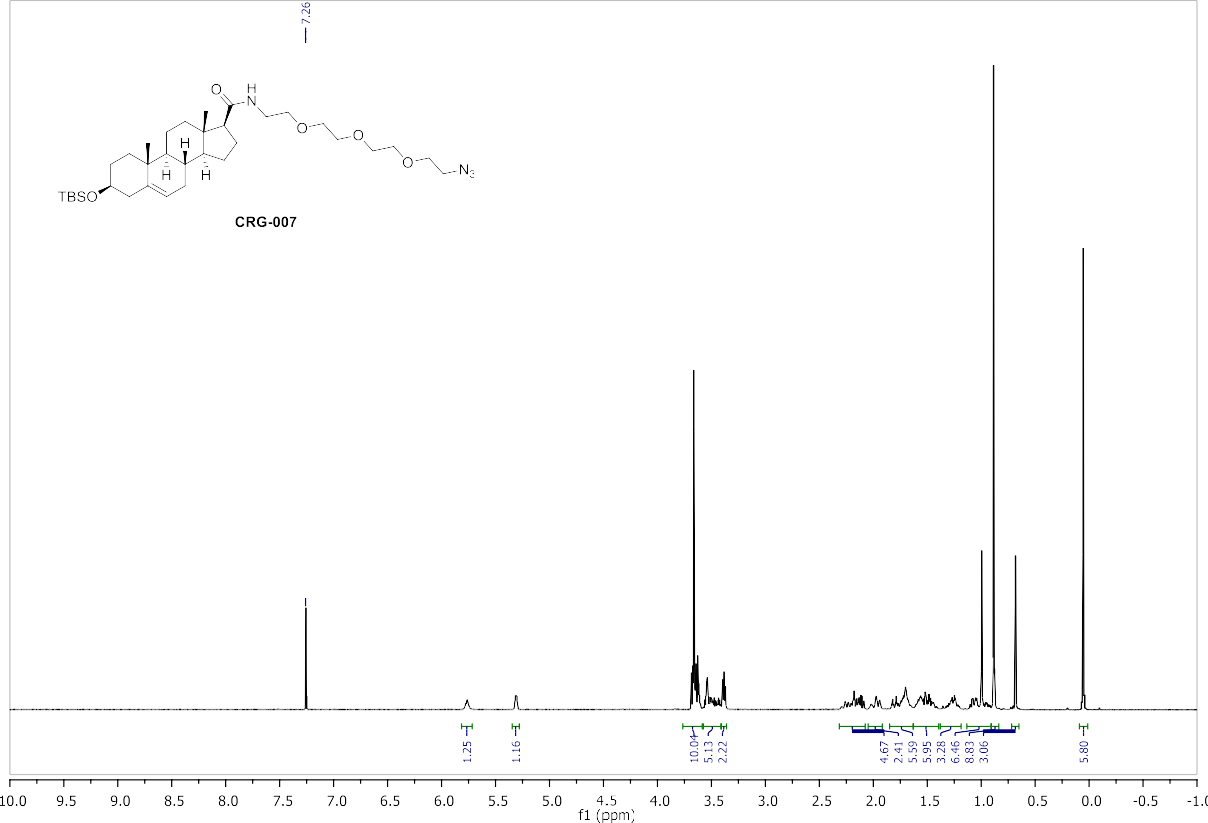


**a**


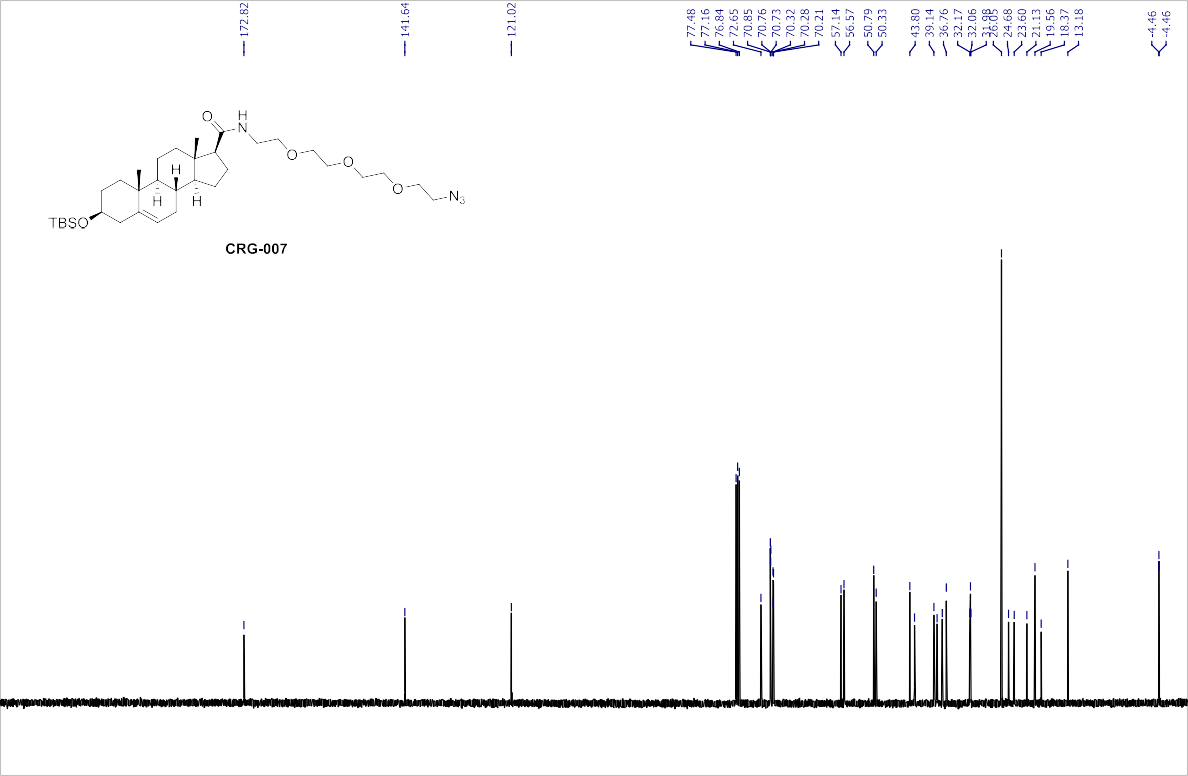


**b**

**Supplementary figure S21**


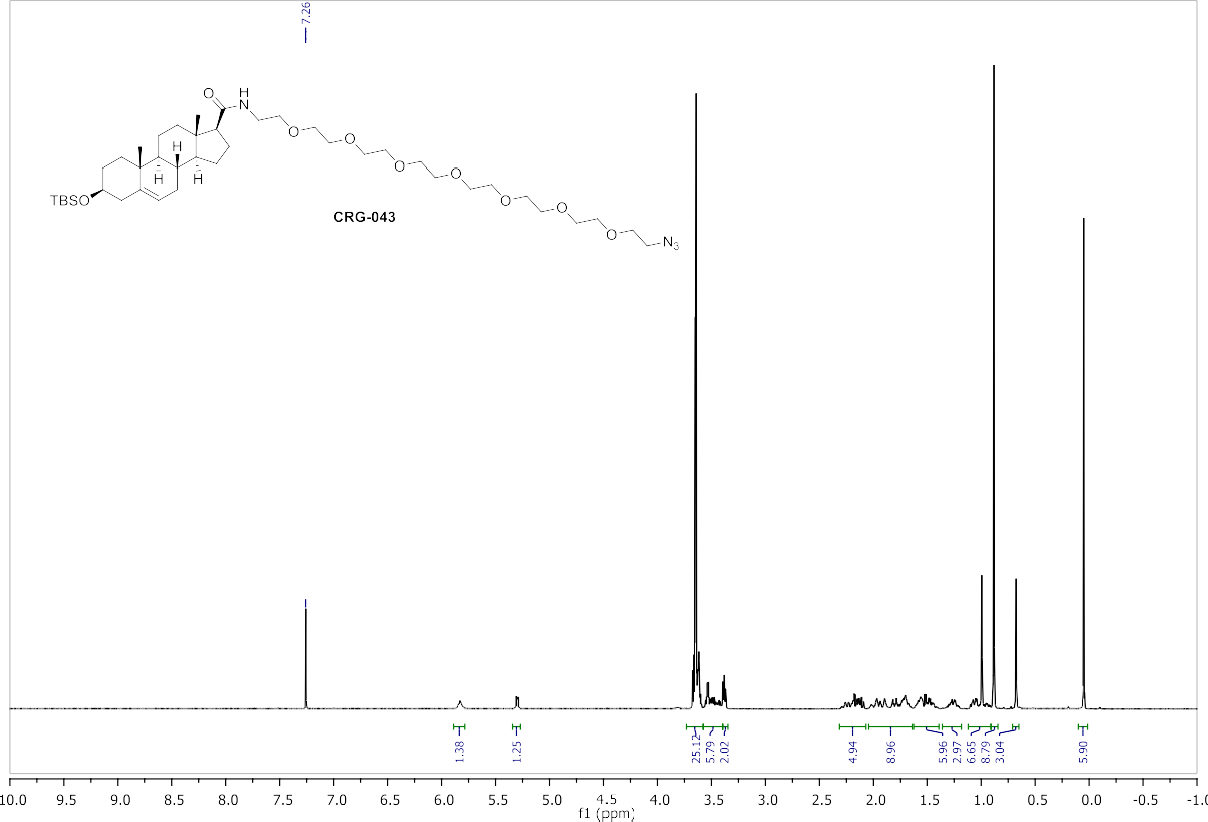


**a**


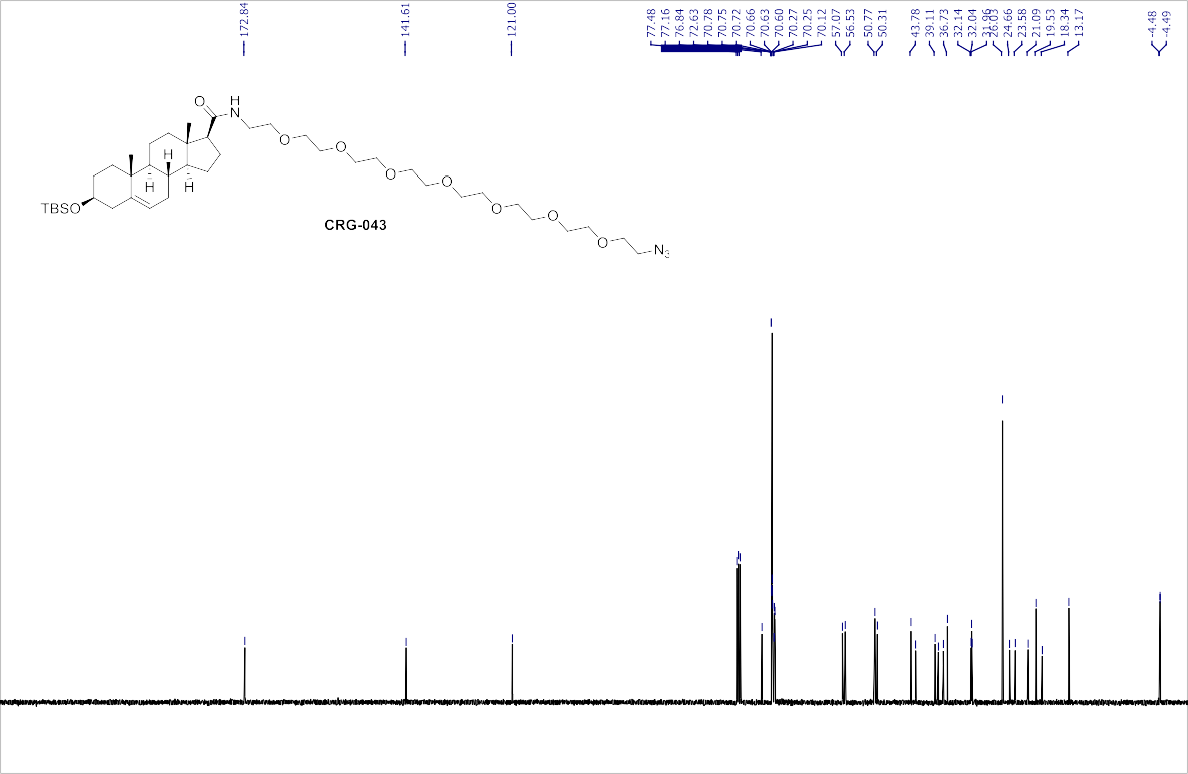


**b**

**Supplementary figure S22**


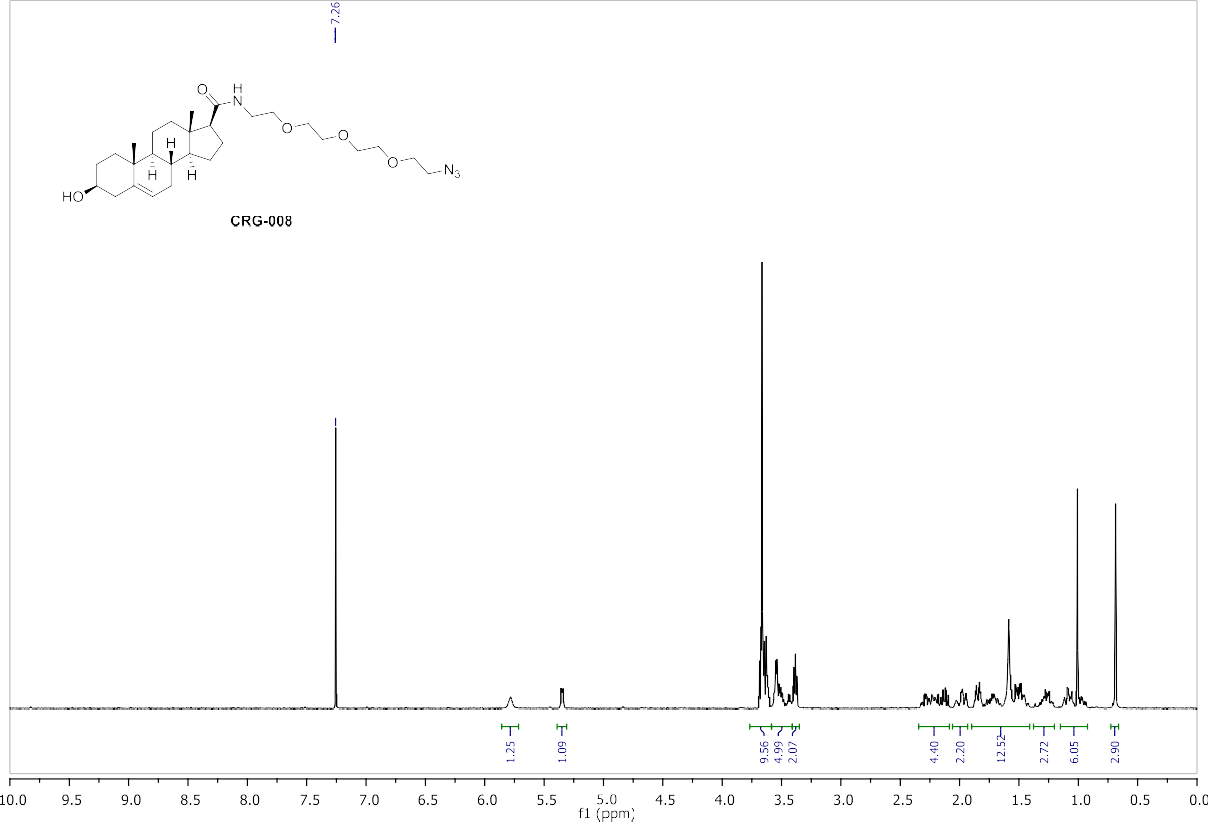


**a**


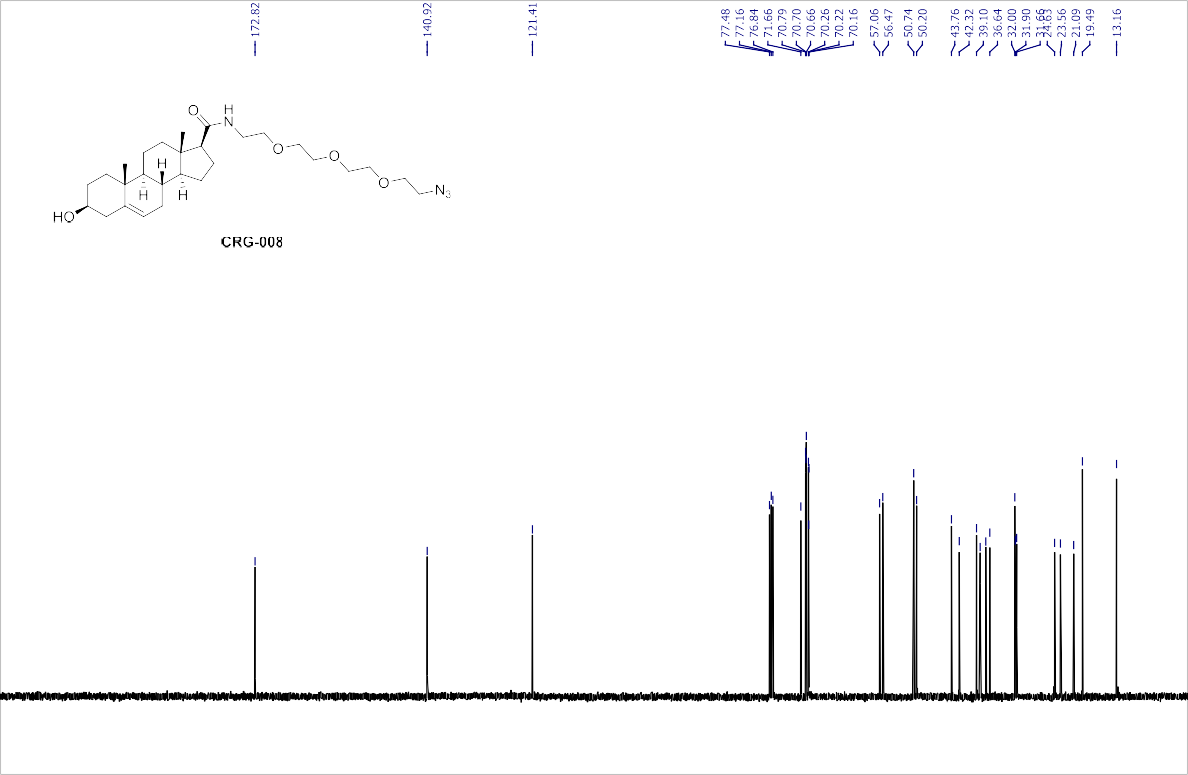


**b**

**Supplementary figure S23**


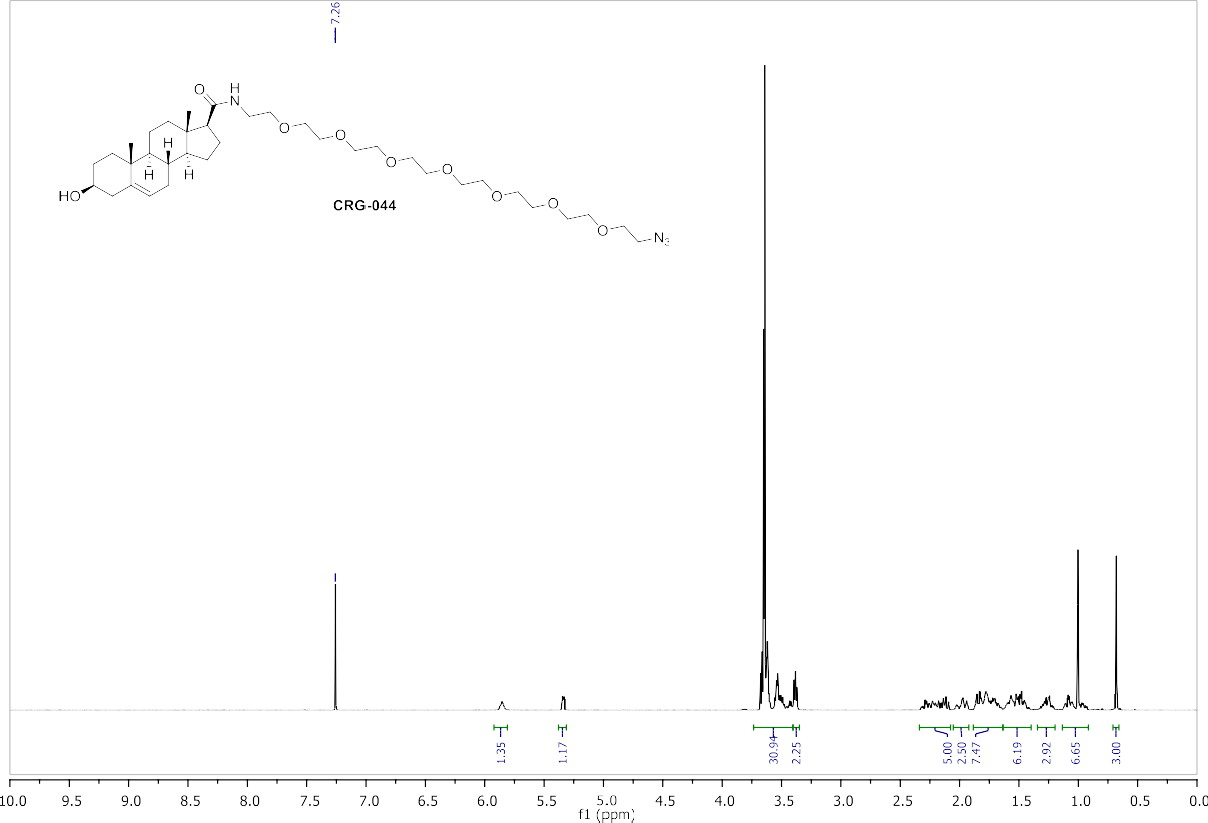


**a**


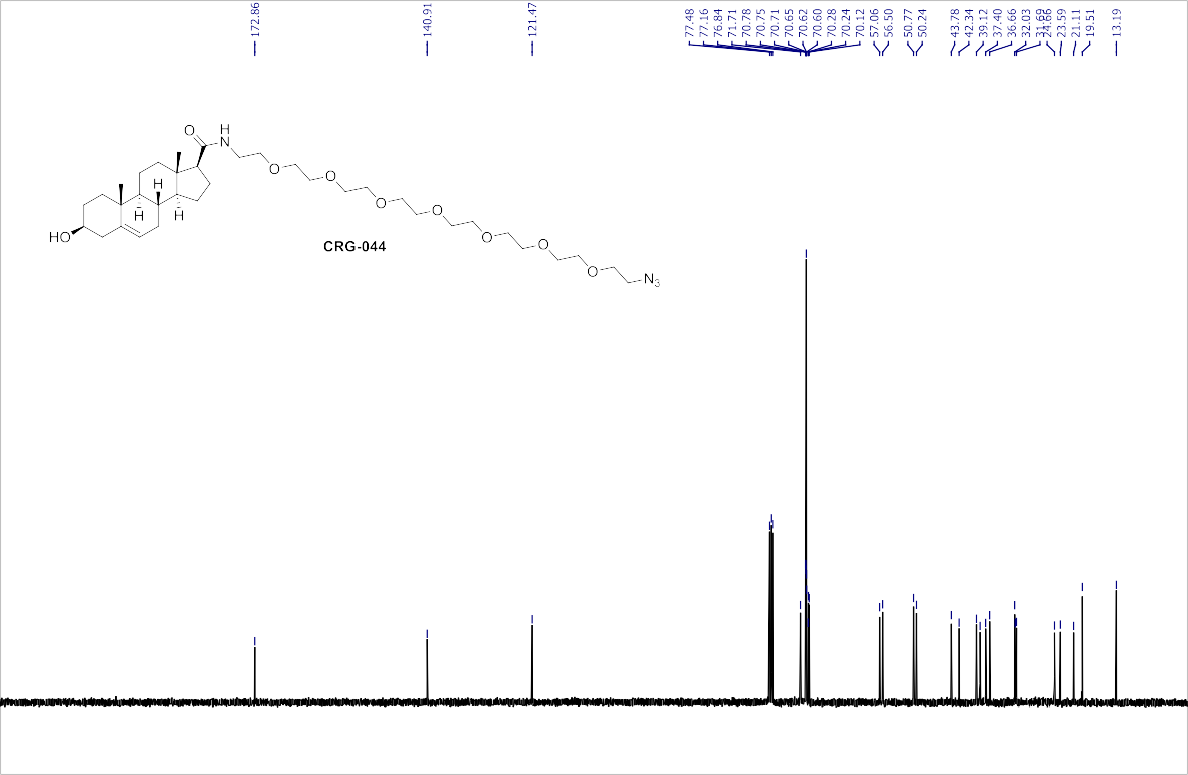


**b**

**Supplementary figure S24**


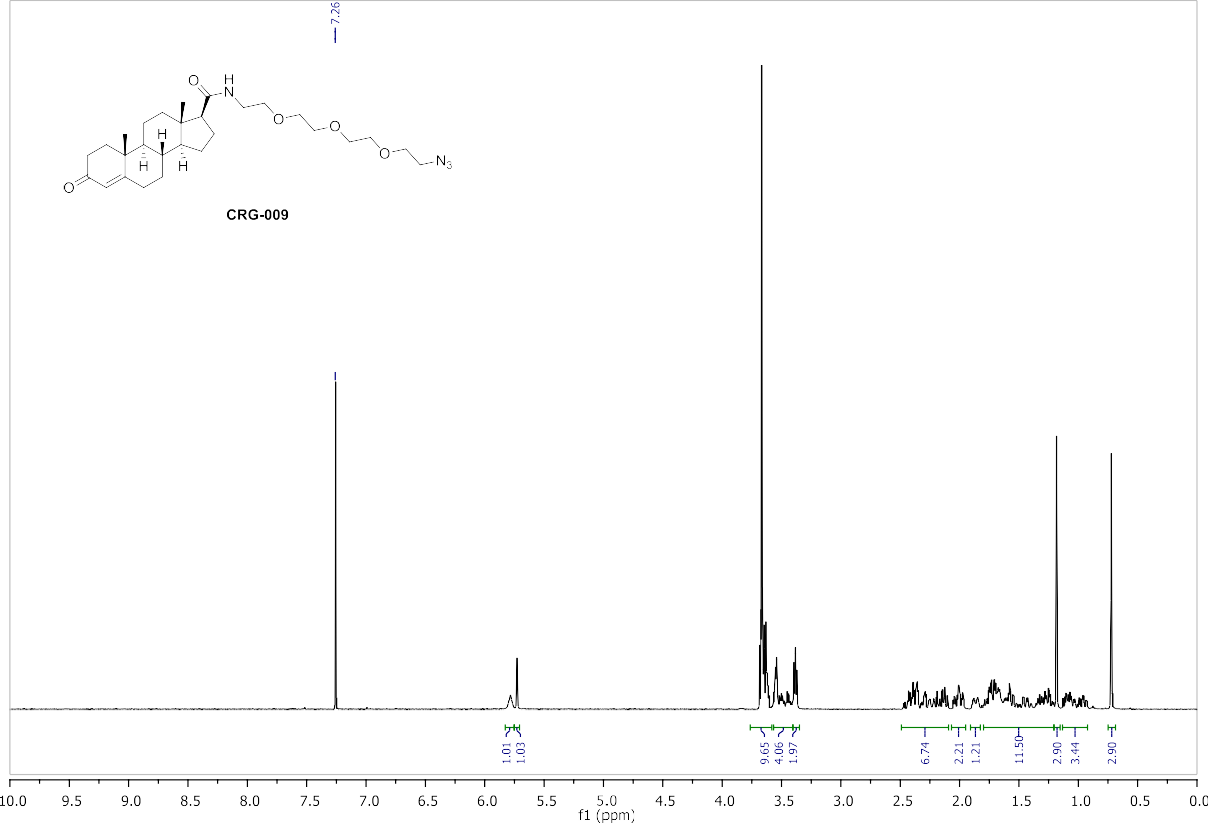


**a**


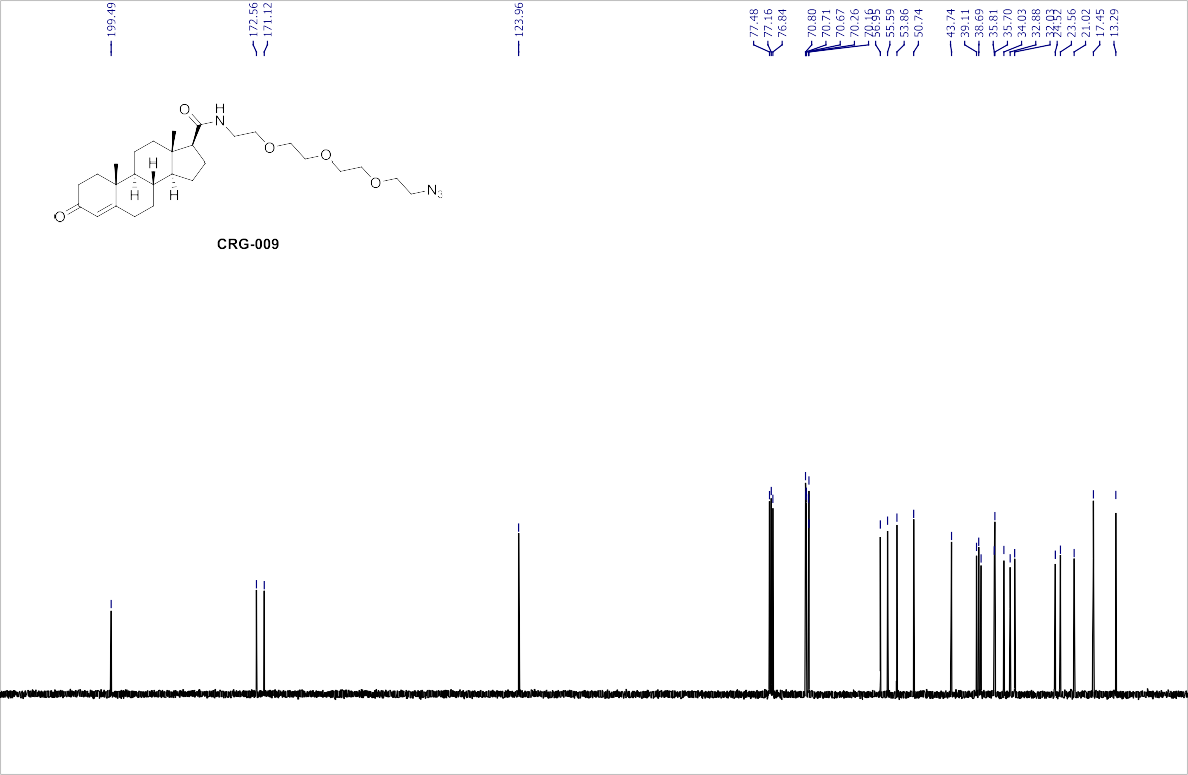


**b**

**Supplementary figure S25**


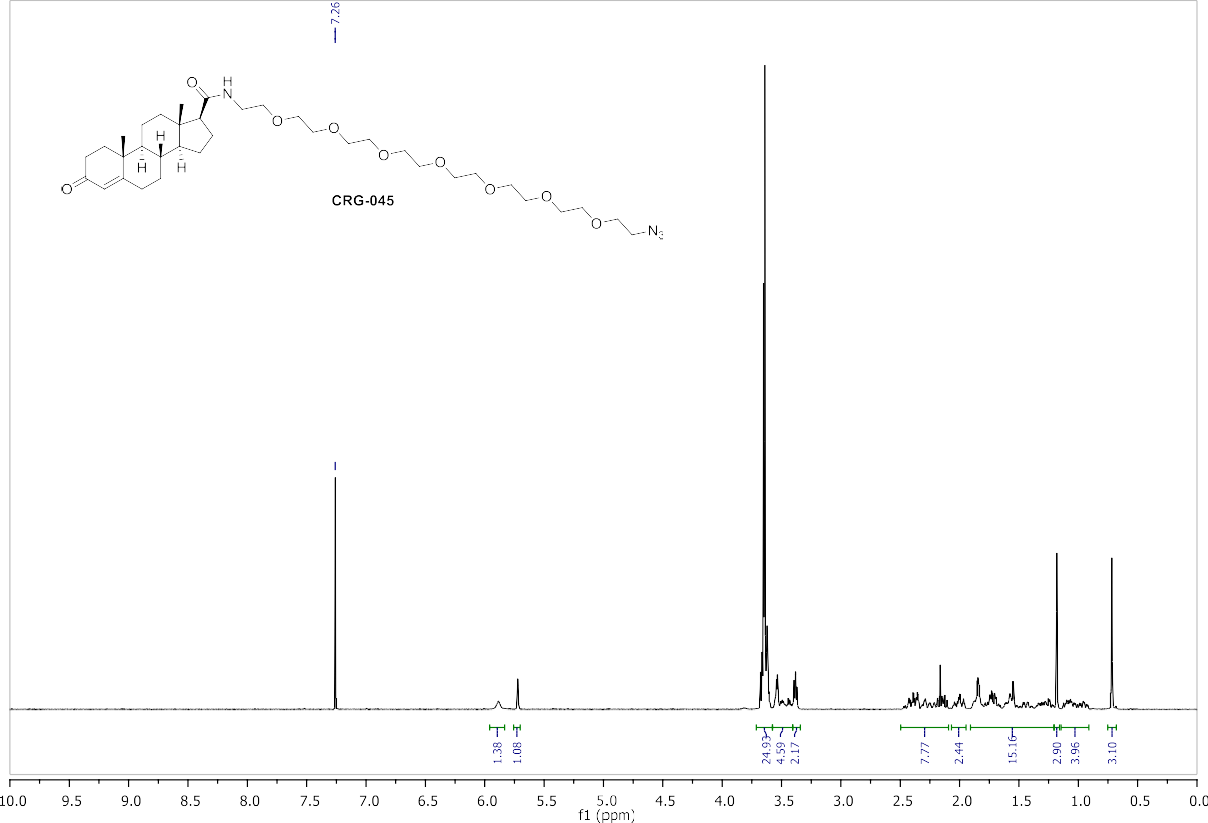


**a**


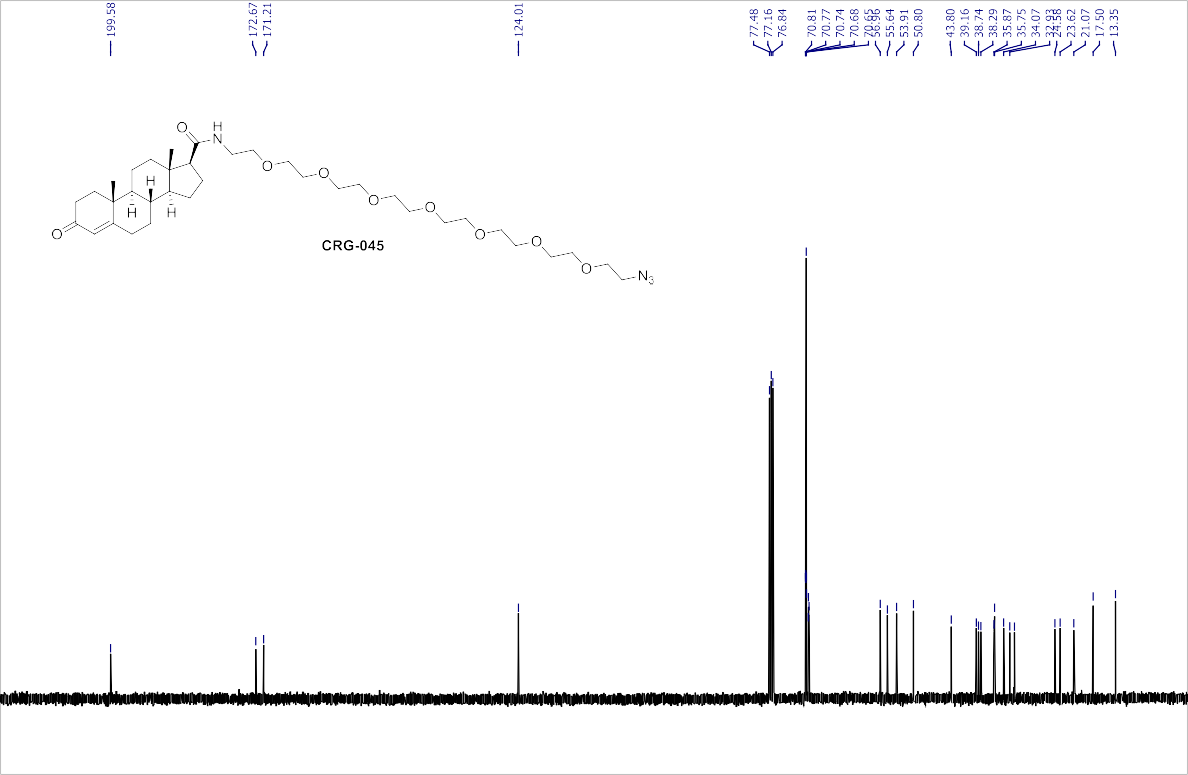


**b**

**Supplementary figure S26**


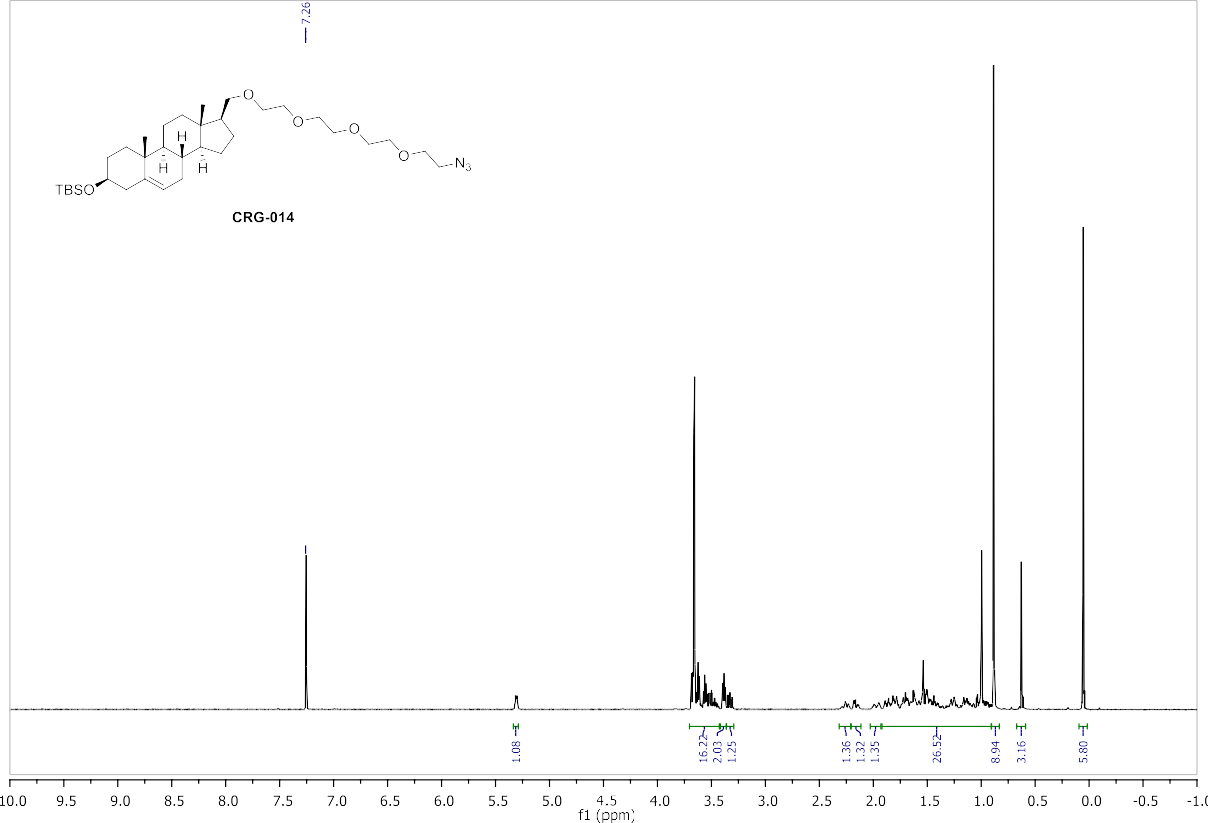


**a**


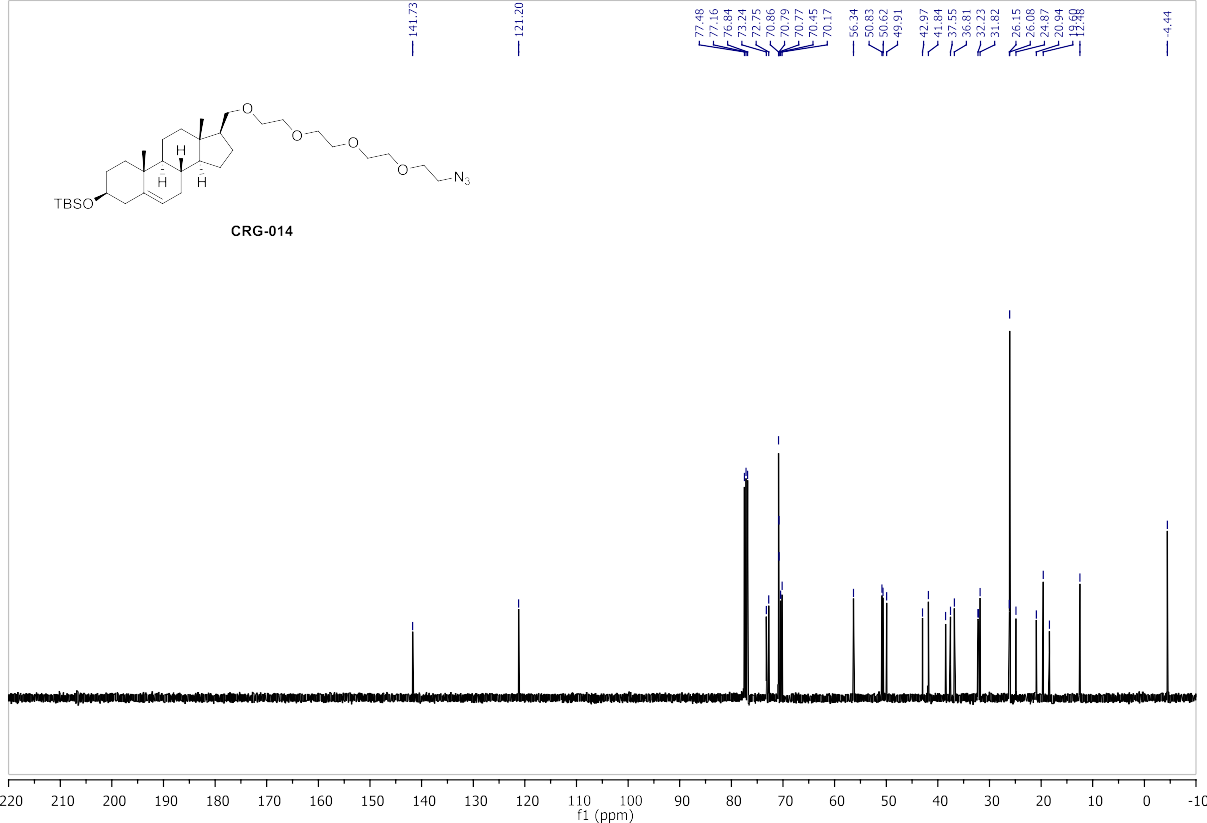


**b**

**Supplementary figure S27**

**a**

**b**

**Supplementary figure S28**

**a**

**b**

**Supplementary figure S29**

**b**

**a**

**Supplementary figure S30**

**a**

**b**

**Supplementary figure S31**

**a**

**b**

**Supplementary figure S32**

**a**

**b**

**Supplementary figure S33**

**a**

**b**

**Supplementary figure S34**

**a**

**b**

**Supplementary figure S35**

**a**

**b**

**Supplementary figure S36**

**a**

**b**

**Supplementary figure S37**

**a**

**b**

**c**

**Supplementary figure S38**

**a**

**b**

**c**

**Supplementary figure S39**

**a**

**b**

**c**

**Supplementary figure S40**

**Supplementary figure S41**

**a**

**b**

**c**
